# Poor efficacy of preemptive amoxicillin clavulanate for preventing secondary infection from *Bothrops* snakebites in the Brazilian Amazon: A randomized controlled clinical trial

**DOI:** 10.1371/journal.pntd.0005745

**Published:** 2017-07-10

**Authors:** Jacqueline A. G. Sachett, Iran Mendonça da Silva, Eliane Campos Alves, Sâmella S. Oliveira, Vanderson S. Sampaio, Fábio Francesconi do Vale, Gustavo Adolfo Sierra Romero, Marcelo Cordeiro dos Santos, Hedylamar Oliveira Marques, Mônica Colombini, Ana Maria Moura da Silva, Fan Hui Wen, Marcus V. G. Lacerda, Wuelton M. Monteiro, Luiz C. L. Ferreira

**Affiliations:** 1 Diretoria de Ensino e Pesquisa, Fundação de Medicina Tropical Dr. Heitor Vieira Dourado, Manaus, Brazil; 2 Escola Superior de Ciências da Saúde, Universidade do Estado do Amazonas, Manaus, Brazil; 3 Núcleo de Sistemas de Informação, Fundação de Vigilância em Saúde do Amazonas, Manaus, Brazil; 4 Faculdade de Medicina, Universidade Federal do Amazonas, Manaus, Brazil; 5 Núcleo de Medicina Tropical, Faculdade de Medicina, Universidade de Brasília, Brasília, Brazil; 6 Diretoria de Ensino e Pesquisa, Fundação Hospitalar de Hematologia e Hemoterapia do Amazonas, Manaus, Brazil; 7 Divisão de Desenvolvimento Científico, Instituto Butantan, São Paulo, Brazil; 8 Instituto de Pesquisas Leônidas & Maria Deane, FIOCRUZ, Manaus, Brazil; Institut de Recherche pour le Développement, BENIN

## Abstract

**Background:**

Secondary bacterial infections from snakebites contribute to the high complication rates that can lead to permanent function loss and disabilities. Although common in endemic areas, routine empirical prophylactic use of antibiotics aiming to prevent secondary infection lacks a clearly defined policy. The aim of this work was to estimate the efficacy of amoxicillin clavulanate for reducing the secondary infection incidence in patients bitten by *Bothrops* snakes, and, secondarily, identify risk factors for secondary infections from snakebites in the Western Brazilian Amazon.

**Methods and findings:**

This was an open-label, two-arm individually randomized superiority trial to prevent secondary infection from *Bothrops* snakebites. The antibiotic chosen for this clinical trial was oral amoxicillin clavulanate per seven days compared to no intervention. A total of 345 patients were assessed for eligibility in the study period. From this total, 187 accomplished the inclusion criteria and were randomized, 93 in the interventional group and 94 in the untreated control group. All randomized participants completed the 7 days follow-up period. Enzyme immunoassay confirmed *Bothrops* envenoming diagnosis in all participants. Primary outcome was defined as secondary infection (abscess and/or cellulitis) until day 7 after admission. Secondary infection incidence until 7 days after admission was 35.5% in the intervention group and 44.1% in the control group [RR = 0.80 (95%CI = 0.56 to 1.15; p = 0.235)]. Survival analysis demonstrated that the time from patient admission to the onset of secondary infection was not different between amoxicillin clavulanate treated and control group (Log-rank = 2.23; p = 0.789).Secondary infections incidence in 7 days of follow-up was independently associated to fibrinogen >400 mg/dL [AOR = 4.78 (95%CI = 2.17 to 10.55; p<0.001)], alanine transaminase >44 IU/L [AOR = 2.52 (95%CI = 1.06 to 5.98; p = 0.037)], C-reactive protein >6.5 mg/L [AOR = 2.98 (95%CI = 1.40 to 6.35; p = 0.005)], moderate pain [AOR = 24.30 (95%CI = 4.69 to 125.84; p<0.001)] and moderate snakebites [AOR = 2.43 (95%CI = 1.07 to 5.50; p = 0.034)].

**Conclusions/Significance:**

Preemptive amoxicillin clavulanate was not effective for preventing secondary infections from *Bothrops* snakebites. Laboratorial markers, such as high fibrinogen, alanine transaminase and C-reactive protein levels, and severity clinical grading of snakebites, may help to accurately diagnose secondary infections.

**Trial registration:**

Brazilian Clinical Trials Registry (ReBec): RBR-3h33wy; UTN Number: U1111-1169-1005.

## Author summary

*Bothrops* genus is responsible by 80–90% of the snakebites in the Brazilian Amazon, resulting in a subcutaneous and muscular lesion at the site of bite, which many times evolve to local complications, mostly secondary bacterial infections. In this region, late medical assistance is common and probably contributes to the high complication rates related to local necrosis and secondary bacterial infections, which can lead to permanent function loss and disabilities. Even with this high frequency, routine empirical use of antibiotics aiming to prevent secondary infection lacks a clearly defined protocol. In this work, we estimated the efficacy of amoxicillin clavulanate for reducing the secondary infection incidence in patients bitten by *Bothrops* snakes, and, identified factors related to secondary infections from snakebites. Amoxicillin clavulanate was not effective for preventing secondary infections from *Bothrops* snakebites, probably because of the resistance to β-lactam antibiotics in bacteria species commonly found infecting the snakebite site. This finding highlights the need of previous knowledge of the secondary infections epidemiology as a cornerstone in the preemptive antibiotics trials in snakebites. Laboratorial markers, such as high fibrinogen, alanine transaminase and C-reactive protein levels, and severity clinical grading of snakebites, may help to accurately diagnose secondary infections.

## Introduction

*Bothrops* snakebites result in a subcutaneous and muscular lesion at the site of bite, which many times evolve to local complications [1–5]. In 2015, the Brazilian Ministry of Health recorded 18,741 snakebites across the country [6]. In this country, a higher incidence is observed in the Brazilian region (37 cases/100.000 inhabitants). These values could be higher in remote areas of the Amazon because of the considerable case underreporting [7]. Snakebites are recorded mostly from remote rural or riverine areas, from where patients’ rescue to the health units is made exclusively by boat transportation, lasting several hours or even days [8–10]. Thus, late medical assistance is common and probably contributes to the high complication rates related to local necrosis [10–12] and secondary bacterial infections [10–15], that can lead to permanent function loss and disabilities [3,10,16–19]. It was suggested that secondary bacterial infections from snakebites are related to the oral and fangs microbiota of the perpetrating snake [20–26]. Besides, traditional treatment also contributes for the emergence of secondary infections, such as tourniquet use, local alternative medicines, incision and suction of the bite site [8,18,27]. These factors increase the development of expressive forms of secondary infection, which has been primarily diagnosed with identification of cellulitis or abscesses [12,13,22].

In the Brazilian Amazon, *Bothrops atrox* is the most important venomous snake, causing 80–90% of the snake envenomings [28], Despite the wide geographic distribution in the Amazon, *B*. *atrox* venoms share the same family of toxins, as PIII and PI snake venom metalloproteinase, phospholipase A2, serine proteinase, cysteine-rich secretory protein, L-amino acid oxidase and C-type lectin-like [29,30] and are characterized by coagulant, hemorrhagic and proteolytic or acute inflammatory activities [31–33]. Spontaneous systemic bleeding and acute renal failure are common systemic complications from *Bothrops* envenomings [13,34]. Local envenomation range from a painless reddened injury to intense pain and swelling at the site of bite, starting minutes after the event. Enlargement of the regional lymph nodes draining the site of bite and bruising can also be observed some hours after bite, especially if patient delayed in reaching a health service [33,34][34][33][33][33][33]. In the first 24 hours, blistering and tissue necrosis may be evident. Cellulitis or abscess occurs mostly in the moderate or severe cases, generally as a polymicrobial infection. Gram-negative bacteria have been implicated in secondary bacterial infection, which frequency may vary according to region [35]. In Manaus, secondary bacterial infections were observed in around 40% of the *Bothrops* snakebites [13].

Several antimicrobial schemes were suggested for the treatment of secondary infections, but in general these recommendations were not based on good evidences from clinical trials [36,37]. For example, ampicillin/cephalosporin/cloxacillin [14,27], ciprofloxacin [15,22] and clindamycin [36,38,39] were previously used for secondary bacterial infections resulted from snakebites, with variable effectiveness. In the Amazon, basic information about the bacterial agents responsible by the wound infection is still lacking since secondary infection diagnosis is mostly based only from clinical features without microbiological confirmation. Although common in endemic areas, routine empirical prophylactic use of antibiotics aiming to prevent secondary infection lacks a clearly defined policy, leading to wasteful inappropriate antibiotic use, which is costly and may promote bacterial antibiotic resistance [22,40]. Preemptive treatment efficacy of oral chloramphenicol monotherapy in *Bothrops* snakebites [41] and intravenous chloramphenicol plus gentamicin in *Crotalus* snakebites [42] showed no statistical difference between patients treated and untreated groups. Nowadays, however, the Infectious Diseases Society of America (IDSA) guidelines for diagnosis and management of skin and soft-tissue infections indicate amoxicillin clavulanate to reduce complications by prevention of secondary infection from animal bites [38,39]. Evidence supporting this recommendation came from a clinical trial carried out with patients bitten by dogs [43], but efficacy of this regimen is still not available in snakebites.

The aim of this work was to estimate the efficacy of amoxicillin clavulanate for reducing the secondary infection incidence in patients bitten by *Bothrops* snakes, and, secondarily, identify associated factors for secondary infections from snakebites in the Western Brazilian Amazon.

## Methods and materials

### Ethics statement

Ethical approval was obtained from the *Fundação de Medicina Tropical Doutor Heitor Vieira Dourado* (FMT-HVD) (approval number 492.892/2013). Written informed consent was obtained from all participants prior to randomization. This study was registered in the Brazilian Clinical Trials Registry (ReBec): RBR-3h33wy and UTN Number: U1111-1169-1005.

### Study design and participants

This was an open-label, two-arm individually randomized superiority trial to estimate the efficacy of the preemptive amoxicillin clavulanate administration compared to no intervention for preventing secondary infection from *Bothrops* snakebites. Clinical trial was performed at the *Fundação de Medicina Tropical Doutor Heitor Vieira Dourado* (FMT-HVD), in Manaus, Western Brazilian Amazon, from August 2014 to September 2016. This tertiary hospital is the reference in the Amazonas state for snakebites treatment. In Manaus, FMT-HVD is the only hospital unit that performs the distribution and administration of snakebite antivenom. At admission, *Bothrops* snakebites were diagnosed with basis in clinico-epidemiological characteristics of the patient and, when the patient brought the snake responsible by the envenomation, by its identification made by a trained biologist.

Sample size calculation was based on the mean of 240 snakebites/year attended at FMT-HVD, with an expected frequency of secondary infection of 40% [13], a 50% risk reduction of infection, at an 80% power and 5% of significance level and an 1:1 randomization ratio. Adding 10% of losses in the follow-up, a sample size of 186 participants was obtained, with 93 patients in the intervention group and 93 in the untreated group.

### Eligibility, randomization and intervention

Patient was eligible if admitted to the hospital with less than 24 hours after the bite, without antivenom therapy in other hospital and without any sign of secondary infection at this time. Patients that used any antibiotic in the past 30 days, pregnant women or patients with previous history of allergic reactions to antibiotics were not included in this trial.

After application of eligibility criteria, the study pharmacist was contacted to obtain the allocation group to the patient. Randomization sequences with an allocation ratio of 1:1 were computer-generated by a random table, to the intervention group (preemptive amoxicillin clavulanate) or to the control group (no preemptive antibiotic prescription). All laboratory staff was blinded for treatment assignment. The antibiotic chosen for this trial was oral tablet amoxicillin clavulanate 875/125 mg to adults and 25 mg/kg/day to children twice per day for seven days, starting at the admission day.

### Admission and follow-up procedures

After patient inclusion, demographic and epidemiological information was collected using a standardized questionnaire, including gender, age (in years), area of occurrence (urban or rural), anatomical site of the bite, work-related bite (yes or no), time elapsed from bite to medical assistance (in hours), walking after bite (in minutes), previous history of snakebite and pre-admission conduits (use of topical or oral medicines, use of tourniquet and other procedures). A detailed clinical and laboratorial characterization was also made at this time. Pain assessment was made using the Numerical Rating Scale, with values rating from 1 to 10 [44]; pain was further classified as absent (rate 0), mild (rated from 1 to 3), moderate (rated from 4 to 7) and severe (rated from 8 to 10). Edema was classified as absent, mild (affecting 1–2 limb segments), moderate (affecting 3–4 limb segments) and severe (affecting more than 5 limb segments) [11]. Bite site temperature (^o^C) was measured using an infrared digital thermometer (Color Check AC322); the difference between the bite site temperature and the contralateral limb site was calculated. Presence of local bleeding, lymphadenitis and necrosis was also assessed. Systemic signs and symptoms, such as systemic bleeding, signs of acute renal failure, headache, dizziness and vomiting were recorded from patients. Vital signs (blood pressure, heart rate, respiratory rate and axillary temperature) were also assessed. All the clinical information was collected through a standardized clinical registration form. Immediately after clinical examination, a 15 mL blood sample was taken for laboratorial analysis. Tests included leukocyte count (cells/μL), fibrinogen (mg/dL), platelet count (number/μL), hemoglobin (mg/dL), creatine phosphokinase (IU/L), creatine phosphokinase-MB (ng/mL), erythrocyte sedimentation rate (mm/hour), lactate dehydrogenase (IU/L), creatinine (mg/dL), urea (mg/dL), aspartate transaminase (IU/L), alanine transaminase (IU/L), clotting time (in minutes), prothrombin time (in seconds) and C-reactive protein (mg/dL). An aliquot was submitted to an enzyme immunoassay to confirm *Bothrops* envenoming diagnosis and to determine circulating venom levels in all patients [45]. All the laboratory results were transferred to a standardized registration form.

According to clinical severity, patients were classified using to the Brazilian Health Ministry guidelines [7]: i) mild cases: local pain, local swelling and bruising for *Bothrops*; ii) moderate cases: local manifestations without necrosis and minor systemic signs (coagulopathy and bleeding, no shock); iii) severe cases: life- threatening snakebite, with severe bleeding, hypotension/shock and/or acute renal failure.

Both groups were submitted to the same local wound care with daily 0.9% saline cleaning. Thirty minutes before antivenom therapy, the intervention group of patients took amoxicillin clavulanate, supervised by a nurse. Twenty minutes after pre-medication with IV hydrocortisone (500 mg), IV cimetidine (300 mg) and oral dexchlorpheniramine (5 mg), antivenom therapy was given to all patients from both arms in a dosage corresponding to the severity grading, according the Brazilian official guidelines [7].

Intervention and control groups were hospitalized for 3 days and returned to the hospital 7 days after admission. A full clinical and laboratory examination were performed 24 hours, 48 hours, 72 hours and 7 days after admission.

### Endpoints

The primary efficacy endpoint of this trial was the time free of secondary infection at snakebite site, defined as the presence of cellulitis and/or abscess [38,39], until 7 days after hospital admission. The onset of secondary infection until 48 hours at admission was considered a secondary outcome. Cellulitis was defined by the presence of local inflammation signs (erythema, edema, bruising and pain) with association to fever, leukocytosis, lymphangitis and/or lymphadenitis [46]. An abscess was characterized by individual injuries, floating, presenting purulent secretion or serous-purulent secretion [46,47]. Two independent observers evaluated all the patients and came to a final agreement. Patients clinically diagnosed with secondary infection were additionally evaluated by ultrasonography and a sample collection for microbiology was obtained only in cases evolving to abscess.

After secondary infection diagnosis, amoxicillin clavulanate was interrupted and patient was treated accordingly medical discretion.

### Statistical analysis

Before statistical analysis, two independent typists entered information using Epi Info 3.5.1. A study researcher solved disagreements. The primary efficacy analysis was done on all randomized participants finishing the follow-up (per protocol population). The primary efficacy endpoint, secondary infection-free at 7 days, was analyzed using Kaplan-Meier estimates. A two-sided log-rank test was done over the period using a 5% significance level. Patients that were secondary infection-free at day 7 were censored at this point. The effect of drug use (amoxicillin clavulanate) was assessed as a single block. Relative risk, relative risk reduction, absolute risk reduction and number needed to treat were assessed for primary and secondary outcomes. For the secondary infection risk analysis, at 48 hours and 7 days of follow-up, explanatory variables were grouped in hierarchical blocks [48]. Proximal block was composed by laboratory findings at admission, intermediate block by clinical findings at admission and distal block by demographic and epidemiological variables. Univariate regression analysis was carried out for each block individually. Variables with a significance level of p<0.2 were included in the multivariate analysis by block. All variables with a significance level of p<0.05 in the multivariate analysis by block were thus included in the overall model (all blocks together). Crude odds ratios (OR), adjusted odds ratios (AOR) with their respective confidence intervals were calculated for each hierarchical level and for the overall model. Accuracy of the final model was evaluated by Hosmer-Lemeshow goodness-of-fit test. Where zeros caused problems with computation of the OR and 95% CI, 0.5 was added to all cells [49,50]. Mann-Whitney tests were carried out to assess differences between median of treated and untreated groups. Statistical analyses were performed using the STATA statistical package version 13 (Stata Corp. 2013).

## Results

### Patients’ characterization

**Fig 1:**
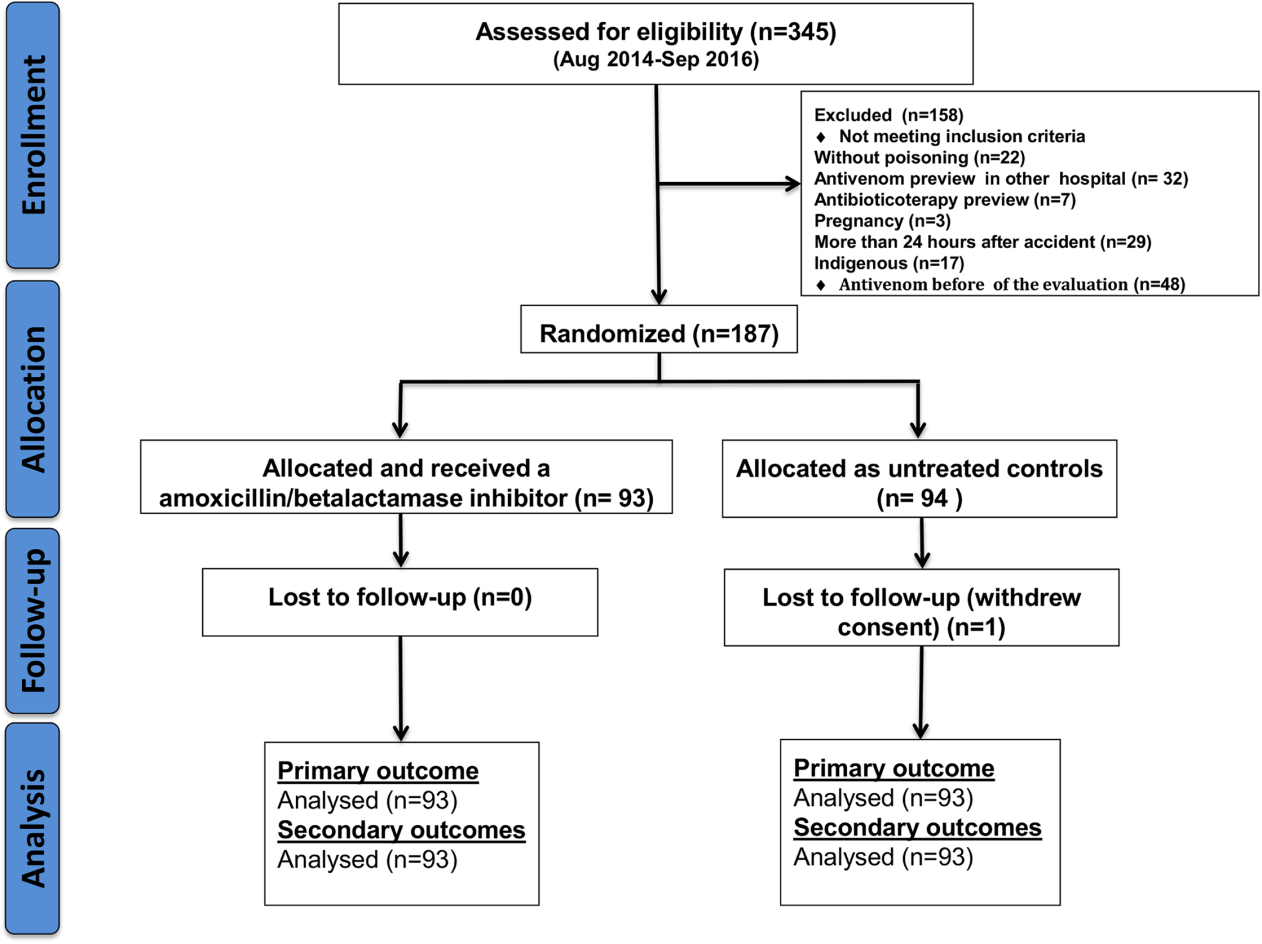
Flow chart of inclusion. Recruitment of the patients attended by snakebite at FMT-HVD, allocation and follow-up in the clinical trial.

A total of 345 patients were assessed for eligibility in the study period. From this total, 187 accomplished the inclusion criteria and were randomized, with 93 in the interventional group and 94 in the untreated control group. One patient of the control group was lost to follow up (Fig 1). Enzyme immunoassay confirmed *Bothrops* envenoming diagnosis in all included patients.

Epidemiological characterization showed predominance of males (82.3%), mostly occurring in rural areas (87.1%). The most affected age group was the 21–30 years old (22.6%). The most affected anatomical site was the foot (66.1%). A total of 40.3% of cases were classified as work-related bites and 65.6% of the patients walked after the snakebite. Time elapsed from bite to medical assistance was higher than 3 hours in 42.4% of the cases. Use of topical medicines was informed in 34.4%, oral medicines in 28.5% and tourniquet in 24.7% of the cases (Table 1).

**Table 1:** Demographic end epidemiological characteristics of the randomized patients according to the experimental group.

Characteristics	Intervention group (n = 93)		Control group (n = 93)		Total (n = 186)	
	Number	%	Number	%	Number	%
Gender						
Male	78	83.9	75	80.6	153	82.3
Female	15	16.1	18	19.4	33	17.7
Age group (in years)						
0–10	8	8.6	5	5.4	13	7.0
11–20	13	14.0	21	22.6	34	18.3
21–30	22	23.7	20	21.5	42	22.6
31–40	11	11.8	16	17.2	27	14.5
41–50	13	14.0	10	10.8	23	12.4
51–60	14	15.0	14	15.1	28	15.1
>60	12	12.9	7	7.5	19	10.2
Area of occurrence						
Rural	80	86.0	82	88.2	162	87.1
Urban	13	14.0	11	11.8	24	12.9
Anatomical site of the bite						
Upper limbs	1	1.1	1	1.1	2	1.1
Lower limbs	18	19.4	16	17.2	34	18.3
Hand	12	12.9	15	16.1	27	14.5
Foot	62	66.7	61	65.6	123	66.1
Work-related bite						
Yes	32	34.4	43	46.2	75	40.3
No	61	65.6	50	53.8	111	59.7
Walking after bite (in minutes)						
No	33	35.5	31	33.3	64	34.4
5–9	17	18.3	18	19.4	35	18.8
10–29	24	25.8	24	25.8	48	25.8
30–59	12	12.9	15	16.1	27	14.5
>60	7	7.6	5	5.4	12	6.5
Time elapsed from bite to medical assistance (hours)						
0–3	53	57.0	54	58.1	107	57.6
4–6	22	23.7	27	29.0	49	26.3
7–12	8	8.6	4	4.3	12	6.5
13–24	10	10.8	8	8.6	18	9.7
Previous snakebite history						
Yes	14	15.1	12	12.9	160	86.0
No	79	84.9	81	87.1	26	14.0
Use of topical medicines						
Yes	32	34.4	32	34.4	64	34.4
No	61	65.6	61	65.6	122	65.6
Use of oral medicines						
Yes	19	20.4	34	36.6	53	28.5
No	74	79.6	59	63.4	133	71.5
Use of tourniquet						
Yes	25	26.9	21	22.6	46	24.7
No	68	73.1	72	77.4	140	75.3

The most frequent manifestations observed at admission were severe pain (46.2%), mild edema (48.4%) and local bleeding (46.8%). The difference of temperature between the bite site and the contralateral site was predominantly <1^°^C (51.9%). The most frequent systemic manifestations were headache (26.9%), dizziness (14.5%), gingival bleeding (8.6%), nausea (8.1%) and vomiting (7.0%). Mean axillary temperature was 36.1 ^o^C, mean heart rate was 79.3 bpm, mean respiratory rate was 20 bpm, mean systolic pressure (mmHg) was 129.8 mmHg and mean diastolic pressure (mmHg) was 82.6 mmHg. Snakebites were classified as moderate in 48.9% of the cases (Table 2). No compartmental syndrome, sepsis, gangrene, amputation or death was seen.

**Table 2:** Clinical characterization of the randomized participants according to the experimental group.

Characteristics	Intervention group (n = 93)		Control group (n = 93)		Total (n = 186)	
	Number	%	Number	%	Number	%
**Local signs**						
**Pain**						
Absent	12	12.9	14	15.1	26	14.0
Mild	13	14.0	11	11.8	24	12.9
Moderate	24	25.8	26	28.0	50	26.9
Severe	44	47.3	42	45.2	86	46.2
**Edema**						
Mild	50	53.8	40	43.0	90	48.4
Moderate	38	40.9	44	47.3	82	44.1
Severe	5	5.4	9	9.7	14	7.5
**Local bleeding**						
Yes	35	37.6	52	55.9	87	46.8
No						
**Difference between bite site and the contralateral site temperature (**^**o**^**C)**						
No difference	6	6.5	3	3.3	9	4.9
0.1–0.9	49	52.7	47	51.1	96	51.9
1–1.9	19	20.4	26	28.3	45	24.3
2–2.9	13	14.0	11	12.0	24	13.0
>3	6	6.5	5	5.4	11	5.9
**Systemic signs and symptoms**						
Headache	26	28.0	24	25.8	50	26.9
Dizziness	12	12.9	15	16.1	27	14.5
Gingival bleeding	9	9.7	7	7.5	16	8.6
Vomiting	8	8.6	5	5.4	13	7.0
Nausea	7	7.5	8	8.6	15	8.1
**Vital signs**						
Axillary temperature (^**o**^ C)	36.0	1.5	36.2	0.6	36.1	1.1
Heart rate (bpm)	84.0	45.7	74.8	14.6	79.3	33.8
Respiratory rate (bpm)	20.0	2.5	20.0	1.9	20.0	2.9
Systolic pressure (mmHg)	130	19.6	129.6	18.2	129.8	18.9
Diastolic pressure (mmHg)	82.4	12.6	82.7	15.4	82.6	14.0
**Snakebite clinical classification**						
Mild	47	50.5	33	35.5	80	43.0
Moderate	41	44.1	50	53.8	91	48.9
Severe	5	5.4	10	10.8	15	8.1

Laboratorial characterization revealed mild leukocytosis, hypofibrinogenemia, increased creatine phosphokinase and creatine phosphokinase-MB activities, increased erythrocyte sedimentation rate and mildly increased lactate dehydrogenase activity. Clotting time presented incoagulable in 57.5% of patients. Prothrombin time presented incoagulable in 40.9% of patients. C-reactive protein was >6.5 mg/dL in 18.8% of the cases. Mean blood venom concentration was 50.9 ng/mL (Table 3). No statistical differences were observed between treated (median = 12) and untreated (median = 6.5) groups when CRP medians were compared (Z = -0.161, p-value = 0.872).

**Table 3:** Laboratorial characterization of the randomized participants according to the experimental group.

Laboratorial test	Intervention group (n = 93)	Control group (n = 93)	Total (n = 186)
	Mean (SD)/n (%)	Mean (SD)/n (%)	Mean (SD)/n (%)
Leukocyte count (cells/μL)	11,600 (8,900)	12,700 (8,700)	12,200 (8,800)
Fibrinogen (mg/dL)	204.0 (124.2)	178.4 (94.2)	174.1 (116.6)
Platelet (number/μL)	234,500 (74,400)	256,300 (71,200)	245,400 (73,400)
Hemoglobin (mg/dL)	14.4 (1.3)	14.4 (1.5)	14.4 (1.4)
Creatine phosphokinase (IU/L)	210.8 (179.4)	260.2 (289.6)	235.5 (241.5)
Creatine phosphokinase-MB (IU/L)	28.6 (33.2)	27.4 (22.1)	28.0 (28.1)
Erythrocyte sedimentation rate (mm/hour)	15.6 (17.6)	15.9 (17.8)	15.8 (17.7)
Lactate dehydrogenase (IU/L)	475.2 (598.0)	435.9 (258.2)	455.6 (459.8)
Creatinine (mg/dL)	1.0 (0.3)	1.0 (0.5)	0.9 (0.4)
Urea (mg/dL)	32.7 (11.2)	33.3 (13.6)	33.0 (12.4)
Aspartate transaminase (UI/L)	29.2 (13.6)	35.0 (25.1)	32.1 (20.3)
Alanine transaminase (UI/L)	24.2 (19.4)	27.2 (36.9)	25.7 (29.5)
Clotting time (minutes)			
Normal (4–10)	30 (32.3)	33 (35.5)	63 (33.9)
Prolonged (>10)	7 (7.5)	9 (9.7)	16 (8.6)
Incoagulable	56 (60.2)	51 (54.8)	107 (57.5)
Prothrombin time (seconds)			
Normal (10–14)	4 (4.3)	8 (8.6)	12 (6.5)
Prolonged (>14)	51 (54.8)	47 (50.5)	98 (52.6)
Incoagulable	38 (40.9)	38 (40.9)	76 (40.9)
C-reactive protein (mg/dL)			
<6.5	76 (81.7)	75 (80.7)	151 (81.9)
>6.5	17 (18.3)	18 (19.4)	35 (18.8)
Blood venom concentration (ng/ml)	52.3 (42.0)	49.0 (59.6)	50.9 (50.0)

**Reference values:** Leukocytes: 4.000–10.000/mm^3^; Fibrinogen: 200–400 mg/dL; Platelets: 130,000–400.000/mm^3^; Hemoglobin: 13.0–16.0 g/dL for males and 12.0–14.0 for females; Creatine phosphokinase: 24–190 IU/L; Creatine phosphokinase-MB: 2–25 IU/L; Erythrocyte sedimentation rate: <10 mm/hour; Lactate dehydrogenase: 211–423 IU/L; Creatinine: 0.5–1.2 mg/dL for adults and 0.3–1.0 mg/dL for children; Urea: 10–45 mg/dL; Aspartate transaminase: 2–38 IU/L; Alanine transaminase: 2–44 UI/L; Clotting time: 4–10 minutes; Prothrombin time: 10–14 seconds; C-reactive protein: <6.5 mg/dL.

### Outcome rates and efficacy estimates

**Fig 2:**
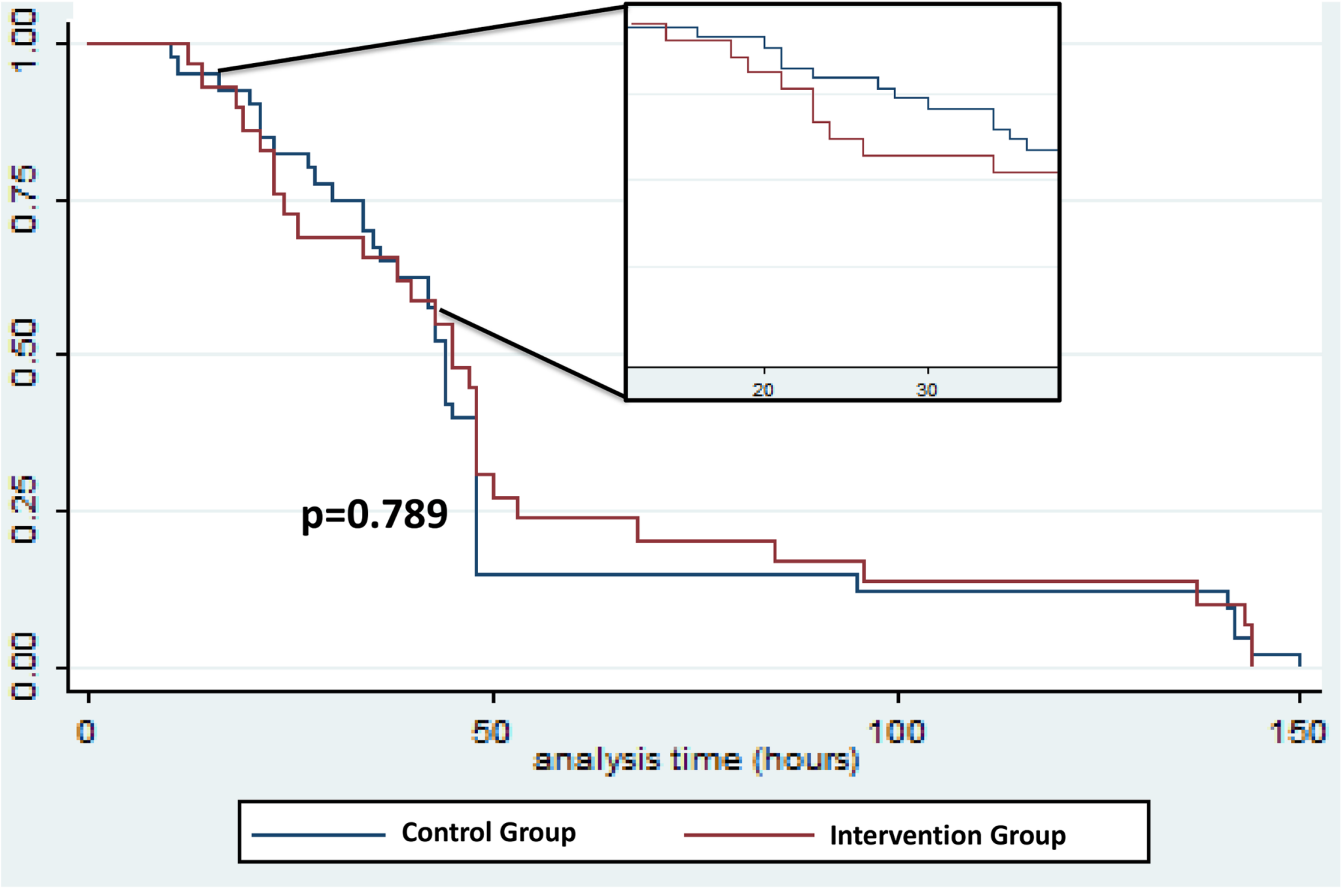
Time free of secondary infection at snakebite site, until 7 days after hospital admission, for both groups. Survival analysis demonstrating that the time from patient admission to the onset of secondary infection was not different between amoxicillin clavulanate treated and control group (Log-rank = 2.23; p = 0.789).

Of the 74 patients with secondary infection, cellulitis was diagnosed in 64 and abscess in 29. Secondary infection rates observed in the intervention and control groups are shown in Table 4. Survival analysis demonstrated that the time from patient admission to the onset of secondary infection was not different between amoxicillin clavulanate treated and control group (Log-rank = 2.23; p = 0.789) (Fig 2). Secondary infection incidence until 7 days after admission was 35.5% in the intervention group and 44.1% in the control group [RR = 0.80 (95%CI = 0.56 to 1.15; p = 0.235)]. Cellulitis rate was 30.1% in the intervention group and 38.7% in the control group [RR = 0.78 (95%CI = 0.52 to 1.16; p = 0.279)]. The abscess rate was 15.1% in the intervention group and 16.1% in the control group [RR = 0.93 (95%CI = 0.48 to 1.82; p = 0.999)].

**Table 4:** Secondary infection rates of the patients followed up in the clinical trial, according to the experimental group.

Outcome	Total (n, %)	Intervention group (n, %)	Control group (n, %)	Relative risk
Secondary infection in 7 days	74 (39.8)	33 (35.5)	41 (44.1)	0.80 (0.56 to 1.15)
Cellulitis in 7 days	64 (34.4)	28 (30.1)	36 (38.7)	0.78 (0.52 to 1.16)
Abscess in 7 days	29 (31.9)	14 (15.1)	15 (16.1)	0.93 (0.48 to 1.82)
Secondary infection in 48 hours	55 (29.6)	21 (22.6)	34 (36.6)	0.62 (0.38 to 0.98)

Secondary infection incidence until 48 hours after admission was 22.6% in the intervention group and 36.6% in the control group [RR = 0.62 (95%CI = 0.38 to 0.98; p = 0.038)]. Actually, survival analysis has shown a later onset of secondary infection in the treated group (Fig 2). No late secondary infection (after 7 days of follow-up) was observed.

From the total of 74 patients presenting secondary infections, 88.2% were males and 88.2% occurred in the rural area. The age groups more affected by secondary infections after snakebite were 31–40 and 51–60 years old, with 20.6% each. Secondary infections were recorded mostly from bites in the foot (61.8%). Infections were secondary to work-related snakebites in 41.2%. Time to medical assistance was less than 3 hours after snakebite in 61.8% of the secondary infections cases. Secondary infections were mostly observed in moderate snakebites (58.8%). Use of local products was made in 35.5% of the secondary infections cases and of tourniquets in 26.5%.

Samples from secondary infection injuries were collected for culture from 11 patients, with 6 positive cases. Microorganisms isolated were *Morganella morganii* (five cases) and *Staphylococcus aureus* (one case).

### Factors associated to secondary infections

Considering proximal variables, secondary infections incidence in 7 days of follow-up was significantly associated to fibrinogen >400 mg/dL [AOR = 3.39 (95%CI = 1.72 to 6.66; p<0.001)], alanine transaminase >44 IU/L [AOR = 2.21 (95%CI = 1.03 to 4.75; p = 0.006)] and C-reactive protein >6.5 mg/L [AOR = 3.90 (95%CI = 1.98 to 7.69; p = <0.001)]. Regarding intermediate variables, moderate [AOR = 11.75 (95%CI = 2.47 to 55.86; p = 0.002)] and severe pain [AOR = 16.97 (95%CI = 2.05 to 340.80; p = 0.001)], moderate [AOR = 3.46 (95%CI = 1.63 to 7.35; p = 0.001)] and severe edema [AOR = 3.78 (95%CI = 1.20 to 11.90; p = 0.023)] and moderate [AOR = 2.52 (95%CI = 1.32 to 4.82; p = 0.005)] and severe snakebites [AOR = 2.80 (95%CI = 0.90 to 8.77; p = 0.076)]. No distal variable was associated to secondary infections incidence (Table 5).

**Table 5:** Factors associated to secondary infection until 7 days of the snakebite patients attended in the hospital in Manaus, 2014 to 2016.

Variables	Secondary infection Number (%)	Without secondary infection Number (%)	Crude OR (IC95%)	p	AOR (IC95%)	p
**Proximal variables**						
**Leukocyte counts**						
>10.000/mm^3^	20 (27.0)	18 (16.1)	1.93 (0.94 to 3.97)	0.072	1.70 (0.73 to4.00)	0.222
**Fibrinogen**						
>400 mg/dL	49 (66.2)	43 (38.4)	3.15 (1.70 to 3.81)	0.000	**3.39 (1.72 to6.66)**	<**0.001**
**Platelet counts**						
<130,000/mm^3^	3 (4.1)	8 (7.2)	0.55 (0.14 to 2.14)	0.388	…	…
**Hemoglobin**						
Lower than normal	1 (1.3)	12 (10.7)	0.11 (0.01 to 0.90)	0.039	0.18 (0.02 to1.45)	0.108
**Creatine phosphokinase**						
>190 IU/L	17 (23.0)	15 (13.4)	1.93 (0.90 to 4.16)	0.093	1.61 (0.65 to3.97)	0.304
**Creatine phosphokinase-MB**						
>25 IU/L	15 (20.3)	29 (25.9)	0.73 (0.36 to 1.48)	0.378	…	…
**Erythrocyte sedimentation rate**						
>10 mm/hour	64 (86.5)	89 (79.5)	1.65 (0.74 to 3.71)	0.223	…	…
**Lactate dehydrogenase**						
>423 IU/L	13 (17.6)	24 (21.4)	0.78 (0.37 to 1.65)	0.519	…	…
**Creatinine**						
Higher than normal	13 (17.6)	14 (12.5)	1.49 (0.66 to 3.39)	0.339	…	…
**Urea**						
>45 mg/dL	12 (16.2)	16 (14.3)	1.16 (0.51 to 2.62)	0.719	…	…
**Aspartate transaminase**						
>38 IU/L	18 (19.3)	17 (15.2)	1.80 (0.86 to 3.77)	0.121	0.86 (0.29 to2.52)	0.783
**Alanine transaminase**						
>44 IU/L	27 (36.5)	24 (21.4)	2.11 (1.10 to 4.06)	0.026	**2.21 (1.03 to4.75)**	**0.042**
**Clotting time**						
>14 minutes	3 (4.0)	5 (5.4)	0.67 (0.04 to 10.88)	0.778	…	…
**Prothrombin time**						
>14 seconds	49 (66.2)	79 (74.1)	1.39 (0.74 to 2.64)	0.321	…	…
**C-reactive protein**						
>6.5 mg/dL	52 (70.3)	42 (37.5)	3.94 (2.10 to 7.38)	<0.001	**3.90 (1.98 to7.69)**	<**0.001**
**Blood venom level**						
>50 ng/mL	57 (77.0)	96 (85.7)	0.56 (0.26 to 1.19)	0.132	0.55 (0.23 to1.35)	0.193
**Intermediate variables**						
**Pain**						
No pain	37 (50.0)	95 (84.8)	1	…	1	…
Mild	12 (16.2)	15 (13.4)	2.05 (0.88 to 4.80)	0.096	1.63 (0.66 to3.98)	0.287
Moderate	18 (24.3)	2 (1.8)	23.11 (5.11 to 104.55)	<0.001	**11.75 (2.47 to55.86)**	**0.002**
Severe	7 (9.5)	0 (0.0)	38.2 (2.13 to 685.5)	<0.001	**16.97 (2.05 to340.80)**	**0.001**
**Edema**						
Mild	21 (28.4)	80 (71.5)	1	…	1	…
Moderate	37 (50.0)	25 (22.3)	5.64 (2.80 to 5.64)	<0.001	**3.46 (1.63 to7.35)**	**0.001**
Severe	16 (21.6)	7 (6.2)	8.71 (3.17 to 23.90)	<0.001	**3.78 (1.20 to11.90)**	**0.023**
**Local bleeding**						
Present	5 (6.8)	2 (1.8)	0.25 (0.47 to 1.33)	0.140	0.28 (0.04 to1.81)	0.182
**Difference between bite site and the contralateral site temperature (⁰C)**						
No difference	4 (5.4)	5 (4.5)	1	…	…	…
0.1–0.9	37(50.0)	59 (53.2)	0.78 (0.20 to 3.11)	0.729	…	…
1–1.9	22 (29.7)	23 (20.7)	1.20 (0.28 to 5.24)	0.808	…	…
2–2.9	7 (9.5)	17 (15.3)	0.51 (0.11 to 2.50)	0.411	…	…
>3	4 (5.4)	7 (6.3)	0.71 (0.12 to 4.32)	0.714	…	…
**Classification of the bite**					
Mild	22 (29.7)	58 (51.8)	1	…	1	…
Moderate	44 (59.5)	47 (42.0)	2.46 (1.30 to 4.68)	0.006	**2.52 (1.32 to4.82)**	**0.005**
Severe	8 (10.8)	7 (6.2)	3.01(0.98 to 9.30)	0.055	2.80 (0.90 to8.77)	0.076
**Distal variables**					
**Gender**						
Male	63 (85.1)	90 (80.4)	0.71 (0.32 to 1.58)	0.405	…	…
**Area of occurrence**						
Rural	65 (87.8)	97 (86.6)	1.03 (0.55 to 1.89)	0.933	…	…
**Age group in years**						
0–10	5 (6.8)	8 (7.2)	1	…	…	…
11–20	10 (13.5)	24 (21.4)	0.67 (0.17 to 2.54)	0.553	…	…
21–30	14 (18.9)	28 (25.0)	0.80 (0.22 to 2.90)	0.734	…	…
31–60	37 (50.0)	41 (36.6)	0.69 (0.19 to 2.34)	0.569	…	…
>60	8 (10.8)	11 (9.8)	1.63 (0.28 to 4.92)	0.837	…	…
**Bite site**					
Upper limbs	-	2 (1.8)	1	…	…	…
Lower limbs	64 (86.5)	92 (82.2)	…	0.991	…	…
Hand	9 (12.5)	18 (16.0)	…	0.991	…	…
**Work-related bite**					
Yes	46 (62.2)	65 (58.0)	0.84 (0.46 to 1.54)	0.575	…	…
**Walking after bite**						
No	24 (32.5)	40 (35.7)	1	…	…	…
5–9 minutes	10 (13.5)	25 (22.3)	0.67 (0.27 to 1.63)	0.372	…	…
10–29 minutes	22 (29.7)	26 (23.2)	1.41 (0.66 to 3.02)	0.376	…	…
30–59 minutes	12 (16.2)	15 (13.4)	1.33 (0.54 to 3.32)	0.537	…	…
>60 minutes	6 (8.1)	6 (5.4)	1.67 (0.48 to 5.76)	0.419	…	…
**Time elapsed from bite to medical assistance **					
0–3 hours	40 (54.1)	67 (59.8)	1	…	…	…
4–6 hours	22 (29.7)	27 (24.1)	1.36 (0.69 to 2.71)	0.374	…	…
7–12 hours	6 (8.1)	6 (5.4)	1.68 (0.51 to 5.55)	0.399	…	…
13–24 hours	6 (8.1)	12 (10.7)	0.84 (0.29 to 2.41)	0.742	…	…
**Previous history of snakebite **					
Yes	14 (18.9)	12 (10.7)	1.94 (0.84 to 4.48)	0.118	1.98(0.84 to4.70)	0.121
**Use of topical medicines**						
Yes	23 (31.1)	40 (35.7)	0.83 (0.44 to 1.55)	0.555	…	…
**Use of oral medicines**						
Yes	23 (31.1)	30 (26.8)	1.23 (0.65 to 2.35)	0.526	…	…
**Use of tournique**t						
Yes	21 (28.4)	25 (22.4)	0.73 (0.37 to 1.42)	0.350	…	…

**Reference values:** Leukocytes: 4,000–10,000/mm^3^; Fibrinogen: 200–400 mg/dL; Platelets: 130,000–400,000/mm^3^; Hemoglobin: 13.0–16.0 g/dL for males and 12.0–14.0 for females; Creatine phosphokinase: 24–190 IU/L; Creatine phosphokinase-MB: 2–25 IU/L; Erythrocyte sedimentation rate: <10 mm/hour; Lactate dehydrogenase: 211–423 IU/L; Creatinine: 0.5–1.2 mg/dL for adults and 0.3–1.0 mg/dL for children; Urea: 10–45 mg/dL; Aspartate transaminase: 2–38 IU/L; Alanine transaminase: 2–44 UI/L; Clotting time: 4–10 minutes; Prothrombin time: 10–14 seconds; C-reactive protein: <6.5 mg/dL.

In the final multivariate analysis model, secondary infections incidence in 7 days of follow-up remained significantly associated to fibrinogen >400 mg/dL [AOR = 4.78 (95%CI = 2.17 to10.55; p<0.001)], alanine transaminase >44 IU/L [AOR = 2.52 (95%CI = 1.06 to 5.98; p = 0.037)], C-reactive protein >6.5 mg/L [AOR = 2.98 (95%CI = 1.40 to 6.35; p = 0.005)], moderate pain [AOR = 24.30 (95%CI = 4.69 to 125.84; p<0.001)] and moderate snakebites [AOR = 2.43 (95%CI = 1.07 to 5.50; p = 0.034)] (Table 6).

**Table 6:** Final model of factors associated to secondary infection until 7 days of the snakebite patients attended in the hospital in Manaus.

Variables	Secondary infection Number, (%)	Without secondary infection Number, (%)	AOR (IC95%)	p
**Fibrinogen**				
<200 mg/dL	49 (66.2)	43 (38.4)	**4.78 (2.17 to10.55)**	**<0.001**
**Alanine transaminase**				
>44 IU/L	27 (36.5)	24 (21.4)	**2.52 (1.06 to 5.98)**	**0.037**
**C-reactive protein**				
>6.5 mg/dL	52 (70.3)	42 (37.5)	**2.98 (1.40 to 6.35)**	**0.005**
**Pain**				
No pain	37 (50.0)	95 (84.8)	1	…
Mild	12 (16.2)	15 (13.4)	2.62 (0.99 to 6.88)	0.051
Moderate	18 (24.3)	2 (1.8)	**24.30(4.69 to 125.84)**	**<0.001**
Severe	7 (9.5)	-	…	…
**Edema**				
Mild	21 (28.4)	80 (71.5)	1	…
Moderate	37 (50.0)	25 (22.3)	1.84 (0.68 to 5.00)	0.229
Severe	16 (21.6)	7 (6.2)	2.03 (0.45 to 9.10)	0.356
**Classification of the bite**				
Mild	22 (29.7)	58 (51.8)	1	…
Moderate	44 (59.5)	47 (42.0)	**2.43 (1.07 to 5.50)**	**0.034**
Severe	8 (10.8)	7 (6.2)	3.64 (0.82 to 16.18)	0.089

Secondary infections incidence in 48 hours of follow-up was significantly associated to C-reactive protein >6.5 mg/L [AOR = 4.28 (95%CI = 1.81 to 10.14; p = 0.001)], moderate [AOR = 5.87 (95%CI = 2.06 to 16.74; p = 0.001)] and severe pain [AOR = 17.89 (95%CI = 1.71 to 186.97; p = 0.016)]. Preemptive amoxicillin clavulanate was protective for secondary infections in 48 hours of follow-up [AOR = 0.42 (95%CI = 0.19 to 0.94; p = 0.034)] (S1 File).

## Discussion

### Preemptive amoxicillin clavulanate efficacy interpretation

Antimicrobial schemes for prevention or treatment of secondary infections from snakebites are not based on good evidences from randomized clinical trials [36,37]. Although the Infectious Diseases Society of America (IDSA) guidelines for diagnosis and management of skin and soft-tissue infections indicates amoxicillin clavulanate to reduce complications by prevention of secondary infection from animal bites [38,39], to the best of our knowledge this is the first trial assessing the efficacy of this regimen for snakebites. In this trial, although lower rates of secondary infection was observed in the intervention group such differences between amoxicillin clavulanate treated and control groups over a follow-up of 7 days did not achieve statistical significance. Consistently, analysis of the subgroups of patients who had abscesses or cellulitis presented also not significantly difference in terms of intervention efficacy. A previous study with oral chloramphenicol showed a poor efficacy in preventing secondary infection from *Bothrops* snakebites [41], although this drug was suggested as a good alternative for the treatment of local infections which may complicate bites by this snake genus [26,51,52]. Accordingly, intravenous chloramphenicol plus gentamicin showed no statistical difference between patients treated and untreated groups in *Crotalus* snakebites [42]. In a previous study amoxacillin was also used preemptively without any clinical benefit [53]. In general, these trials were not guided by the investigation of the bacterial agents responsible by the snakebite site infection in that area. Indeed, even for routine treatment purposes, secondary infection diagnosis is mostly based only from clinical features without microbiological confirmation followed of antimicrobial resistance profile.

Oral microbiota of snakes comprises a wide range aerobial and anaerobial microorganisms, including *Enterobacteriaceae* (namelly *Morganella* spp. and *Escherichia coli*), *Streptococcus*, *Aeromonas* spp., *Staphylococcus aureus* and *Clostridium spp*. [20–22]. Few reports of microbiological confirmation of bacteria responsible for snakebites abscesses demonstrated a predominance of aerobics *Enterobacteriaceae*, mainly *Morganella morganii* [20,23,24]. The infection may not be necessarily associated to snake's mouth flora but also to local disorders induced by venom. The significant difference present at 48 hours but not at 7^th^ day could be explained by the contamination between 3^rd^ day and 7^th^ day with origin other than oral microbiota of the snake, such as patient microbiota or iatrogeny. A limitation of this study was the absence of definitive identification of bacteria responsible by the infections for most of the participants. From six bacterial isolations, however, *Morganella morganii* was present in five snakebite site infections, however no antibiogram was routinely performed. Resistance to β-lactam antibiotics in *Morganella* species is very common and usually mediated by the presence of chromosomally encoded β-lactamases belonging to the AmpC β-lactamase family. These β-lactamases are typically inducible in the presence of β-lactam antibiotics [54]. As a result, agents such as ampicillin, amoxicillin, and first-generation and some second-generation cephalosporins may be ineffective [55]. This finding highlights the need of previous knowledge of the secondary infections epidemiology as a cornerstone in the preemptive antibiotics trials in snakebites.

Secondary infection incidence until 48 hours after admission had a relative risk reduction of 38.3% for the group using preemptive amoxicillin clavulanate compared to control, showing that antibiotic regimen delayed the onset of secondary infection among treated patients. As the preventive effect did not extend until the end of the follow-up, this delay may represent a severe risk for patients using ineffective preventive antibiotics regimens, because in the absence of signs of local complications patients are usually discharged after 48 hours of hospitalization, and may develop secondary infection without proper medical attention. In the difficulty for riverine and indigenous populations living in remote areas returning to health centers for treatment of this complication, severe clinical conditions such as functional loss, amputation, sepsis and even deaths are possible [28]. Although common in endemic areas, our results point that routine empirical prophylactic use of antibiotics aiming to prevent secondary infection lacks a clearly defined policy, leading to a costly and wasteful inappropriate antibiotic use, and even a risk for the patient [22,40]. Unfortunately due to the limited funding, a double-blinded study was not performed, a possible limitation of the study. Another limitation was the enrollment of patients with less than 24 hours after the bite, what may have selected those less prone to develop secondary infection.

### Factors associated to secondary bacterial infection from *Bothrops* snakebites

In *Bothrops* snakebites, studies mostly describe factors associated to systemic complications, such as coagulopathy [11,20], acute renal failure [11,56–59] and death [11,60,61], with extreme age groups and time to medical assistance associated with these poor outcomes [61]. There is scarce information in relation to local complications, especially necrosis [59,62] and amputation [63], associated to anatomical region bitten, systemic bleeding, renal failure, older age and use of tourniquet. Although secondary bacterial infections were observed in around 40% of the *B*. *atrox* snakebites in the Amazon [13], as confirmed in this work, no epidemiological or clinical predictive marker is known for this complication.

In this work, secondary infections incidence was significantly associated to higher levels fibrinogen, alanine transaminase and C-reactive protein, suggesting these laboratorial markers as auxiliary tools for the diagnosis of secondary infections allied to clinical signs of cellulitis and abscesses. Fibrinogen is an acute-phase protein and its serum concentration may be elevated in inflammatory and infectious conditions associated with vascular damage [64,65]. Even that *B*. *atrox* metalloproteinases cleave fibrinogen and induces a drop of fibrinogen levels with an increase in fibrin/fibrinogen degradation products (FDP) levels in vivo, with a foremost role in the pathogenesis of coagulopathy and intravascular hemolysis in the acute envenoming [66,67], the intense inflamatory reaction linked to secondary infection persisting after the antivenom therapy may trigger an acute phase reactant response in affected patients. High C-reactive protein paralleling with high fibrinogen in patients with soft-tissue secondary infections [39]. Our study suggests that the proinflammatory profile present in secondary infections from snakebites may be responsible for hepatocellular dysfunction and further elevated alanine transaminases, as previously reported in patients with sepsis and urinary tract infections [68–72]. Cellulitis or abscesses occur mostly in the moderate or severe snakebite cases, as previously reported in southern Brazil [52].

### Conclusions and perspectives

The Infectious Diseases Society of America (IDSA) guidelines for diagnosis and management of skin and soft-tissue infections indicate amoxicillin clavulanate to prevent secondary infections from animal bites [38,39]. However, in this study, patients did not benefit from preemptive amoxicillin clavulanate in preventing secondary infection from *Bothrops* snakebites. As a perspective, antimicrobial selection to be used in future clinical trials should be pursued diligently in comprehensive snakebites infection management. Secondary infections incidence was significantly associated to higher levels fibrinogen, alanine transaminase and C-reactive protein, suggesting these laboratorial markers as auxiliary tools for a more accurate diagnosis of secondary infections allied to clinical evaluation.

## Supporting information

S1 Checklist CONSORT checklist. (DOC)

S1 File CONSORT flow diagram. (DOC)

S1 Text Risk factors for secondary infection in 48 hours of follow-up. (DOCX)

S1 Dataset Study database. (XLSX)

S1 Appendix Original trial protocol. (PDF)

S2 Appendix Ethics approval document. (PDF)
