# Supplementary material for: Poor efficacy of preemptive amoxicillin clavulanate for preventing secondary infection from Bothrops snakebites in the Brazilian Amazon: A randomized controlled clinical trial
Source: PLoS Negl Trop Dis. 2017 Jul 10;11(7):e0005745. doi: 10.1371/journal.pntd.0005745 (PMC5519217; doi:10.1371/journal.pntd.0005745)
Supplement: S1 File — (DOC) [file pntd.0005745.s002.doc]

**
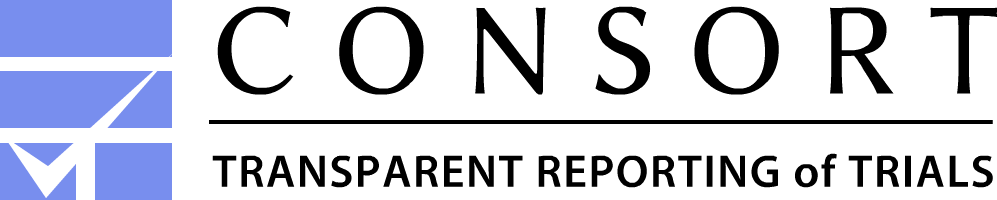
**

**CONSORT 2010 Flow Diagram**

**Allocation**

**Analysis**

**Follow-Up**

**Enrollment**

Assessed for eligibility (n=345)

Excluded (n=158)

♦  Not meeting inclusion criteria (n=110)

♦  Declined to participate (n=0)

♦  Other reasons (n=48)

Analysed (n=93)
♦ Excluded from analysis (give reasons) (n=0)

Lost to follow-up (give reasons) (n=0)

Discontinued intervention (give reasons) (n=0)

Allocated to intervention (Intervention group) (n=93)

♦ Received allocated intervention (n=93)

♦ Did not receive allocated intervention (give reasons) (n=0)

Lost to follow-up (withdrew consent) (n=1)

Discontinued intervention (give reasons) (n=0)

Allocated to intervention (Control Group) (n=94)

♦ Received allocated intervention (n=94)

♦ Did not receive allocated intervention (give reasons) (n=0)

Analysed (n=93)
♦ Excluded from analysis (give reasons) (n=0)

Randomized (n=187)
