# Supplementary material for: Poor efficacy of preemptive amoxicillin clavulanate for preventing secondary infection from Bothrops snakebites in the Brazilian Amazon: A randomized controlled clinical trial
Source: PLoS Negl Trop Dis. 2017 Jul 10;11(7):e0005745. doi: 10.1371/journal.pntd.0005745 (PMC5519217; doi:10.1371/journal.pntd.0005745)
Supplement: S1 Text — (DOCX) [file pntd.0005745.s003.docx]

**S1 Text.** Risk factors for secondary infection in 48 hours of follow-up.

Considering proximal variables, secondary infections incidence in 48 hours of follow-up was significantly associated to fibrinogen >400 mg/dL [AOR=1.96 (95%CI=0.99 to 3.89; p=0.054)], reactive C protein >6.5 mg/L [AOR=3.78 (95%CI=1.85 to 7.72; p<0.001)]. Regarding intermediate variables, moderate [AOR=4.78 (95%CI=1.79 to 12.75; p=0.002)] and severe pain [AOR=9.93 (95%CI=1.05 to 93.77; p=0.045)] and moderate [AOR=5.87 (95%CI=2.40 to14.39; p<0.001)] and severe edema [AOR=6.22 (95%CI=2.04 to18.88; p=0.023)]. No distal variable was associated to secondary infections incidence (Table A).

In the final multivariate analysis model, secondary infections incidence in 48 hours of follow-up was significantly associated to reactive C protein >6.5 mg/L [AOR=4.28 (95%CI=1.81 to 10.14; p=0.001)], moderate [AOR=5.87 (95%CI=2.06 to 16.74; p=0.001)] and severe pain [AOR=17.89 (95%CI=1.71 to 186.97; p=0.016)] and preemptive amoxicillin clavulanate [AOR=0.42 (95%CI=0.19 to 0.94; p=0.034)] (Table B).

**Table A.** Factors associated to secondary infection until 48 hours of the snakebite patients.

| **Variables** | **Secondary infection (Number, %)** | **Without secondary infection (Number, %)** | | **Crude OR (IC95%)** | | **p** | **AOR (IC95%)** | **p** | |
| --- | --- | --- | --- | --- | --- | --- | --- | --- | --- |
| **Proximal variables** | | | | | | | | | |
| **Leukocyte counts** |  |  |  | | |  |  | |  |
| >10.000/mm³ | 18 (32.7) | 38 (29.0) | 1.19 (0.60 to 2.35) | | | 0.614 | … | | … |
| **Fibrinogen** |  |  |  | | |  |  | |  |
| >400 mg/dL | 28 (50.9) | 38 (29.0) | 2.54 (0.33 to 4.86) | | | **0.005** | **1.96 (0.99-3.89)** | | **0.054** |
| **Platelet counts** |  |  |  | | |  |  | |  |
| <130,000/mm³ | 2 (3.6) | 7 (5.3) | 0.67 (0.13 to 3.32) | | | 0.623 | … | | … |
| **Hemoglobin** |  |  |  | | |  |  | |  |
| Lower than normal | 1 (1.8) | 7 (5.3) | 0.33 (0.04 to 2.73) | | | 0.303 | … | | … |
| **Creatine phosphokinase** |  |  |  | | |  |  | |  |
| >190 IU/L | 17 (30.9) | 24 (18.3) | 1.99 (0.97 to 4.11) | | | 0.061 | 1.25 (0.55-2.84) | | 0.599 |
| **Creatine phosphokinase-MB** |  |  |  | | |  |  | |  |
| >25 IU/L | 12 (21.8) | 36 (27.5) | 0.74 (0.35 to 1.55) | | | 0.422 | … | | … |
| **Erythrocyte sedimentation rate** |  |  |  | | |  |  | |  |
| >10 mm/hour | 45 (81.8) | 103 (78.6) | 1.22 (0.55 to 2.73) | | | 0.623 | … | | … |
| **Lactate dehydrogenase** |  |  |  | | |  |  | |  |
| >423 IU/L | 10 (18.2) | 24 (18.3) | 1.09 (0.44 to 2.24) | | | 0.982 | … | | … |
| **Creatinine** |  |  |  | | |  |  | |  |
| Higher than normal | 12 (21.8) | 17 (13.0) | 1.87 (0.83 to 2.24) | | | 0.133 | 1.26 (0.51-3.11) | | 0.616 |
| **Urea** |  |  |  | | |  |  | |  |
| >45 mg/dL | 11 (20.0) | 21 (16.0) | 1.31 (0.58 to 2.94) | | | 0.514 |  | |  |
| **Aspartate transaminase** |  |  |  | | |  |  | |  |
| >38 IU/L | 13 (23.6) | 14 (10.7) | 2.59 (1.12 to 5.95) | | | 0.025 | 2.31 (0.95-5.64) | | 0.066 |
| **Alanine transaminase** |  |  |  | | |  |  | |  |
| >44 IU/L | 19 (34.5) | 27 (20.6) | 2.03 (1.01 to 4.09) | | | 0.047 | 1.35 (0.60-3.08) | | 0.477 |
| **Clotting time** |  |  |  | | |  |  | |  |
| >14 minutes | 2 (3.6) | 10 (7.6) | 0.43 (0.02 to 9.36) | | | 0.590 | … | | *…* |
| **Prothrombin time** |  |  |  | | |  |  | |  |
| >14 seconds | 39 (70.9) | 108 (82.4) | 0.52 (0.24 to 1.17) | | | 0.122 | 0.84 (0.62-1.19) | | 0.423 |
| **Reactive C protein** |  |  |  | | |  |  | |  |
| >6.5 mg/dL | 41 (74.5) | 48 (36.6) | 5.06 (2.51 to 10.23) | | | <0.001 | **3.78 (1.85-7.72)** | | **<0.001** |
| **Blood venom level** |  |  |  | | |  |  | |  |
| >50 ng/mL | 43 (78.2) | 110 (84.0) | 0.69 (0.31 to 1.51) | | | 0.347 | … | | … |
| **Intermediate variables** | | | | | | | | | |
| **Pain** |  |  |  | |  | |  | |  |
| No pain | 23 (41.8) | 99 (75.6) | 1 | | *…* | | 1 | | … |
| Mild | 10 (18.2) | 22 (16.8) | 1.96 (0.82 to 4.69) | | 0.132 | | 0.82 (0.72-4.65) | | 0.208 |
| Moderate | 17 (30.9) | 9 (6.8) | 8.13 (3.22 to 20.54) | | <0.001 | | **4.78 (1.79-12.75)** | | **0.002** |
| Severe | 5 (9.1) | 1 (0.8) | 21.52 (2.40 to 193.16) | | 0.006 | | **9.93 (1.05-93.77)** | | **0.045** |
| **Edema** |  |  |  | |  | |  | |  |
| Mild | 8 (14.5) | 78 (59.5) | 1 | | *…* | | 1 | | *…* |
| Moderate | 32 (58.2) | 39 (29.8) | 8.00 (3.37 to 19.00) | | <0.001 | | **5.87 (2.40-14.39)** | | **<0.001** |
| Severe | 15 (27.3) | 14 (10.7) | 10.45 (3.74 to 29.25) | | <0.001 | | **6.22 (2.04-18.88)** | | **0.001** |
| **Local bleeding** |  |  |  | |  | |  | |  |
| Present | 5 (9.1) | 6 (4.6) | 1.48 (0.14 to 1.64) | | 0.243 | | … | | … |
| **Difference between bite site and the contralateral site temperature (⁰C)** | | |  | |  | |  | |  |
| No difference | 1 (1.8) | 11 (8.9) | 1 | | *…* | | 1 | | *…* |
| 0.1-0.9 | 14 (25.5) | 45 (36.3) | 3.42 (0.41 to 28.89) | | 0.258 | | 2.22 (0.21-2.24) | | 0.511 |
| 1-1.9 | 17 (30.9) | 32 (25.8) | 5.84 (0.70 to 49.17) | | 0.104 | | 3.69 (0.33-39.34) | | 0.293 |
| 2-2.9 | 9 (16.4) | 18 (14.5) | 5.50 (0.61 to 49.54) | | 0.128 | | 3.40 (0.29-39.16) | | 0.327 |
| >3 | 4 (25.4) | 18 (14.5) | 8.56 (0.98 to 74.41) | | 0.052 | | 9.48 (0.83-108.82) | | 0.071 |
| **Classification of the bite** | |  |  | | |  |  | |  |
| Mild | 19 (34.6) | 61 (46.6) | 1 | | | … | 1 | | … |
| Moderate | 31 (56.4) | 60 (45.8) | 1.66 (1.30 to 4.68) | | | 0.141 | 1.49 (0.75-2.96) | | 0.260 |
| Severe | 5 (9.0) | 10 (7.6) | 1.61(0.49 to 5.28) | | | 0.436 | 1.30 (0.38-4.41) | | 0.677 |
| **Distal variables** | |  |  | | |  |  | |  |
| **Gender** |  |  |  | | |  |  | |  |
| Male | 49 (89.1) | 104 (79.4) | 0.47 (0.18 to 1.22) | | | 0.120 | 0.45 (0.17-1.16) | | 0.099 |
| **Area of occurrence** |  |  |  | | |  |  | |  |
| Rural | 47 (85.5) | 115 (87.7) | 1.18 (0.63 to 2.23) | | | 0.606 | … | | … |
| **Age group in years** |  |  |  | | |  |  | |  |
| 0-10 | 3 (5.5) | 10 (7.6) | 1 | | | *…* | … | | … |
| 11-20 | 8 (14.5) | 26 (19.9) | 1.03 (0.23 to 4.66) | | | 0.974 | … | | … |
| 21-30 | 10 (18.2) | 32 (24.4) | 1.04 (0.24 to 4.54) | | | 0.957 | … | | … |
| 31-60 | 18 (50.9) | 50 (38.2) | 1.40 (0.30 to 6.49) | | | 0.665 | … | | … |
| >60 | 6 (10.9) | 13 (9.9) | 1.54 (0.31 to 7.72) | | | 0.601 | … | | … |
| **Bite site** | |  |  | | |  |  | |  |
| Upper limbs | 0 (0.0) | 2 (1.8) | 1 | | | … | … | | … |
| Lower limbs | 64 (86.5) | 92 (82.2) | 0.48 (0.02 to 6.45) | | | 0.979 | … | | … |
| Hand | 9 (12.5) | 18 (16.0) | 1.05 (0.03 to 15.45) | | | 0.999 | … | | … |
| **Work-related bite** | |  |  | | |  |  | |  |
| Yes | 35 (63.6) | 76 (58.0) | 0.79 (0.41 to 1.51) | | | 0.476 | … | | … |
| **Walking after bite** |  |  |  | | |  |  | |  |
| No | 21 (38.2) | 43 (32.8) | 1 | | | … | 1 | | … |
| 5-9 minutes | 6 (10.9) | 29 (22.2) | 0.42 (0.15 to 1.18) | | | 0.100 | 0.39 (0.14-1.12) | | 0.081 |
| 10-29 minutes | 17 (30.9) | 31 (23.7) | 1.12 (0.51 to 2.47) | | | 0.773 | 0.97 (0.43-2.20) | | 0.937 |
| 30-59 minutes | 9 (16.4) | 18 (13.7) | 1.02 (0.39 to 2.66) | | | 0.961 | 0.94 (0.35-2.55) | | 0.907 |
| >60 minutes | 2 (3.6) | 10 (7.6) | 1.41 (0.08 to 2.04) | | | 0.276 | 0.36 (0.07-1.87) | | 0.223 |
| **Time elapsed from bite to medical assistance** | |  |  | | |  |  | |  |
| 0-3 hours | 32 (58.2) | 75 (57.2) | 1 | | | … | … | | … |
| 4-6 hours | 15 (27.3) | 34 (24.0) | 1.03 (0.50 to 2.16) | | | 0.929 | … | | … |
| 7-12 hours | 3 (5.5) | 9 (6.9) | 0.78 (0.20 to 3.08) | | | 0.724 | … | | … |
| 13-24 hours | 5 (9.0) | 13 (9.9) | 0.90 (0.30 to 2.74) | | | 0.855 | … | | … |
| **Previous history of snakebite** | |  |  | | |  |  | |  |
| Yes | 10 (18.2) | 16 (12.2) | 1.60 (0.67 to 3.78) | | | 0.287 | … | | … |
| **Use of topical medicines** |  |  |  | | |  |  | |  |
| Yes | 17 (30.9) | 46 (35.1) | 0.83 (0.42 to 1.62) | | | 0.580 | … | | … |
| **Use of oral medicines** |  |  |  | | |  |  | |  |
| Yes | 18 (32.7) | 35 (26.7) | 1.33 (0.67 to 2.64) | | | 0.408 | … | | … |
| **Use of tournique**t |  |  |  | | |  |  | |  |
| Yes | 16 (29.1) | 30 (22.9) | 0.72 (0.36 to 1.47) | | | 0.373 | … | | … |

**Table B.** Final multivariate analysis model associated to secondary infection until 48 hours of the snakebite patients.

| **Factors** | **With secondary infection**  **(n %)** | **Without secondary infection**  **(n %)** | **AOR (IC95%)** | **p** |
| --- | --- | --- | --- | --- |
| **Reactive C protein** |  |  |  |  |
| >6.5 mg/dL | 41 (74.5) | 48 (36.6) | **4.28 (1.81 to 10.14)** | **0.001** |
| **Pain** |  |  |  |  |
| No pain | 23 (41.8) | 99 (75.6) | 1 | *…* |
| Mild | 10 (18.2) | 22 (16.8) | 1.92 (0.69 to 5.38) | 0.214 |
| Moderate | 17 (30.9) | 9 (6.8) | **5.87 (2.06 to 16.74)** | **0.001** |
| Severe | 5 (9.1) | 1 (0.8) | **17.89 (1.71 to 186.97)** | **0.016** |
| **Edema** |  |  |  |  |
| Mild | 8 (14.5) | 78 (59.5) |  |  |
| Moderate | 32 (58.2) | 39 (29.8) | **3.94 (1.52 to 10.21)** | **0.005** |
| Severe | 15 (27.3) | 14 (10.7) | 2.70 (0.79 to 2.24) | 0.112 |
| **Preemptive amoxicillin clavulanate** | |  |  |  |
| Yes | 21 (38.2) | 72 (55.0) | **0.42 (0.19 to 0.94)** | **0.034** |
| No | 34 (61.8) | 59 (45.0) |  |  |
