# Supplementary material for: Poor efficacy of preemptive amoxicillin clavulanate for preventing secondary infection from Bothrops snakebites in the Brazilian Amazon: A randomized controlled clinical trial
Source: PLoS Negl Trop Dis. 2017 Jul 10;11(7):e0005745. doi: 10.1371/journal.pntd.0005745 (PMC5519217; doi:10.1371/journal.pntd.0005745)
Supplement: S1 Appendix — (PDF) [file pntd.0005745.s005.pdf]

UNIVERSIDADE DO ESTADO DO AMAZONAS
FUNDAÇÃO DE MEDICINA TROPICAL DR. HEITOR VIEIRA DOURADO

ANTIBIOTICOTERAPIA PRECOCE: UM ENSAIO CLÍNICO RANDOMIZADO PARA AVALIAÇÃO DA EFICÁCIA AMOXICILINA/INIBIDOR DA BETALACTAMASE EM INFECÇÃO SECUNDÁRIA DE PACIENTES VÍTIMAS DE ACIDENTES OFÍDICOS NA AMAZÔNIA BRASILEIRA

MANAUS

2014

RESUMO

Os acidentes ofídicos têm sido considerados como o problema de saúde negligenciado mais importante do século XXI, em função do enorme número de pessoas que acomete e pelo virtual desinteresse por parte do setor produtivo no investimento para melhorar as condições de atendimento a essa população, quase sempre moradora de zonas rurais. Apenas um percentual desses acidentados chegam às unidades de saúde para realização de soroterapia específica. A complicação mais frequente são as infecções bacterianas secundárias, que nem sempre acontecem nas primeiras 24 horas de observação dos pacientes após soroterapia. Muitos desses pacientes retornam para casa sem a nova possibilidade de retorno à unidade de saúde, para tratamento das complicações mediatas do acidente. Na literatura, há um grande desconhecimento sobre fatores de risco para a infecção bacteriana secundária, bem como marcadores clínicos e laboratoriais que permitam identificar essa complicação de forma mais precoce. A antibioticoterapia em geral é empírica e o melhor momento de sua introdução não é conhecido. Esse tipo de acidente poderia se beneficiar de antibioticoterapia precoce, considerando a frequência da complicação e o perfil dos pacientes, que residem em locais sem acesso ao sistema de saúde. Portanto, o presente estudo pretende realizar um ensaio clínico randomizado com o objetivo de identificar a superioridade da introdução precoce de Amoxicilina/Inibidor da Betalactamase em pacientes acidentados por *Bothrops atrox* sobre a não-realização do antibiótico. São também objetivos secundários do estudo a caracterização das infecções secundárias e das alterações de coagulação induzidas pelo veneno que possam contribuir para a complicação infecciosa. Trata-se de um projeto de pesquisa que certamente contribuirá para o benefício do paciente, tanto em aspectos sociais, econômicos e de saúde.

Palavras Chaves: Mordeduras de serpentes, infecção dos ferimentos, antibioticoprofilaxia, *Bothrops*, ensaio clínico.

LISTA DE FIGURAS

Figura 1 - Distribuição espacial dos acidentes ofídicos no Estado do Amazonas, de 2007 a 2012. Área de abrangência, com altas taxas de incidência se estende do Nordeste para a região Central do Estado, onde as taxas de incidência são cerca de 150 casos por 100.000 habitantes/ano 03

Figura 2 - Serpentes envolvidas nos acidentes em humanos na Amazônia Brasileira. Imagens das oito principais espécies de serpentes responsáveis por envenenamentos são mostradas (A-H) 04

Figura 3 - Distribuição Geográfica das espécies de serpentes *Bothrops, Crotalus, Lachesis* e *Micrurus* no Brasil... 04

Figura 4 - Complicações locais, resultantes de acidentes botrópicos. 08

Figura 5 – Acidentes ofídicos com infecção secundária A-D............................. 10

Figura 6 – Entrada da FMT-HVD.. 16

Figura 7 – Evolução histórica dos acidentes com serpentes ocorridos na Fundação de Medicina Tropical Doutor Heitor Vieira Dourado, Manaus (1974-2012) 18

Figura 8 – Fita métrica com escala em centímetro 20

Figura 9 – Termômetro Clínico Digital Infravermelho 21

LISTA DE QUADROS

Quadro 1– Efeitos dos venenos ofídicos de acordo com suas atividades fisiopatológicas. 05

Quadro 2 – Número de ampolas de antiveneno indicada para cada tipo de acidente de acordo com a classificação da gravidade do envenenamento 06

Quadro 3 – Posologia para administração da profilaxia do Clavulin BD® 19

Quadro 4 – Descrição dos exames laboratoriais realizados nos pacientes acompanhados durante 7 dias de acompanhamento 21

SUMÁRIO

1 Introdução 01
1.1 Envenenamentos ofídicos
1.1.1 Epidemiologia 01
1.1.2 Espécies envolvidas nos envenenamentos 03
1.1.3 Manifestações Clínicas 05
1.1.4 Complicações 07
1.2 Infecções Secundárias no acidente ofídico
1.2.1 Frequência 09
1.2.2 Micro-organismo envolvidos 11
1.2.3 Profilaxia e tratamento 12
1.3 Justificativa.. 14

2 Objetivos
2.1 Geral 15
2.2 Específicos.. 15

3 Materiais e Métodos
3.1 Modelo do Estudo 16
3.2 Local do Estudo.. 16
3.3 Plano Amostral 18
3.4 Critérios de Elegibilidade.. 18
3.5 Procedimento de Randomização 19
3.6 Intervenção 19
3.7 Seguimento dos Pacientes 20
3.8 Identificação específica e determinação da Venenemia 22
3.9 Definição de Infecção Secundária 23
3.10 Questões Éticas 23
3.11 Plano Analítico 24

4 Orçamento.. 25

5 Cronograma.. 26

6 Participantes do Projeto.. 27

7 Referências 28

APÊNDICES.. 38

ANEXOS... 62

1 INTRODUÇÃO

1.1 Envenenamentos ofídicos

1.1.1 Epidemiologia

O acidente ofídico resulta em uma lesão causada pela mordida de uma serpente, que, muitas vezes desenvolve feridas infligidas pelos dentes do animal e, consequente envenenamento. Embora estes acidentes ocorram em todo o mundo, estes envenenamentos pós evento são considerados uma ameaça importante, sendo atualmente evidenciados como emergentes para o planejamento de estratégias eficazes no âmbito da saúde pública. Isto é especialmente verdadeiro em áreas rurais de países tropicais e subtropicais, onde os acidentes ofídicos são comuns. Contudo, nestes países o acesso aos serviços de saúde para a administração da soroterapia é limitado. A verdadeira magnitude da ameaça à saúde pública representada pelo acidente ofídico nesses países é desconhecida, o que torna difícil para os profissionais atuantes na saúde pública otimizarem a prevenção e tratamento desses eventos em seus respectivos países (1–4).

Em 2013, o Ministério da Saúde registrou 27.181 casos de acidentes neste país (5). A maior incidência foi observada na região Norte (52,6 casos/100.000 habitantes), seguido pelo Centro-Oeste (16,4/100.000). Estes valores, podem ser mais elevados em áreas remotas da (6) Amazônia brasileira e pode serem subestimados devido à uma subnotificação considerável. Nesta região, trabalhadores do sexo masculino que vivem em áreas rurais, principalmente os que exercem a agricultura, caça e silvicultura (7,8) são os grupos mais afetados, sugerindo fortemente um risco ocupacional. Uma pesquisa na comunidade com população indígena e seringueiros revelou que 13% desses indivíduos tinham sido mordido por serpente durante a sua vida (7). A incidência dos acidentes com serpentes correlaciona-se com o período de chuvas mais elevados (9–12), destacando a vulnerabilidade das comunidades amazônicas, principalmente os que vivem em áreas ribeirinhas (Fig. 1).

Apesar de escassos, alguns estudos apontaram a identificação da espécie *Bothrops* como o tipo agente causador mais comum registrado nos acidentes ofídicos da Amazônia brasileira (~ 80%) (13–15). Acidentes com a espécie *Lachesis* predominaram na série de casos de Manaus (46,8%) (11) e Cruzeiro do Sul (51,3%) (10).

No entanto, os autores destacam a possível confusão na identificação da serpente pela população local uma vez que ambos *Bothrops atrox* e *Lachesis muta* receber o mesmo nome popular "surucucu" em determinadas áreas da Amazônia (10).

Na Amazônia brasileira, relativamente poucos acidentes são causados por serpentes corais (*Micrurus* sp.) (16) e por cascavéis (*Crotalus* sp.) (17). O sistema de vigilância mostra uma taxa global de mortalidade variando de 0,4 para 3,9% (7,8,10–12).

Neste sentido, a predominância de acidentes em áreas rurais é particularmente preocupante para algumas regiões da Amazônia, por apresentar sérios problemas de deslocamento para esses pacientes. Algumas áreas não possuem acesso rodoviário e o deslocamento ocorre exclusivamente por rios até a chegada da unidade de saúde mais próxima, o que pode levar horas ou mesmo dias. Assim, as vítimas de envenenamento por serpentes muitas vezes usam medicamentos caseiros tradicionais (7), que contribuem para o surgimento de gravidade, subnotificação dos casos e até mesmo ocorrência de óbitos. Os acidentes ofídios associados a esse fator possuem uma média de tempo até a unidade de saúde muito semelhante nas diversas regiões da Amazônia (15), mas este tempo é muito maior quando comparado a outras regiões do Brasil (18). Em estudo retratando casos de acidentes no Estado do Amazonas (1989-1996), verificou-se que cerca de 29,5% destes tiveram uma demora de atendimento após 6 horas do evento, com uma letalidade estimada em 0,6% (11).

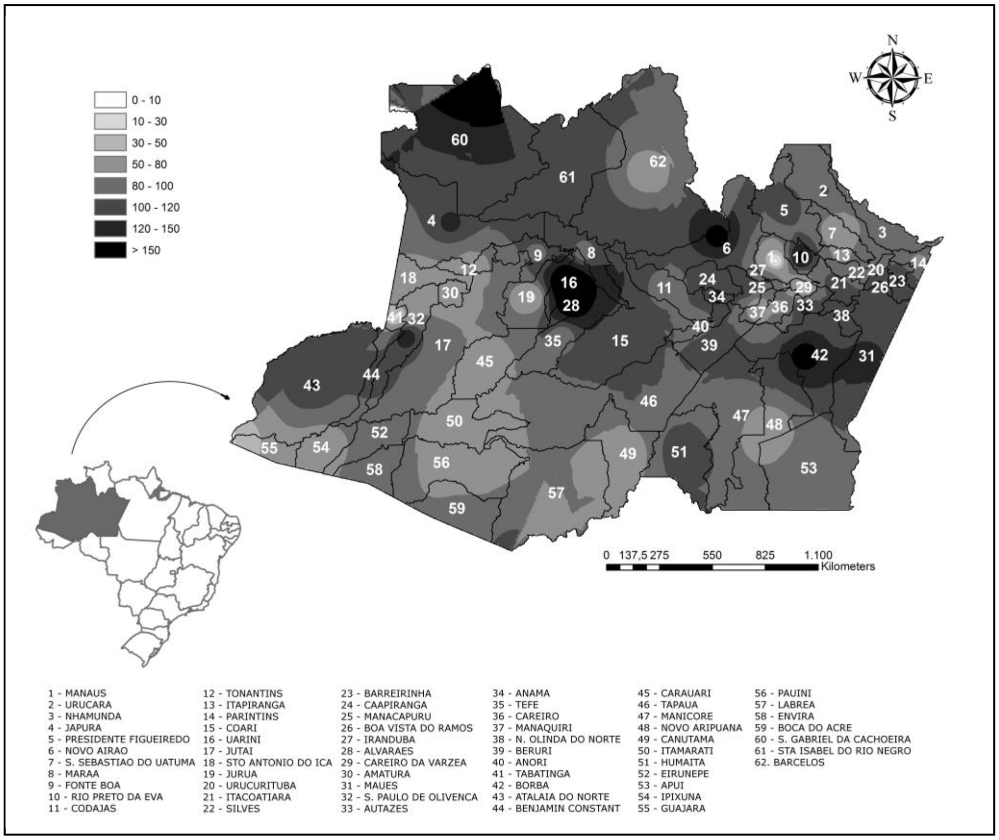

Figura 1 - Distribuição espacial dos acidentes ofídicos no Estado do Amazonas, de 2007 a 2012. Área de abrangência, com altas taxas de incidência se estende do Nordeste para a região Central do Estado, onde as taxas de incidência são cerca de 150 casos por 100.000 habitantes/ano (19).

1.1.2 Espécies envolvidas nos envenenamentos

No Brasil, envenenamentos por serpentes são representados pelos gêneros *Bothrops* (conhecido como, jararaca, jararacuçu, urutu, caiçaca, comboia), *Crotalus* (cascavel), *Lachesis* (surucucu-pico-de-jaca) e *Micrurus* (coral verdadeira) (6). Mordeduras de serpentes não-venenosas também causam lesões, que, muitas vezes devido a lacerações causadas por pelo acidente, podem resultar em infecção secundária (6,20) (Fig. 2).

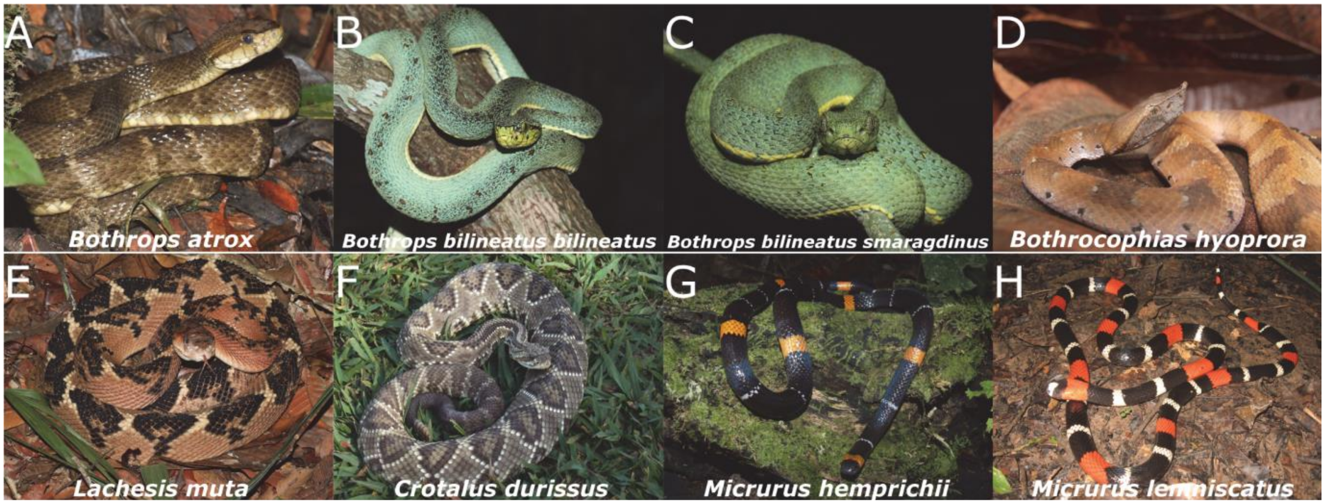

Figura 2 - Serpentes envolvidas nos acidentes em humanos na Amazônia Brasileira. Imagens das oito principais espécies de serpentes responsáveis por envenenamentos são mostradas (A-H): Jararaca-do-norte (A) está implicado na maioria das picadas de cobra humanos registrados na região da Amazônia brasileira (80% -90%), seguido por *Lachesis muta* (E) (21).

A distribuição geográfica das espécies de serpentes foi descrita em 2001 (22) em documentos oficiais, sendo estes, os registros que contempla o cenário brasileiro, entretanto, alguns estudos atualizam as informações do seu habitat de forma pontual (23,24) (10) (Fig. 3).

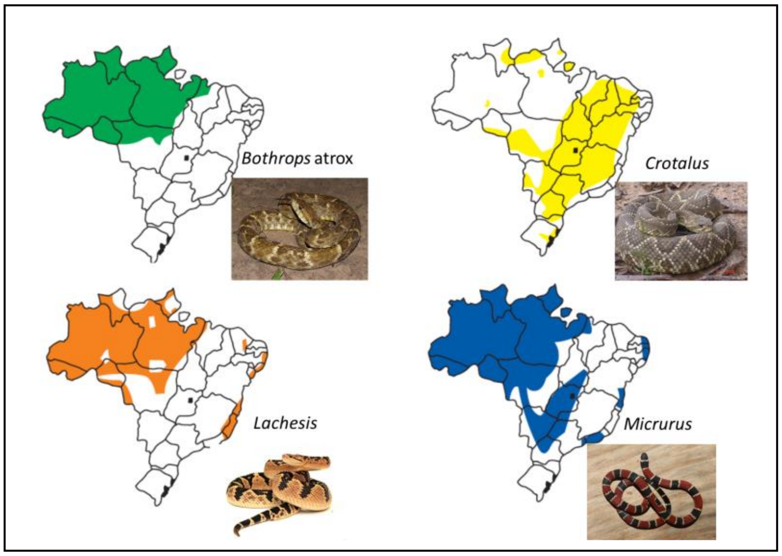

Figura 3 - Distribuição Geográfica das espécies de serpentes *Bothrops*, *Crotalus*, *Lachesis* e *Micrurus* no Brasil (22)

1.1.3 Manifestações Clínicas

A maioria dos acidentes, seja por uma serpente venenosa ou não, leva a algum tipo de efeito local. Pode manifestar-se como uma lesão hiperemiada praticamente sem dor até lesões extremamente dolorosas e severamente edemaciadas, dentro de cinco minutos após o evento. O local acometido também pode apresentar sangramento e bolhas na pele, eventualmente levando à necrose do tecido. Outros sintomas iniciais comuns incluem letargia, sangramento, fraqueza, náuseas e vômitos e que indicam reação sistêmica e maior gravidade do envenenamento. Dependendo do gênero da serpente, os sintomas podem tornar-se mais graves ao longo do tempo, com sinais de hipotensão, taquipneia, taquicardia grave, hemorragia interna grave, sensibilidade alterada, insuficiência renal e falência respiratória (6,20) (Quadro 1).

Quadro 1- Efeitos dos venenos ofídicos de acordo com suas atividades fisiopatológicas (6).

Atividades	Venenos	Efeitos
Inflamatória aguda	Botrópico e laquético	Lesão endotelial e necrose no local da picada<br>Liberação de mediadores inflamatórios
Coagulante	Botrópico, laquético e crotálico	Incoagulabilidade sanguínea
Hemorrágica	Botrópico, laquético	Sangramentos na região da picada (equimose) e à distância (gengivorragia, hematúria, etc.)
Neurotóxica	Crotálico e elapídico	Bloqueio da junção neuromuscular (paralisia de grupos musculares)
Miotóxica	Crotálico	Rabdomiólise (mialgia generalizada, mioglobinúria), incoagulabilidade sanguinea
"Neurotóxica" vagal	Laquético	Estimulação colinérgica (vômitos, dor abdominal, diarreia, hipotensão, choque)

A gravidade clínica do envenenamento por serpente depende da espécie do animal e das características do paciente, mas existem poucos estudos (19) que identificam os subgrupos mais vulneráveis e resultados ainda pouco conclusivos. Na Índia, os fatores relacionados com o paciente, como idade, estado de saúde anterior e tempo decorrido até que assistência médica atue como fatores de risco para gravidade, sequelas e morte (25–29). No mesmo país, os pacientes internados com sintomas de neurotoxicidade e vômitos (26,28), coagulopatia e

leucocitose (27), hipertensão, albuminúria, mudanças no sangramento e tempo de protrombina (29) são considerados os mais propensos a desenvolver desfechos graves. O atraso na administração de antiveneno também foi um fator de risco para gravidade na Nigéria (30). A síndrome de permeabilidade capilar, sangramento e paralisia respiratória indicaram fatores de risco para mortalidade em pacientes que receberam antiveneno serpente na Nigéria e Coreia (31,32).

Na Amazônia, os dados clínicos de envenenamento por *Bothrops atrox* mostraram a dor como a manifestação local mais frequente, seguido por edema e calor à palpação (13,33). Cerca de 25% dos pacientes apresentam manifestações sistêmicas com frequência de sangramento sistêmico espontâneo em 16-18% (13,15,33) e insuficiência renal aguda em 10,9% da uma série de casos (33). Um total de 39,0% dos pacientes desenvolveram complicações secundárias, como a celulite e abscessos (13). Para neutralizar os efeitos do veneno, a administração do antiveneno de acordo com a classificação do acidente possui protocolo definido pelo Ministério da Saúde de acordo com sua gravidade (6) (Quadro 2).

Quadro 2 – Número de ampolas de antiveneno indicada para cada tipo de acidente de acordo com a classificação da gravidade do envenenamento (34).

Acidente	Antiveneno	Gravidade	Nº de ampolas
Botrópico	SAB[b] SABL[c] ou SABC[d]	**Leve**: quadro local discreto, sangramento discreto em pele ou mucosas; pode haver apenas distúrbio na coagulação	2 a 4
		**Moderado**: edema e equimose evidentes, sangramento sem comprometimento do estado geral; pode haver distúrbio na coagulação	4 a 8
		**Grave**: alterações locais intensas, hemorragia grave, hipotensão/choque, insuficiência renal, anúria; pode haver distúrbio na coagulação	12
Laquético[a]	SABL	**Moderado**: quadro local presente; pode haver sangramentos, sem manifestações vagais	10
		**Grave**: quadro local intenso, hemorragia intensa, com manifestações vagais	20
Crotálico	SAC[e] ou SABC	**Leve**: alterações neuroparalíticas discretas; sem mialgia, escurecimento da urina ou oligúria	5
		**Moderado**: alterações neuroparalíticas evidentes, mialgia e mioglobinúria (urina escura) discretas	10
		**Grave**: alterações neuroparalíticas evidentes, mialgia e mioglobinúria intensas, oligúria	20
Elapídico	SAEla[f]	Considerar todos os casos como potencialmente graves pelo risco de insuficiência respiratória	10

[a] Devido à potencial gravidade do acidente laquético, são considerados clinicamente moderados ou graves, não havendo casos leves.
[b] SAB = Soro antibotrópico (pentavalente); [c] SABL = Soro antibotrópico (pentavalente) e antilaquético; [d] SABC = Soro antibotrópico (pentavalente) e anticrotálico; [e] SAC = Soro anticrotálico; [f] SAEla = Soro antielapídico (bivalente).
Fonte: Adaptado do *Manual de Diagnóstico e Tratamento de Acidentes por Animais Peçonhentos* (2001) e do *Guia de Vigilância Epidemiológica* (2009).

Um melhor conhecimento da gravidade e mortalidade por envenenamento de serpente poderia levar a uma melhor gestão, e pode reduzir sequelas e caso taxa de letalidade em localidades remotas na Amazônia brasileira. Para esta realidade, os fatores de risco para gravidade e letalidade causada por envenenamento de serpente no estado do Amazonas foram descritos com base das informações geradas pelas fichas de notificação do SINAN. Os fatores associados a gravidade foram a idade <15 e <65 anos de idade, a área rural como local de ocorrência, acidentes relacionados ao trabalho, procura por assistência médica no período superior a 6 horas do acidente. A letalidade foi descrita como associação para a idade >65 anos e a procura por assistência médica no período maior que 6 horas do acidente (19).

O tempo decorrido entre a picada e o atendimento ao paciente foi principalmente maior que 6 horas (11,19,35), sugerindo que a demora na soroterapia é um fator de risco para gravidade e letalidade. Esta informação é essencial para regular de forma mais efetiva a distribuição de antivenenos de serpentes e terapias de apoio para locais mais úteis e necessários.

Os atrasos na assistência ao paciente, juntamente com o uso de substâncias que podem agravar as condições do envenenamento, aumentando a frequência de complicações locais, resultantes de *Bothrops* e *Lachesis*. No entanto, a gravidade também está possivelmente relacionada à composição dos venenos nas espécies da região amazônica (36).

1.1.4 Complicações

As complicações decorrentes do envenenamento são desencadeadas por fatores intrínsecos como sistema imunológico, nutrição e comorbidades do paciente, bem como de fatores extrínsecos relacionados ao ambiente e condutas no local do acidente realizadas como torniquete e terapias alternativas que potencializam a ação do veneno e de micro-organismo presentes na flora oral da serpente (37,38).

Desta forma, a evolução do paciente com piora do quadro, frequentemente estão relacionadas às injúrias renais 15.0 a 38.0% (29,39–42), com o desenvolvimento de necrose local 10 a 38.5% (11,37,42) e infecção secundária de 11 a 39% dos casos (11,33,37,42–44). Essas complicações podem necessitar de procedimentos médicos como debridamento, amputações e diálise (3,7,11,45–47) (Fig. 4).

Além da evolução patológica, existe também a perda socioeconômica nos acidentes ofídicos (6,48) que ainda é uma questão totalmente negligenciada no Brasil. Na Amazônia brasileira, os dados disponíveis também não retratam as consequências físicas e psicológicas a longo prazo de sobrevivência pós acidente ofídico (49–51). Porém, como a maioria das vítimas está na faixa etária economicamente produtiva, o impacto econômico deste agravo possivelmente é relevante (51).

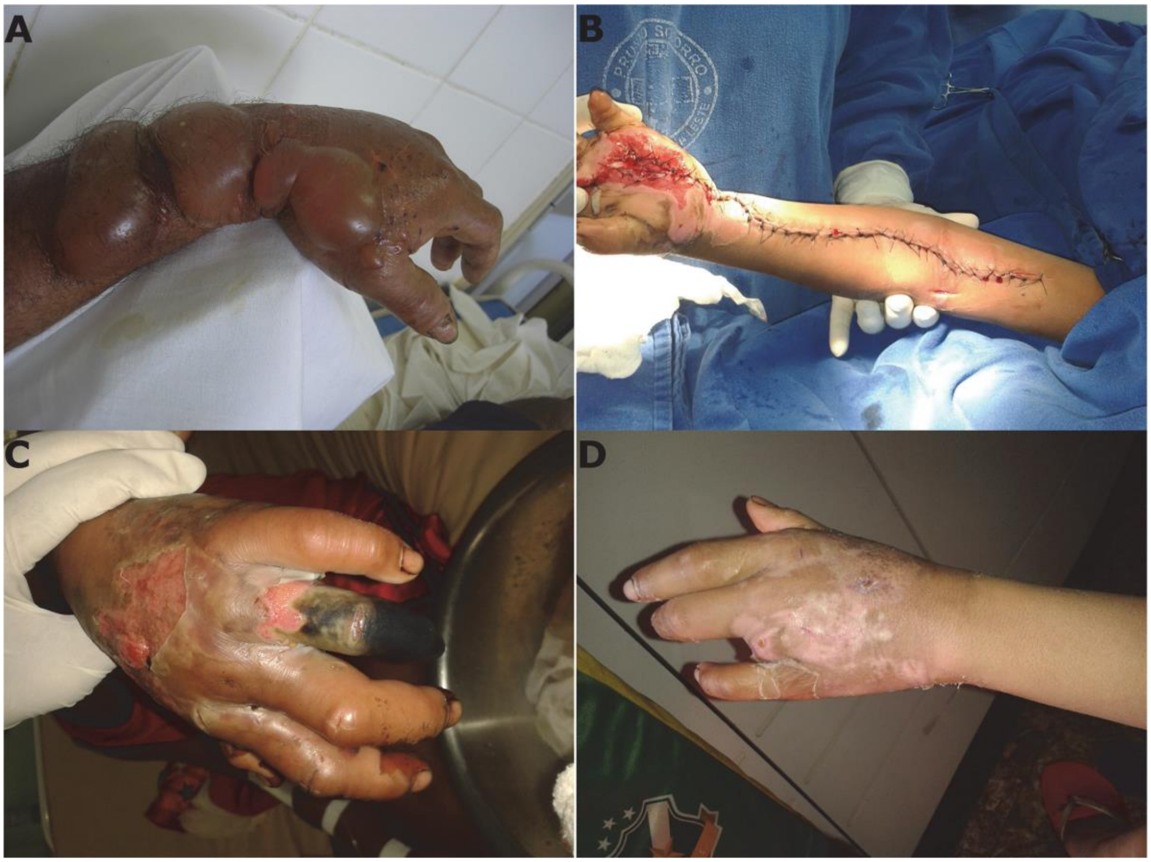

Figura 4 - Complicações locais, resultantes de acidentes botrópicos. A) envenenamento na mão; esse paciente chegou 12 horas após a picada no Hospital Municipal de Belterra, com inchaço e bolhas sero-hemorrágicas no membro superior esquerdo e sangue incoagulável. B) envenenamento grave na mão esquerda; esse paciente chegou 24 horas após a picada na FMT-HVD, apresentando síndrome compartimental no membro superior esquerdo, exigindo

fasciotomia. C) envenenamento na mão esquerda, o paciente chegou 24 horas após a picada na FMT-HVD, com uma extensa área de edema e necrose no membro superior esquerdo e gangrena do quarto dedo. D) O mesmo paciente mostrado em C, após amputação do quarto dedo (na fase de cura) (21).

1.2 Infecções Secundárias no acidente ofídico

1.2.1 Frequência

Atualmente, muito pouco se sabe sobre a frequência de infecções secundárias, bem como o espectro de bactérias responsáveis por infecções de feridas em pacientes ofídicos ou mesmo a susceptibilidade aos antimicrobianos. Alguns estudos evidenciam pontualmente a ocorrência das infecções secundárias após os acidentes ofídicos (13,33,52–56), caracterizando as lesões e manifestações clínicas.

A frequência dessas infecções associada a flora oral da serpente, também é descrita como uma complicação multifatorial caracterizada principalmente pela presença de fatores como aplicação de torniquete e utilização de terapias locais alternativas (ervas, óleos e ingredientes indefinidos). Além disso, outras condutas também podem contribuir para o surgimento das infecções como a ingestão de misturas nativas, incisões no local do acidente, sucção da lesão entre outros (7,15,38)

Alguns medicamentos refletem um misticismo em torno do envenenamento ofídico. Nas regiões de acesso remoto, essas terapias alternativas são muito utilizadas e encontradas facilmente nessas comunidades como é o caso do popular ‘Especifico Pessoa’. Este composto é citato como possuidor de componentes que atuam contra determinados venenos de serpente (7).

As terapias alternativas, quando utilizadas, retardam a procura por atendimento médico, pois inferem uma expectativa de cura. Assim, a vítima está susceptível à progressão da gravidade e surgimento de complicações com esse aumento do tempo entre a hora do acidente e a assistência hospitalar para administração do antiveneno (15,19,29,42).

Contudo, a associação destes fatores potencializam o desenvolvimento das infecções secundárias de forma expressiva, diagnosticada primordialmente com a presença de celulites e/ou abscesso (33,37,57) (Fig. 5).

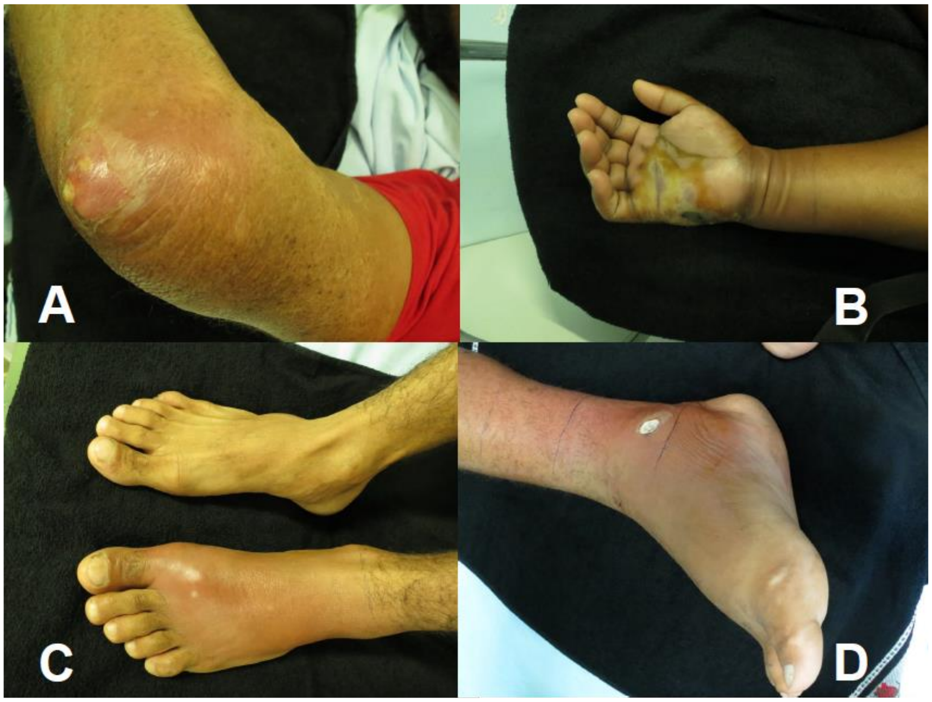

Figura 5 – Acidentes ofídicos com infecção secundária A-D. A) Formação de abscesso com drenagem de secreção purulenta em membro superior direito; B) Presença de necrose e secreção sero-purulenta em mão direita; C) Formação de celulite com tecido flutuante e rubor intenso em dorso do pé esquerdo; D) Infecção com associação de celulite e abscesso após colocação de dreno de penrose em tornozelo do membro inferior direito (Arquivo próprio).

A infecção secundária possui uma ocorrência variada, devido a presença ou ausência dos fatores influenciadores na população estudada. Em Taiwan os pacientes apresentaram esta complicação em 44% dos casos, necessitando de intervenção cirúrgica (44). Na Nigéria, 41.7% das vítimas foram tratadas para infecção da ferida definida como eritema, calor local, secreção purulenta e febre (38). Na Colômbia as infecções foram identificadas em 30.8% dos acidentes (42) e 2,5% nos acidentes ocorridos na Índia (27).

No Brasil, as infeções secundárias tiveram prevalência 15.3% para acidentes botrópicos e laquéticos em Tocantins (53), 4.7% em acidentes botrópicos em São Paulo/Minas Gerais (58),

15,7% dos pacientes em Goiânia evoluíram com formação de abscesso no local da picada, com lesão flutuante e secreção purulenta ou sero-purulenta (59). Em vítimas atendidas no Hospital Vital Brazil do Instituto Butantan em São Paulo, foram reportados dois estudos de acidentes botrópicos que mostraram 9,2% e 15,3% de infecções secundárias (60,61). Em crianças admitidas no Hospital Universitário da UNICAMP também observou-se o desenvolvimento de infecção (15,1%) em acidentes botrópicos (62).

Nos acidentes botrópicos da região amazônica houveram prevalência de infeção de 8,3% ocorridos em municípios do interior do Amazonas (11) e 39,0% nas vítimas atendidas na Fundação de Medicina Tropical Dr. Vieira Dourado em Manaus (33,42).

Um estudo realizado na Costa Rica com 80 crianças avaliou a formação de abscesso em 11,25% dos casos e identificou fatores de risco para essa evolução como: protrombina <2%, fibrinogênio <100g/dl, habitantes de zonas mais afastadas e lesões ocorridas na parte mais proximal do corpo (37).

1.2.2 Micro-organismo envolvidos

A flora oral de serpente compreende uma ampla gama de microrganismos aeróbios e anaeróbios. Os micro-organismos encontrados nas presas de diversas serpentes como *Bothrops*, víboras, cascavéis e naja, mostraram colonização de várias bactérias, incluindo Enterobactérias, *Morganella* spp. e *Escherichia coli*, *Estreptococos*, *Aeromonas* spp., *Staphylococcus aureus*, e anaeróbios tais como *Clostridium* spp. (54,57,63).

A cavidade oral de 15 cascavéis tiveram bactérias cultivadas, sendo encontradas 58 cepas aeróbias e 28 cepas anaeróbias. As espécies mais comuns isoladas foram *Pseudomonas aeruginosa*, espécies *Proteus*, *Estafilococos* coagulase-negativo, e as espécies de *Clostridium* (64). Nas culturas de *Bothrops jararaca* também foram isoladas *Salmonella* (27.3%), *Citrobacter* (26.0%) e *Escherichia* (12.3%) (65).

Estudos mostram que, normalmente, tanto bactérias aeróbias quanto bactérias anaeróbias são responsáveis pelos abscessos, sendo que há predomínio de enterobactérias aeróbias (principalmente *Morganella morganii*) (22,54,66). As bactérias isoladas em abscessos podem incluir espécies presentes na boca e no veneno das serpentes (67), bem como na pele das vítimas, tais como *Staphylococcus aureus* (42,68).

Os abscessos das vítimas de acidentes ofídicos também tem sido estudados para identificar o agente causador das infecções secundárias. Os bacilos Gram negativos aeróbicos foram os mais frequentemente identificados, destacando-se a *Morganella morganii* isolada em 44,4% das culturas, *Escherichia coli* 20,2%, *Providencia* sp 13,1% e *Staphylococcus aureus* (8,1%) (59).

1.2.3 Profilaxia e tratamento

Neste contexto, a referência dos estudos concentram-se na terapêutica medicamentosa para a infecção secundária com utilização de diversos antimicrobianos, que apresentam variáveis relacionadas ao acidente ofídico como a espécie da serpente e a composição da sua flora oral (55,69). A escolha do antibiótico também apresenta diversidade terapêutica com indicação de Ampicilina/Cefalosporina/Cloxacilina (38,43), Ciprofloxacino (44,57), Clindamicina (69–71), entretanto, alguns estudos descrevem a sua antibioticoprofilaxia como controversa devido a inexistência de protocolo específico para o acidente ofídico (57,72).

A antibioticoprofilaxia para infecção secundária em acidentes com serpentes ainda necessita de evidência científica para o estabelecimento da sua eficácia (73,74). Em contrapartida, a prevenção de infecções com antibioticoprofilaxia em outras situações como procedimentos cirúrgicos já possuem protocolos comprovados com ensaios clínicos randomizados (75,76). Desta forma, estes pacientes se beneficiam com a diminuição da morbidade infecciosa de procedimentos cirúrgicos por administração precoce de doses de antibióticos (73,77).

O uso profilático de antibióticos no acidente ofídico tem sido defendido e recomendado por alguns autores (64), baseados em evidências sobre a flora bacteriana da cavidade oral e no veneno das serpentes, bem como a administração de antibióticos de amplo espectro em casos de acidentes graves (69). A pele da vítima, o vestuário, os materiais utilizados para os primeiros socorros e o meio ambiente hospitalar também foram implicados como outras eventuais fontes de infecção bacteriana (22,37,78). Assim, as infecções secundárias desenvolvidas em pacientes classificados com acidentes graves são, à princípio, aqueles que poderão ter maiores benefícios com a profilaxia antibiótica (69,79).

Nos estudos de casos de celulite secundária à mordedura de serpente no Zimbabwe, foi recomendado que antibióticos da família das penicilinas devem ser frequentemente utilizados profilaticamente (72).

Entretanto, as evidências clínicas para a prevenção da infecção secundária em acidente ofídico em seres humanos foram reportadas em apenas dois estudo com randomização de 2 grupos (grupo intervenção e grupo controle). Um dos estudos foi realizado com acidentes envolvendo o gênero *Bothrops* no Hospital Vital Brazil, sendo que, o grupo experimental recebeu a monoterapia com cloranfenicol oral para verificar sua eficácia em prevenir infecções secundárias das lesões (58). Após o acompanhamento da evolução clínica, concluiu-se que não houve diferença significativa na ocorrência de infecções locais. Entretanto o desfecho estudado foi abscesso e não outros tipos de infecção bacteriana, tal como a celulite localizada.

O segundo estudo foi com acidentes envolvendo o gênero *Crotalus* na floresta Amazônica do Equador e os pacientes receberam como intervenção a administração profilática de gentamicina intravenosa associada ao cloranfenicol (73). Os resultados também não mostraram diferenças estatisticamente significativas entre os pacientes tratados com antibióticos e não tratados. Assim, o uso rotineiro de antibióticos profiláticos para a prevenção das complicações infecciosas do envenenamento crotálico não pode ainda ser recomendado.

1.3 Justificativa

O tratamento médico de infecção secundária, necrose e síndrome compartimental tem sido objeto de controvérsia, em parte por causa da falta de padronização sobre conceitos e protocolos de gestão. A possibilidade de reduzir os efeitos locais por meio de medicamentos com atividade anti-inflamatória, antibioticoterapia precoce para infecção secundária e novos tratamentos complementares precisam ser mais bem investigada, observando boas práticas clínicas e, de preferência, em estudos multicêntricos. As complicações sistêmicas, tais como sepse e lesão renal aguda são menos conhecidos e a falta de acompanhamento do paciente, incluindo testes laboratoriais, parece estar relacionado a esta observação (21).

Atualmente, a redução de complicações sistêmicas com a prevenção das infecções secundárias por mordida de animais foi recomendada pela Infectious Diseases Society of America (IDSA) com utilização de Inibidor da Betalactamase (70,71). Este guideline evidencia um estudo clínico que mostrou proteção significativa ao utilizar o referido antibiótico de forma profilática em mordida de animais (74), entretanto, a comprovação científica para eficácia deste medicamento em acidentes envolvendo animais peçonhentos ainda não foi evidenciada.

A Amoxicilina/Inibidor da Betalacmase (Clavulin BD®) tem ação sobre bactérias Gram-negativas, estafilococos e alguma ação sobre anaeróbios, o que torna esse antibiótico igualmente atrativo para ser usado de forma precoce. Possui boa absorção oral, boa distribuição em partes moles e tem posologia facilitada, além de estar facilmente disponível nos mais diversos municípios, em função da sua indicação para várias outras infecções bacterianas (70,71).

Diante deste contexto, suscita a necessidade de novas investigações utilizando antimicrobianos disponíveis nas redes básicas de atendimento de saúde, com o propósito de estabelecer condutas clínicas preventivas que contribuam para a diminuição de agravos e maior resolutividade e prevenção dos danos à saúde, relacionado à infecção decorrente dos acidentes ofídicos.

2 OBJETIVOS

2.1 Objetivo Geral:

- Avaliar a eficácia da antibioticoterapia na prevenção de infecções secundárias em pacientes vítimas de acidentes ofídicos.

2.2 Objetivos Específicos:

- Identificar o perfil sociodemográfico dos pacientes envolvidos em acidentes ofídicos;

- Identificar o perfil clínico dos pacientes envolvidos em acidentes ofídicos;

- Estimar a redução na incidência de infecções secundárias em pacientes vítimas de envenenamentos ofídicos tratados com o Amoxicilina/Ácido Clavulânico.

3 MATERIAIS E MÉTODOS

3.1 Modelo de estudo

Trata-se de um ensaio clínico randomizado com pacientes vítimas de envenenamento ofídicos atendidos na Fundação de Medicina Tropical Doutor Heitor Vieira Dourado, acometidos por acidentes ofídicos no período de julho/2014 a julho/2016.

3.2 Local de estudo

A Fundação de Medicina Tropical Doutor Heitor Vieira Dourado (FMT-HVD) está situada no município de Manaus-AM, sendo considerada centro de referência nacional e mundial para o tratamento de enfermidades tropicais, principalmente aos eventos relacionados a animais peçonhentos.

Atualmente, representa o centro de referência para doenças infecciosas, parasitárias e dermatológicas do estado do Amazonas, com 40 consultórios médicos e mais de 250.000 consultas de saúde / ano; 150 leitos, sendo 10 leitos em UTI; 14 leitos dedicados à pesquisa clínica; prontuário eletrônico para informação médica com sistema iDoctor®, permitindo o monitoramento mais rápido em ensaios clínicos; Laboratório de Análises Clínicas e Equipe treinada em Boas Práticas Clínicas) (Fig. 6).

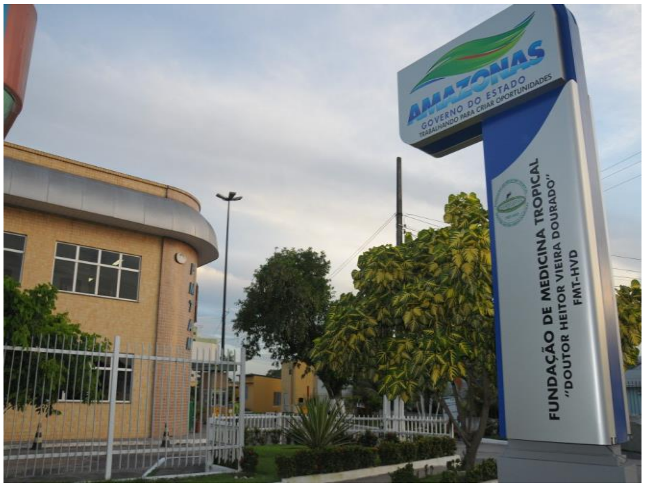

Figura 6 – Entrada da Fundação de Medicina Tropical Doutor Heitor Vieira Dourado (Arquivo próprio).

Além disso, desde 2005, a Fundação compõe a Rede Nacional de Pesquisa Clínica em Hospitais de Ensino (RNPC) que se trata de uma iniciativa do Ministério da Saúde (MS) e da Ciência e Tecnologia (MCT) para promover as melhores práticas de pesquisa voltadas às necessidades do Sistema Único. A rede prioriza o desenvolvimento de ensaios clínicos de medicamentos, procedimentos, equipamentos e dispositivos diagnósticos, de interesse para o Sistema Único de Saúde. O objetivo desta rede é que a capacitação regional proveniente da integração nacional dirigida promova a aceleração do crescimento e da capacidade científica de cada um destes centros. Desse modo, os centros estarão aptos a desenvolver ferramentas próprias com capacidade de solucionar desafios específicos do nosso país.

Neste sentido, os indivíduos atendidos na rede hospitalar do estado do Amazonas serão encaminhados para o serviço em questão para o tratamento e acompanhamento dos envolvidos em acidentes ofídicos, pois, esta unidade é referência para esse tipo de acidente no Estado. Em Manaus, a FMT-HVD é a única unidade hospitalar que realização a dispensação de antivenenos ofídicos.

Os acidentes ofídicos hospitalizados na FMT-HVD no período de 1974 a 1984 registraram um total de 514 vítimas. A Figura 7 mostra a série casos de 1974 a 2012 com aumento da incidência de acidentes ofídicos na década de 1990, que atingiu o pico em 1998, com 554 casos na FMT-HVD. Desde o início, a vigilância epidemiológica dos acidentes ofídicos no Estado do Amazonas mostrou que atividades rurais estão intimamente associados com a ocorrência deste problema. Na década de 1990, o desenvolvimento da silvicultura sustentável, a pesca e a agricultura no Estado foi estimulado como resultado do apoio oficial do governo para a criação do Programa Zona Franca Verde (9).

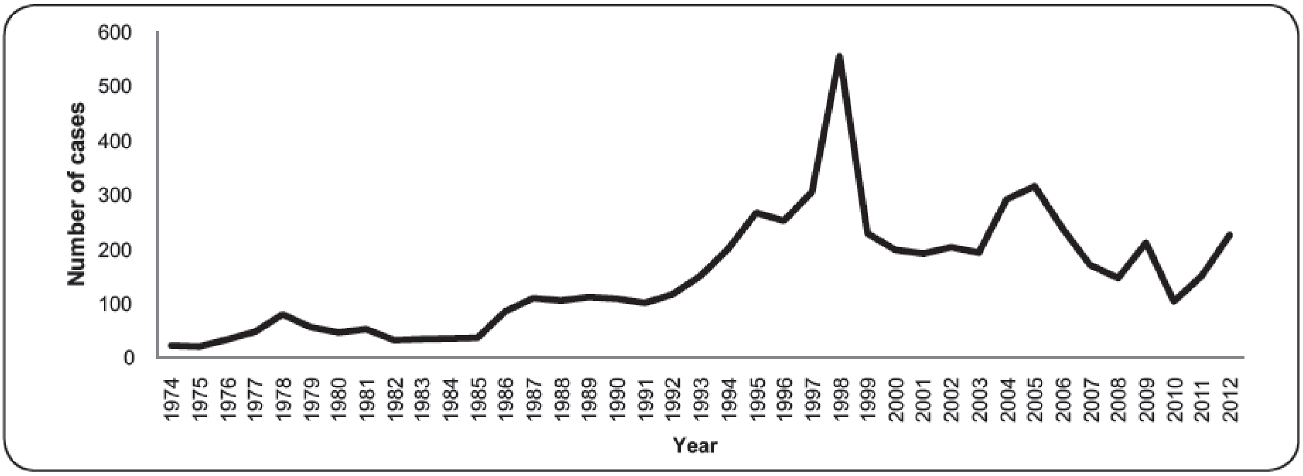

Figura 7 – Evolução histórica dos acidentes com serpentes ocorridos na Fundação de Medicina Tropical Doutor Heitor Vieira Dourado, Manaus (1974-2012) (9)

3.3 Plano amostral

A população considerada para o presente estudo corresponde aos pacientes atendidos pela instituição com acidentes ofídicos, correspondendo em média 240 pacientes/ano.

O tamanho amostral foi definido levando em consideração os seguintes parâmetros:

a) Nível de Confiança de 95%;
b) Poder de 80%;
c) Frequência esperada de infecção secundária 40% (33)
d) 50% de redução do risco de infecção;
e) Razão 1:1 entre os pacientes do grupo experimental e grupo controle;
f) Acréscimo de no mínimo 10% de perdas de seguimento.

Dessa forma, foi estimada uma amostra de 93 pacientes para o grupo experimental e 93 pacientes para o grupo controle.

3.4 Critérios de Elegibilidade

Os pacientes para serem elegíveis para o estudo deverão cumprir as seguintes características:

a) ter menos de 24 horas após o acidente;
b) não ter utilizado qualquer antibiótico nos últimos 30 dias antes do atendimento na instituição;
c) não ter realizado a soroterapia para o acidente ofídico atual;
d) não possuir abscesso ou infecção claramente estabelecida no momento da admissão; ser alérgico ao antibiótico de escolha neste estudo;
e) não estar gestante e;
f) aceitar participar do estudo.

3.5 Procedimento de Randomização

Os pacientes incluídos serão randomizados de forma aleatória simples em dois grupos por tabela randomizada (APÊNDICE A), sendo um grupo em esquema tradicional de acompanhamento clínico (sem profilaxia antibiótica) e outro no grupo com tratamento proposto para infecção secundária (com profilaxia de Amoxicilina/Inibidor da Betalactamase).

3.6 Intervenção

O antibiótico de escolha será a Amoxicilina/Inibidor da Betalactamase – Clavulin BD® (875/125 mg) por 7 (sete) dias, a contar da data da admissão. A posologia para o indivíduo adulto será 1 comprimido de 12/12 horas e para infantil será utilizado o medicamento na forma de suspensão oral pelo cálculo 25/3,6 mg/kg/dia (Quadro 3).

Quadro 3 – Posologia para administração da profilaxia do Clavulin BD®

**Posologia – Infantil 25/3,6 mg/kg/dia**	2 – 6 anos (13 - 21 Kg)	5 ml de Clavulin® BD Suspensão 200mg+28,5mg/5ml ou 2,5 ml de Clavulin® BD Suspensão 400mg+57/5ml	2 x ao dia
	7 – 12 anos (22 - 40 Kg)	10 ml de Clavulin® BD Suspensão 200mg+28,5mg/5ml ou 5 ml de Clavulin® BD Suspensão 400mg+57/5ml	2 x ao dia
**Posologia – Adulto 875/125 mg**	> 12 anos (mais de 40 Kg)	1 comprimido de Clavulin® BD 875/125mg	2 x ao dia

3.7 Seguimento dos Pacientes

Antes da administração do antiveneno, os pacientes incluídos responderão a um questionário semiestruturado contendo variáveis sociodemográficas (sexo, idade, endereço, peso) e clínico-epidemiológicas (data e horário do acidente, local do corpo afetado, classificação do acidente, condutas pré-hospitalares, sinais e sintomas locais e sistêmicos, entro outros).

A avaliação do local do acidente será da seguinte forma:

a) Dor: intensidade avaliada por escala numérica 0-10;
b) Edema: utilização de fita métrica (escala em centímetros) para circunferência do membro afetado na proximidade do local do acidente e no membro contralateral. A extensão do edema também será avaliada para mensurar a distância próxima e distal da mordedura da serpente (Fig. 8);

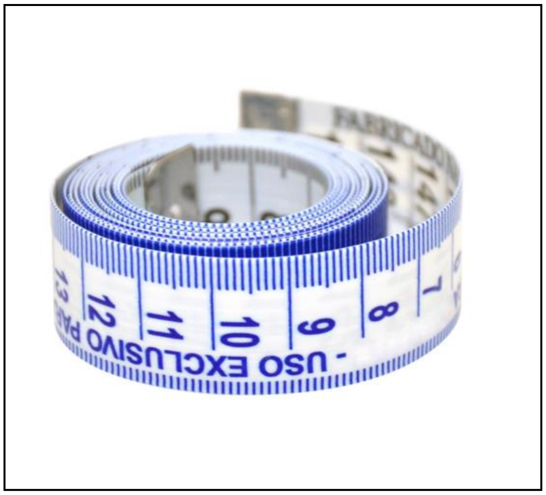

Figura 8 – Fita métrica com escala em centímetro. Fonte: http://www.google.com.br

c) Temperatura: será mensurada por termômetro clínico digital infravermelho MODELO COLOR CHECK AC322, a aferição será realizada o mais próximo do local da picada e na região anatômica idêntica do membro contralateral (Fig. 9).

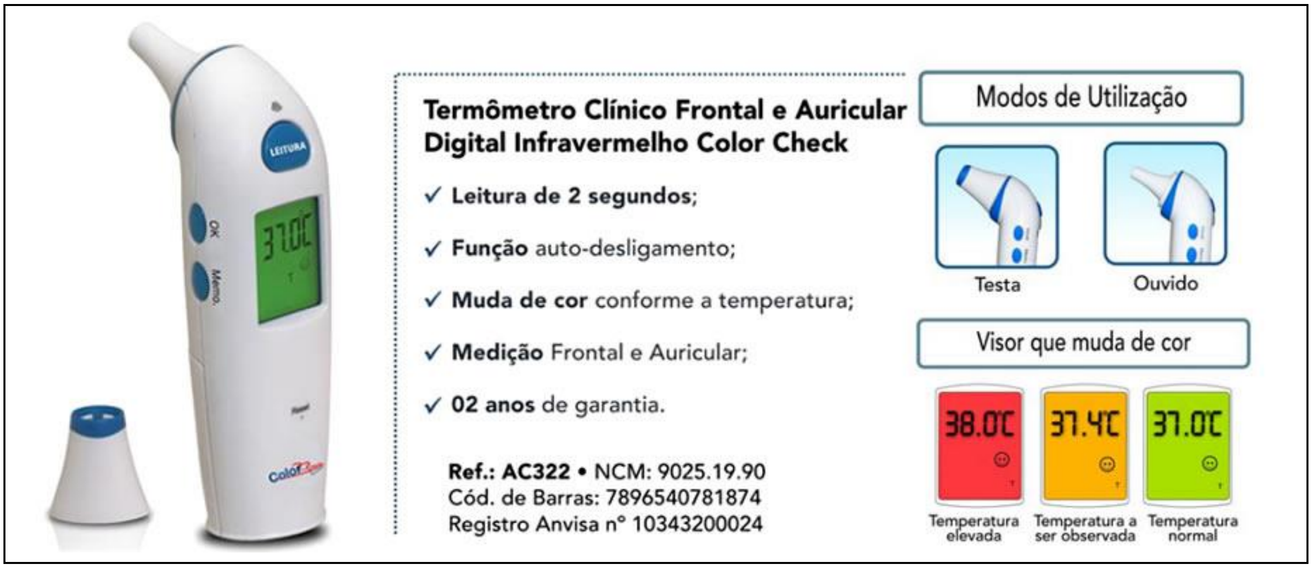

Figura 9 – Termômetro Clínico Digital Infravermelho. Fonte: http://www.medjet.com.br/

Neste momento, também serão submetidos à uma coleta de 15 ml de sangue para a realização dos exames laboratoriais listados no Quadro 2. Novas coletas serão realizadas nos tempos de 24h, 48h, 72h e 7° dia após admissão para a realização destes exames, exceto para a venenemia (Quadro 4).

Quadro 4 – Descrição dos exames laboratoriais realizados nos pacientes acompanhados durante 7 dias de acompanhamento.

Tipo de exame	Periodicidade da coleta/análise	Local de realização
- Teste de Elisa – Venenemia	Somente na admissão antes da administração do antiveneno	Butantan
- Hemograma completo, Plaquetas.<br>- Potássio, Sódio, Ureia e Creatinina.<br>- AST/ALT<br>- Velocidade de Hemossedimentação<br>- Proteína C reativa<br>- CK e CK MB<br>- EAS	Na admissão – 24h – 48h – 72h – e 7° dia.	FMT-HVD
- Fibrinogênio;<br>- TAP;<br>- Tempo de coagulação.	Na admissão – 24h – 48h – 72h – e 7° dia.	HEMOAM

Para maiores detalhes sobre o seguimento dos pacientes, consultar o Protocolo de Pesquisa (APÊNDICE B).

Os dois grupos serão inicialmente internados por 3 (três) dias e com apenas um retorno no 7° (sétimo) dia após a inclusão no estudo em atendimento ambulatorial. O grupo com tratamento convencional receberá a assistência de acordo com o protocolo da instituição para acidentes ofídicos. O grupo com a proposta de intervenção receberá o mesmo tratamento estabelecido, sendo adicionado a antibioticoterapia precoce.

3.8 Identificação específica e determinação da Venenemia

A identificação e determinação da venenemia será realizada pelo exame de ELISA com AcMo anti-*B*. *atrox* + biotina que será realizado no Instituto Butantan.

As placas serão sensibilizadas com 25 µg/ml de Soro anti-botrópico/laquético (SBL) do Instituto Butantan em PBS “over night”. As placas serão lavadas e bloqueadas com PBS contendo 2% de BSA (2hs/37ºC) e em seguida incubadas com curvas de veneno homólogo ou hetrólogo diluídas em PBS/BSA 1% ou PBS/BSA 1% contendo soro normal humano diluído 1/5 (2 e ½ hs/37ºC). Após lavagem, as placas serão incubadas com IgG monoclonal de camundongo anti-*B*. *atrox* conjugada à biotina diluída 1/250 em PBS/BSA 1% contendo 0,05% de Tween 20 ou com um pool de IgGs monoclonais de camundongo anti-*L. muta* conjugadas à biotina diluída 1/500 no mesmo tampão (2 e ½ hs/37ºC). As placas serão lavadas e incubadas com estreptoavidina-perosidase na diluição de 1/250, para o AcMo anti-*B. atrox* ou 1/500, para o pool de AcMo anti-*L. muta*, diluídos em PBS/BSA 1% contendo 0,05% de Tween 20 (30 min./37ºC). As placas serão reveladas com adição do substrato cromogenico OPD e H_2O_2. A leitura da densidade óptica será feita a 490nm.

3.9 Definição de Infecção Secundária

A infecção secundária ao acidente ofídico, definida para este estudo como a presença de celulite e/ou abscesso (70,71) até 48 horas após a admissão em qualquer um dos grupos será o desfecho primário. Nestes casos, será administrado para tratamento o antibiótico Clindamicina EV 600mg de 6/6 horas ou conforme resultado do antibiograma para aqueles pacientes dos quais foi possível isolar o micro-organismo. O surgimento de infecção secundária após 48 horas da admissão será considerado como um desfecho secundário e seu tratamento se dará da mesma forma descrita acima.

Para tanto, a celulite será definida pela presença de sinais locais de inflamação (eritema, edema, calor e dor) associada a febre, leucocitose, linfangite e/ou linfadenite (80). O abscesso será caracterizado pela presença de lesão individualizada, flutuante, apresentando secreção purulenta ou sero-purulenta (59,80).

Os indivíduos identificados com infecção secundária serão adicionalmente avaliados por ultrassonografia e biópsia da ferida (para realização de coloração pelo Gram e histopatologia). A identificação da bactéria no material coletado será realizada por cultura automatizada com antibiograma.

Após a alta o paciente será instruído para que retorne à FMT-HVD quando houver alguma modificação ou complicações relacionada ao acidente ofídico.

3.10 Questões Éticas

O estudo foi submetido ao Comitê de Ética em Pesquisa da Universidade do Estado do Amazonas (CAAE: 19380913.6.0000.5016 e Número do Parecer: 492.892) (ANEXO I) e da FMT-HVD (CAAE: 19380913.6.3001.0005 e Número do Parecer: 602.907-0) (ANEXO II) e foi submetido ao Registro Brasileiro de Ensaios Clínicos (REBEC).

Os pacientes assinarão o TCLE (APÊNDICE C) para participação da pesquisa. Para os indivíduos menores que 18 anos deverão assinar o Termo de Assentimento (APÊNDICE D) e também ter o consentimento dos pais ou responsáveis.

3.11 Plano Analítico

As variáveis quantitativas serão calculados frequência e proporções e para as variáveis qualitativas serão categorizadas e realização do cálculo de médias, além disso, serão utilizados testes estatísticos para definir associação entre as variáveis por meio da Curva de Sobrevida, Regressão Logística, Qui-quadrado e Teste Exato de Fisher. Serão considerados IC 95% e $p>0,05$.

Para as estimativas da eficácia do uso de profilaxia antibiótica, serão obtidas as seguintes medidas (75,81), incluindo uma análise por intenção de tratamento:

Odds Ratio (OR): número de infecções secundárias que ocorrem com antibioticoprofilaxia dividido pelo número de infecções secundárias que ocorrem sem profilaxia.

Risco Básico Esperado: número de infecções secundárias ao acidente ofídico no hospital (média anual dos últimos 5 anos), divididas pelo número total de acidentes ofídicos que ocorreram no ano (média anual dos últimos 5 anos). O risco básico esperado multiplicado por 100 é o risco percentual de infecção secundária o acidente ofídico.

Números necessários para tratamento efetivo (NNT): número de pacientes que devem ser administrado profilaxia com antibióticos a fim de impedir uma infecção secundária da ferida.

$$NNT = \frac{1- [\text{Risco Básico Esperado} \times (1\text{-OR})]}{(1\text{-Risco Básico Esperado}) \times \text{Risco Básico Esperado} \times (1\text{-OR})}$$

4 ORÇAMENTO

ITENS DE CUSTEIO (COM JUSTIFICATIVA)	QUANTIDADE	VALOR UNIT. (R$)	VALOR TOTAL (R$)
Reagentes para teste de ELISA para detecção do veneno ofídico	1	25.000,00	25.000,00
Exames de Hematologia e bioquímica para acompanhamento clínico e evolução da infecção	1	10.000,00	10.000,00
kits para dosagens de Pró-calcitonina para acompanhamento clínico	1	18.000,00	18.000,00
Material para realização de pesquisa direta de cultura para diagnóstico da infecção secundária	1	10.000,00	10.000,00
kits para dosagens de Fatores de Coagulação para acompanhamento clínico	1	34.000,00	34.000,00
Kits para realização de Histopatologia para acompanhamento clínico e evolução da infecção	1	10.000,00	10.000,00
		**Subtotal A**	**R$ 107.000,00**
**ITENS DE CAPITAL (COM JUSTIFICATIVA)**	**QUANTIDADE**	**VALOR UNIT. (R$)**	**VALOR TOTAL (R$)**
Notebook (para compilação e análise de dados)	1	2.500,00	2.500,00
		**Subtotal B**	**R$ 2.500,00**
**BOLSA**	**QUANTIDADE**	**VALOR UNIT. (R$)**	**VALOR TOTAL (R$)**
bolsa por projeto na modalidade Desenvolvimento Científico Tecnológico Amazônico - DCTA, nível C<br>Ter título de Mestre, ou Técnico de nível superior com 2 (dois) anos de experiência em projetos de C&T; Dedicar, no mínimo, 30 (trinta) horas semanais às atividades a serem desenvolvidas; Não ter vínculo empregatício com carga horária semanal superior a 12 (doze) horas.	18	1.234,00	22.212,00
		**Subtotal**	**R$ 22.212,00**
		**TOTAL GERAL**	**R$ 131.712,00**

* Este Projeto será financiado pela FAPEAM conforme consulta pública no link http://www.fapeam.am.gov.br/decisoes-conselho/ da DECISÃO 287/2013. (ANEXO III)

5 CRONOGRAMA

ATIVIDADES	ANO/TRIMESTRE												
	2013	2014				2015				2016			
	4	1	2	3	4	1	2	3	4	1	2	3	4
Revisão do Projeto e procedimentos	**X**	**X**	**X**										
Recrutamento de pacientes envolvidos em acidentes com animais peçonhentos				**X**	**X**	**X**	**X**	**X**	**X**	**X**	**X**		
Realização de exames clínico-laboratoriais e de cultura				**X**	**X**	**X**	**X**	**X**	**X**	**X**	**X**		
Análise dos Dados							**X**	**X**			**X**	**X**	
Avaliações parciais									**X**				
Redação dos artigos									**X**	**X**	**X**	**X**	**X**
Preparo de relatório final													**X**
**Desembolso de recurso para compra de material permanente**				**X**									
**Desembolso de recurso para compra de material de consumo**				**X**	**X**								

6 PARTICIPANTES DO PROJETO

	Nome<br>(ordem alfabética)	Categoria	Formação	Instituição de vínculo
1	Ana Maria Moura	Pesquisadora	Bioquímica	Butantan
2	Eliane Alves	Mestranda	Enfermeira	FMT-HVD
3	Fábio Francisconi do Valle	Pesquisador	Médico Dermatologista	FMT-HVD
4	Iran Mendonça da Silva	Doutorando	Médico	FMT-HVD
5	Jacqueline A. Gonçalves Sachett	Doutoranda	Enfermeira	FMT-HVD/UEA
6	Geraldo Majela Soares	Pesquisador	Bioquímico	FMT-HVD
7	Antônio Magela Tavares	Pesquisador	Médico Infectologista	FMT-HVD
8	Gustavo Adolfo Sierra Romero	Pesquisador	Médico	UNB
9	Hedylamar Marques	Pesquisadora	Bioquímica	HEMOAM
10	Luiz Carlos de Lima Ferreira	Pesquisador	Médico Patologista	FMT-HVD
11	Marcelo Cordeiro dos Santos	Pesquisador	Médico Infectologista	FMT-HVD
12	Marcus Vinícius G. Lacerda	Pesquisador	Médico Infectologista	FMT-HVD
13	Mônica Colombini	Pesquisador	Médica Veterinária	Butantan
14	Sâmella Silva de Oliveira	Doutoranda	Enfermeira	FMT-HVD
15	Silvio Cesar Pereira Fragoso	Pesquisador	Médico Infectologista	FMT-HVD
16	Wuelton Marcelo Monteiro	Pesquisador	Farmacêutico-Bioquímico	FMT-HVD

7 REFERÊNCIAS

1. White J. Bites and stings from venomous animals: a global overview. Ther Drug Monit. 2000;22(1):65–8.
2. Chippaux JP. Snake-bites: Appraisal of the global situation. Bull World Health Organ. 1998;76(5):515–24.
3. Gutiérrez JM, Theakston RDG, Warrell D a. Confronting the neglected problem of snake bite envenoming: the need for a global partnership. PLoS Med [Internet]. 2006 Jun [cited 2014 Dec 19];3(6):e150. Available from: http://www.pubmedcentral.nih.gov/articlerender.fcgi?artid=1472552&tool=pmcentrez&rendertype=abstract
4. Kasturiratne A, Wickremasinghe AR, Silva N, Gunawardena NK, Pathmeswaran A, Premaratna R, et al. The Global Burden of Snakebite: A Literature Analysis and Modelling Based on Regional Estimates of Envenoming and Deaths. PLoS Med [Internet]. 2008 [cited 2015 Jan 2];5(11):1591–604. Available from: http://www.rexano.org/ResponsibleOwnership/10.1371_journal.pmed.0050218-S.pdf
5. Brasil. SISTEMA DE INFORMAÇÃO DE AGRAVOS DE NOTIFICAÇÃO - SINAN [Internet]. Ministério da Saúde. 2014 [cited 2015 Jan 2]. Available from: http://dtr2004.saude.gov.br/sinanweb/
6. Brasil. Caderno 14-Acidentes por Animais Peçonhentos. In: Saude M da, editor. Guia de vigilância epidemiológica [Internet]. 7th ed. Brasília: Ministério da Saúde; 2009. p. 23. Available from: http://bvsms.saude.gov.br/bvs/publicacoes/guia_vigilancia_epidemiologica_7ed.pdf
7. Pierini S V, Warrell DA, de Paulo A, Theakston RD. High incidence of bites and stings by snakes and other animals among rubber tappers and Amazonian Indians of the Juruá Valley, Acre State, Brazil. Toxicon [Internet]. 1996 Feb [cited 2015 Jan 1];34(2):225–36. Available from: http://www.ncbi.nlm.nih.gov/pubmed/8711756
8. Waldez F, Vogt RC. Ecological and epidemiological aspects of snakebites in riverside communities of the lower Purus River, Amazonas, Brazil. Acta Amaz [Internet]. Instituto Nacional de Pesquisas da Amazônia; 2009 Sep [cited 2015 Jan 2];39(3):681–92. Available from: http://www.scielo.br/scielo.php?script=sci_arttext&pid=S0044-

59672009000300025&lng=en&nrm=iso&tlng=pt

9. Feitosa ES, Sampaio V, Sachett J, Castro DB, Noronha M das DN, Lozano JLL, et al. Snakebites as a largely neglected problem in the Brazilian Amazon: highlights of the epidemiological trends in the States of Amazonas. Rev Soc Bras Med Trop. 2015;48(Suppl I):34–41.
10. Bernarde PS, Gomes JDO. Venomous snakes and ophidism in Cruzeiro do Sul, Alto Juruá, State of Acre, Brazil. Acta Amaz [Internet]. 2012;42(1):65–72. Available from: http://www.scielo.br/pdf/aa/v42n1/a08v42n1.pdf
11. Borges CC, Sadahiro M, Dos-Santos MC. Epidemiological and clinical aspects of snake accidentes in the municipalities of the State of Amazonas, Brazil. Rev Soc Bras Med Trop [Internet]. 1999;32(6):637–46. Available from: http://www.scielo.br/pdf/rsbmt/v32n6/0860.pdf
12. Nascimento SP. Epidemiological characteristics of snakebites in the State of Roraima, Brazil, 1992-1998. Cad Saude Publica [Internet]. 2000;16(1):271–6. Available from: http://www.scielosp.org/pdf/csp/v16n1/1589.pdf
13. Pardal PP de O, Souza SM, Monteiro MR de C da C, Fan HW, Cardoso JLC, França FOS, et al. Clinical trial of two antivenoms for the treatment of Bothrops and Lachesis bites in the north eastern Amazon region of Brazil. Trans R Soc Trop Med Hyg [Internet]. 2004 Jan [cited 2015 Jan 2];98(1):28–42. Available from: http://www.ncbi.nlm.nih.gov/pubmed/14702836
14. Sá-Neto RP, Dos-Santos M. Aspectos epidemiológicos dos acidentes ofídicos atendidos no Instituto de Medicina Tropical de Manaus (IMTM), 1986–92: estudo retrospectivo. Rev da Soc Bras Med Trop. 1995;28(supl I):171.
15. Moreno E, Queiroz-Andrade M, Lira-da-silva RM, Tavares-Neto J. Clinical and epidemiological characteristics of snakebites in Rio Branco, Acre. Rev Soc Bras Med Trop [Internet]. 2005;38(1):15–21. Available from: http://www.scielo.br/pdf/rsbmt/v38n1/22767.pdf
16. Pardal PP de O, Pardal JS de O, Gadelha MA da C, Rodrigues L da S, Feitosa DT, Prudente A lúcia da C, et al. ENVENOMATION BY Micrurus CORAL SNAKES IN THE BRAZILIAN AMAZON REGION: REPORT OF TWO CASES. Rev Inst Med Trop Sao Paulo [Internet]. 2010 [cited 2015 Mar 17];56(6):333–7. Available from:

http://www.scielo.br/pdf/rimtsp/v52n6/a09v52n6.pdf

17. Pardal PPDO, Silva CLQ Da, Hoshino SDSN, Pinheiro MDFR. SNAKEBITE BY THE RATTLESNAKE (Crotalus sp) IN PONTA DE PEDRA, MARAJÓ ISLAND, PARÁ- A CASE REPORT. Rev Para Med. 2007;21(3):69–73.
18. Saraiva MG, Oliveira DDS, Filho GMCF, Coutinho LASDA, Guerreiro JV. Epidemiological profile of snake bites in the State of Paraiba, Brazil, 2005 to 2010. Epidemiol e Serviços Saúde [Internet]. 2012;21(3):449–56. Available from: http://scielo.iec.pa.gov.br/scielo.php?script=sci_arttext&pid=S1679-49742012000300010&lng=en&nrm=iso&tlng=en
19. Feitosa EL, Sampaio VS, Salinas JL, Queiroz AM, da Silva IM, Gomes A a., et al. Older Age and Time to Medical Assistance Are Associated with Severity and Mortality of Snakebites in the Brazilian Amazon: A Case-Control Study. PLoS One [Internet]. 2015;10(7):e0132237. Available from: http://dx.plos.org/10.1371/journal.pone.0132237
20. Gold BS, Dart RC, Barish RA. Bites of Venomous Snakes. N Engl J Med. 2002;347(5):347–56.
21. Hui Wen F, Monteiro WM, Moura da Silva AM, Tambourgi D V., Mendonça da Silva I, Sampaio VS, et al. Snakebites and Scorpion Stings in the Brazilian Amazon: Identifying Research Priorities for a Largely Neglected Problem. PLoS Negl Trop Dis [Internet]. 2015;9(5):e0003701. Available from: http://dx.plos.org/10.1371/journal.pntd.0003701
22. Brasil. Fundação Nacional de Saúde. Manual de Diagnóstico e Tratamento de Acidentes por Animais Peçonhentos. Fundação N. Brasília: Fundação Nacional de Saúde; 2001. 120 p.
23. Calvete JJ, Sanz L, Pérez A, Borges A, Vargas AM, Lomonte B, et al. Snake population venomics and antivenomics of Bothrops atrox: Paedomorphism along its transamazonian dispersal and implications of geographic venom variability on snakebite management. J Proteomics [Internet]. 2011 Apr 1 [cited 2014 Dec 15];74(4):510–27. Available from: http://www.ncbi.nlm.nih.gov/pubmed/21278006
24. Bochner R, Fiszon JT, Machado C. A Profile of Snake Bites in Brazil, 2001 to 2012. J Clin Toxicol [Internet]. 2014 [cited 2014 Dec 19];04(03):194. Available from: http://omicsonline.org/open-access/a-profile-of-snake-bites-in-brazil-to-2161-0495.1000-194.php?aid=27518

25. Saravu K, Shastry A, Somavarapu V, Kumar R. Clinical profile, species-specific severity grading, and outcome determinants of snake envenomation: An Indian tertiary care hospital-based prospective study. Indian J Crit Care Med [Internet]. 2012 Oct [cited 2015 Mar 16];16(4):187. Available from: http://www.pubmedcentral.nih.gov/articlerender.fcgi?artid=3610449&tool=pmcentrez&rendertype=abstract

26. Sankar J, Nabeel R, Sankar MJ, Priyambada L, Mahadevan S. Factors affecting outcome in children with snake envenomation: a prospective observational study. Arch Dis Child [Internet]. 2013;98(8):596–601. Available from: http://www.ncbi.nlm.nih.gov/pubmed/23716133

27. Suchithra N, Pappachan JM, Sujathan P. Snakebite envenoming in Kerala, South India: clinical profile and factors involved in adverse outcomes. Emerg Med J [Internet]. 2008 Apr 1 [cited 2015 Mar 17];25(4):200–4. Available from: http://emj.bmj.com/content/25/4/200.abstract

28. Kalantri S, Singh A, Joshi R, Malamba S, Ho C, Ezoua J, et al. Clinical predictors of in-hospital mortality in patients with snake bite: a retrospective study from a rural hospital in central India. Trop Med Int Heal [Internet]. 2006 [cited 2015 Mar 17];2(1):22–30. Available from: file:///C:/Users/Jacqueline/Downloads/Kalantri_et_al-2006-Tropical_Medicine_&_International_Health.pdf

29. Dharod M V, Patil TB, Deshpande AS, Gulhane R V, Patil MB, Bansod Y. Clinical Predictors of Acute Kidney Injury Following Snake Bite Envenomation. N Am J Med Sci [Internet]. 2013 [cited 2015 Mar 17];5(10):594–9. Available from: http://www.najms.org/temp/NorthAmJMedSci510594-1938391_003218.pdf

30. Ogunfowokan O, Jacob DA, Livinus OL. Relationship between bite-to-hospital time and morbidity in victims of carpet viper bite in North-Central Nigeria. West Afr J Med [Internet]. Jan [cited 2015 Mar 17];30(5):348–53. Available from: http://www.ncbi.nlm.nih.gov/pubmed/22752823

31. Habib AG, Abubakar SB. Factors affecting snakebite mortality in north-eastern Nigeria. Int Health [Internet]. 2011 Mar [cited 2015 Mar 17];3(1):50–5. Available from: http://www.ncbi.nlm.nih.gov/pubmed/24038050

32. Kim JS, Yang JW, Kim MS, Han ST, Kim BR, Shin MS, et al. Coagulopathy in patients

who experience snakebite. Korean J Intern Med [Internet]. 2008 Jun [cited 2015 Mar 17];23(2):94–9. Available from: http://www.pubmedcentral.nih.gov/articlerender.fcgi?artid=2686970&tool=pmcentrez&rendertype=abstract

33. Souza ARB. Snakebite by Bothrops atrox (Lin. 1758) in the State of Amazonas - Brazil: Study of 212 cases with identified snake. Rev Patol Trop [Internet]. 2002 [cited 2015 Mar 17];31(2):267–8. Available from: http://www.revistas.ufg.br/index.php/iptsp/article/viewFile/14573/9140

34. Ministerio da Saúde. Secretaria de Vigilancia em Saúde. Departamento de Vigilancia Epidemiologica. Guia de vigilância em saúde. Ministério. Brasilia: Ministério da Saúde; 2014. 812 p.

35. Lima ACSF, Campos CEC, Ribeiro JR. Epidemiological profile of snake poisoning accidents in the State of Amapá. Rev Soc Bras Med Trop [Internet]. SBMT; 2009 Jun [cited 2015 Jan 4];42(3):329–35. Available from: http://www.scielo.br/scielo.php?script=sci_arttext&pid=S0037-86822009000300017&lng=en&nrm=iso&tlng=pt

36. Guércio RAP, Shevchenko A, Shevchenko A, López-Lozano JL, Paba J, Sousa M V, et al. Ontogenetic variations in the venom proteome of the Amazonian snake Bothrops atrox. Proteome Sci [Internet]. 2006 Jan [cited 2014 Nov 18];4(1):11. Available from: http://www.proteomesci.com/content/4/1/11

37. Saboriâo P, Gonza M, Cambronero M. Accidente Ofídico en Niños en Costa Rica: Epidemiología Y Deteccíon de Factores de Riesgo en el Desarrollo de Absceso Y Necrosis. Toxicon. 1998;36(2):359–66.

38. Michael GC, Thacher TD, Shehu MIL. The effect of pre-hospital care for venomous snake bite on outcome in Nigeria. Trans R Soc Trop Med Hyg [Internet]. Royal Society of Tropical Medicine and Hygiene; 2011 Feb [cited 2014 Dec 19];105(2):95–101. Available from: http://www.ncbi.nlm.nih.gov/pubmed/21035155

39. Pinho FMO, Zanetta DMT, Burdmann EA. Acute renal failure after Crotalus durissus snakebite: a prospective survey on 100 patients. Kidney Int [Internet]. 2005 Feb [cited 2015 Mar 17];67(2):659–67. Available from: http://www.ncbi.nlm.nih.gov/pubmed/15673314

40. Rodrigues Sgrignolli L, Florido Mendes GE, Carlos CP, Burdmann E a. Acute kidney injury caused by bothrops snake venom. Nephron Clin Pract [Internet]. 2011 Jan [cited 2014 Dec 19];119(2):c131–7. Available from: http://www.ncbi.nlm.nih.gov/pubmed/21757950

41. Albuquerque PLMM, Silva GB, Jacinto CN, Lima JB, Lima CB, Amaral YS, et al. Acute kidney injury after snakebite accident treated in a Brazilian tertiary care centre. Nephrology [Internet]. 2014 Dec [cited 2015 Mar 16];19(12):764–70. Available from: http://www.ncbi.nlm.nih.gov/pubmed/25123203

42. Otero R, Gutiérrez J, Beatriz Mesa M, Duque E, Rodríguez O, Luis Arango J, et al. Complications of Bothrops, Porthidium, and Bothriechis snakebites in Colombia. A clinical and epidemiological study of 39 cases attended in a university hospital. Toxicon [Internet]. 2002 Aug [cited 2015 Mar 17];40(8):1107–14. Available from: http://www.ncbi.nlm.nih.gov/pubmed/12165312

43. Alkaabi JM, Al Neyadi M, Al Darei F, Al Mazrooei M, Al Yazedi J, Abdulle AM. Terrestrial snakebites in the South East of the Arabian Peninsula: patient characteristics, clinical presentations, and management. PLoS One [Internet]. 2011 Jan [cited 2014 Dec 19];6(9):e24637. Available from: http://www.pubmedcentral.nih.gov/articlerender.fcgi?artid=3171447&tool=pmcentrez&rendertype=abstract

44. Chen C-M, Wu K-G, Chen C-J, Wang C-M. Bacterial infection in association with snakebite: a 10-year experience in a northern Taiwan medical center. J Microbiol Immunol Infect [Internet]. Elsevier Taiwan LLC; 2011 Dec [cited 2014 Dec 19];44(6):456–60. Available from: http://www.ncbi.nlm.nih.gov/pubmed/21700517

45. Ozay G, Bosnak M, Ece A, Davutoglu M, Dikici B, Gurkan F, et al. Clinical characteristics of children with snakebite poisoning and management of complications in the pediatric intensive care unit. Pediatr Int [Internet]. 2005 Dec [cited 2015 Mar 17];47(6):669–75. Available from: http://www.ncbi.nlm.nih.gov/pubmed/16354222

46. David S, Matathia S, Christopher S. Mortality predictors of snake bite envenomation in southern India-A ten-year retrospective audit of 533 patients. J Med Toxicol [Internet]. 2012 Jun [cited 2015 Mar 17];8(2):118–23. Available from: http://www.pubmedcentral.nih.gov/articlerender.fcgi?artid=3550238&tool=pmcentrez&re

ndertype=abstract

47. Ribeiro LA, Jorge MT. Acidente por serpentes do gênero Bothrops: série de 3.139 casos. Rev Soc Bras Med Trop. 1997;30(6):475–80.

48. Queiroz SJ de. IDENTIFICAÇÃO DA ATIVIDADE ANTIMICROBIANA NO VENENO DA SERPENTE Bothrops moojeni EM BACTÉRIAS GRAM NEGATIVAS. PONTIFÍCIA UNIVERSIDADE CATÓLICA DE GOIÁS; 2010.

49. Williams SS, Wijesinghe C a, Jayamanne SF, Buckley N a, Dawson AH, Lalloo DG, et al. Delayed psychological morbidity associated with snakebite envenoming. PLoS Negl Trop Dis [Internet]. 2011 Aug [cited 2014 Dec 19];5(8):e1255. Available from: http://www.pubmedcentral.nih.gov/articlerender.fcgi?artid=3149015&tool=pmcentrez&rendertype=abstract

50. Vaiyapuri S, Vaiyapuri R, Ashokan R, Ramasamy K, Nattamaisundar K, Jeyaraj A, et al. Snakebite and its socio-economic impact on the rural population of Tamil Nadu, India. PLoS One [Internet]. 2013 Jan [cited 2014 Dec 19];8(11):e80090. Available from: http://www.pubmedcentral.nih.gov/articlerender.fcgi?artid=3836953&tool=pmcentrez&rendertype=abstract

51. Bochner R, Struchiner CJ. Exploratory analysis of environmental and socioeconomic factors related to snakebite incidence in Rio de Janeiro from 1990 to 1996. Cad Saúde Pública. 2004;20(4):976–85.

52. Fonseca M, Moreira W, Cunha K, Ribeiro A, Almeida M. ORAL MICROBIOTA OF BRAZILIAN CAPTIVE SNAKES. J Venom Anim Toxins incl Trop Dis. 2009;15(1):54–60.

53. Paula RCMF. PERFIL EPIDEMIOLÓGICO DOS CASOS DE ACIDENTES OFÍDICOS ATENDIDOS NO HOSPITAL DE DOENÇAS TROPICAIS DE ARAGUAÍNA-TO (TRIÊNIO 2007-2009). Dissertação. Instituto de Pesquisas Energéticas e Nucleares; 2010.

54. Jorge MT, Mendonça JS de, Ribeiro LA, Silva MLR, Kusano EJU, Cordeiro CL dos S. Flora bacteriana da cavidade oral, presas de Bothrops jararaca: possível fonte de infecção no local da picada. Rev Inst Med Trop São Paulo. São Paulo: Rev. Inst. Med. trop. São Paulo; 1990;32(1):6–10.

55. Cheng AC, Currie BJ. Venomous Snakebites Worldwide with a Focus on the Australia-Pacific Region: Current Management and Controversies. J Intensive Care Med

[Internet]. 2004;19(5):259–69. Available from: http://jic.sagepub.com/cgi/doi/10.1177/0885066604265799

56. Nicoleti AF. Comparação dos acidentes causados por Bothropoides jararaca (Serpentes: Viperidae) com e sem envenenamento atendidos no Hospital Vital Brazil do Instituto Butantan. Faculdade de Medicina da Universidade de São Paulo; 2010.

57. Garg A, Sujatha S, Garg J, Acharya NS, Parija SC. Wound infections secondary to snakebite. J Infect Dev Ctries. 2009;3(3):221–3.

58. Jorge MT, Malaque C, Ribeiro L a, Fan HW, Cardoso JLC, Nishioka S a, et al. Failure of chloramphenicol prophylaxis to reduce the frequency of abscess formation as a complication of envenoming by Bothrops snakes in Brazil: a double-blind randomized controlled trial. Trans R Soc Trop Med Hyg [Internet]. 2004 Sep [cited 2014 Dec 19];98(9):529–34. Available from: http://www.ncbi.nlm.nih.gov/pubmed/15251401

59. Andrade JG de, Pinto RNL, Andrade ALSS de, Martelli CMT, Zicker F. ESTUDO BACTERIOLÓGICO DE ABSCESSOS CAUSADOS POR PICADA DE SERPENTES DO GÊNERO BOTHROPS. Rev Inst Med Trop São Paulo. 1989;31(6):363–7.

60. Oliveira RB De, Ribeiro LA, Jorge MT. Risk factors associated with coagulation abnormalities in Bothrops envenoming. Rev Soc Bras Med Trop. 2003;36(6):657–63.

61. Ribeiro LA, Gadia R, Jorge MT. Comparison between the epidemiology of accidents and the clinical features of envenoming by snakes of the genus Bot. Rev Soc Bras Med Trop. 2008;41(1):46–9.

62. Bucaretchi F, Herrera SRF, Hyslop S, Bacarat ECE, Vieira RJ. SNAKEBITES BY Bothr ops spp IN CHILDREN IN CAMPINAS, SÃO PAULO, BRAZIL. Rev Inst Med Trop São Paulo. 2001;43(6):329–33.

63. Goldstein EJ. Bite wounds and infection. Clin Infect Dis [Internet]. 1992 Mar [cited 2015 Jan 7];14(3):633–40. Available from: http://www.ncbi.nlm.nih.gov/pubmed/1562653

64. Goldstein EJC, Citron DM, Gonzalez H, Russell FE, Finegold SM. Bacteriology of Rattlesnake Venom and Implications for Therapy. J Infect Dis [Internet]. 1979;140(5):818–21. Available from: http://jid.oxfordjournals.org/lookup/doi/10.1093/infdis/140.5.818

65. Bastos HM, Lopes LFL, Gattamorta MA, Matushima ER. Prevalence of enterobacteria in Bothrops jararaca in São Paulo State: microbiological survey and antimicrobial

resistance standards. Acta Sci Biol Sci [Internet]. 2008 Oct 9 [cited 2014 Dec 19];30(3):321–6. Available from: http://periodicos.uem.br/ojs/index.php/ActaSciBiolSci/article/view/536

66. JORGE MT, MENDONÇA JS, RIBEIRO LA, CARDOSO JLC, SILVA ML. Bacilos Gram-negativos aeróbios em abscessos por acidente botrópico. Rev Soc Bras Med Trop. 1987;20(supl.):55.

67. Jorge MT, Ribeiro LA, Da Silva MLR, Kusano EJU, Mendonça JS de. Microbiological studies of abscesses complicating Bothrops snakebite in humans: A prospective study. Toxicon. 1994;32(6):743–8.

68. Gutiérrez JM, Rucavado A, Chaves F, Díaz C, Escalante T. Experimental pathology of local tissue damage induced by Bothrops asper snake venom. Toxicon [Internet]. 2009 Dec 1 [cited 2014 Dec 15];54(7):958–75. Available from: http://www.ncbi.nlm.nih.gov/pubmed/19303033

69. Albuquerque PLMM, Jacinto CN, Silva Junior GB, Lima JB, Veras M do SB, Daher EF. Acute kidney injury caused by Crotalus and Bothrops snake venom: a review of epidemiology, clinical manifestations and treatment. Rev Inst Med Trop Sao Paulo [Internet]. 2013 Jan [cited 2015 Mar 17];55(5):295–301. Available from: http://www.pubmedcentral.nih.gov/articlerender.fcgi?artid=4105065&tool=pmcentrez&rendertype=abstract

70. Stevens DL, Bisno AL, Chambers HF, Dellinger EP, Goldstein EJC, Gorbach SL, et al. Practice guidelines for the diagnosis and management of skin and soft tissue infections: 2014 update by the infectious diseases society of America. Clin Infect Dis [Internet]. 2014 Jul 15 [cited 2014 Dec 15];59(2):1–43. Available from: http://www.ncbi.nlm.nih.gov/pubmed/24947530

71. Stevens DL, Bisno AL, Chambers HF, Everett ED, Dellinger P, Goldstein EJC, et al. Practice Guidelines for the Diagnosis and Management of Skin and Soft-Tissue Infections. Clin Infect Dis [Internet]. 2005;41:1373–406. Available from: http://cid.oxfordjournals.org/

72. Tagwireyi DD, Ball DE, Nhachi CF. Routine prophylactic antibiotic use in the management of snakebite. BMC Clin Pharmacol [Internet]. 2001;1(4):7. Available from: http://www.pubmedcentral.nih.gov/articlerender.fcgi?artid=59881&tool=pmcentrez&rend

ertype=abstract

73. Kerrigan K, Mertz B, Nelson S, Dye J. Antibiotic prophylaxis for pit viper envenomation: prospective, controlled trial. World J Surg [Internet]. 1997;21(4):369–73. Available from: papers2://publication/uuid/2CEFE28B-F057-4003-94EB-4C292221FF19
74. Brakenbury PH, Muwanga C. A comparative double blind study of amoxycillin/clavulanate vs placebo in the prevention of infection after animal bites. Arch Emerg Med. 1989;6:251–6.
75. Scottish Intercollegiate Guidelines Network. SIGN 104 • Antibiotic prophylaxis in surgery. NHS - SIGN Clin Guidel. 2014;104:1–67.
76. Bratzler DW, Dellinger EP, Olsen KM, Perl TM, Auwaerter PG, Bolon MK, et al. Clinical practice guidelines for antimicrobial prophylaxis in surgery. Am J Heal Pharm. 2013;70:195–283.
77. Classen DC, Evans RS, Pestotnik SL, Horn SD, Menlove RL, Burke JP. The timing of prophylactic adminisration of antibiotics and the risk of surgival-wound infection. N Engl J Med. 1992;326(5):281–6.
78. Nishioka SA, Silveira PVP. Philodryas patagoniensis bite and local envenoming. Revista do Instituto de Medicina Tropical de Sao Paulo. 1994. p. 279–81.
79. Liu P, Shi Z, Lin C, Huang J, Liu J, Chan K, et al. Shewanella infection of snake bites: a twelve-year retrospective study. Clinics [Internet]. 2012 May 15 [cited 2014 Dec 19];67(5):431–5. Available from: http://www.ncbi.nlm.nih.gov/pmc/articles/PMC3351262/
80. Bisno AL, Stevens DL. Streptococcal Infections of Skin and Soft Tissues. N Engl J Med. 1996;334(4):240–5.
81. Cook RJ, Sackett DL. The number needed to treat: a clinically useful measure of treatment effect. BMJ. 1995;310:452–4.
82. Illumina. 16S Metagenomic Sequencing Library [Internet]. Illumina.com. 2013. 1-28 p. Available from: http://support.illumina.com/content/dam/illumina-support/documents/documentation/chemistry_documentation/16s/16s-metagenomic-library-prep-guide-15044223-b.pdf

APÊNDICE A – TABELA RANDOMIZADA

Tabela de Randomização Grupo 1 = Placebo Grupo 2 = Antibioticoterapia

N° Paciente	Grupo	N° Prontuário
1	2	
2	2	
3	2	
4	1	
5	1	
6	1	
7	2	
8	2	
9	2	
10	1	
11	2	
12	1	
13	2	
14	2	
15	2	
16	2	
17	1	
18	2	
19	2	
20	2	
21	2	
22	1	
23	1	
24	1	
25	1	
26	2	
27	1	
28	1	
29	2	
30	1	

N° Paciente	Grupo	N° Prontuário
31	2	
32	2	
33	1	
34	2	
35	2	
36	2	
37	1	
38	2	
39	2	
40	2	
41	1	
42	1	
43	2	
44	1	
45	1	
46	2	
47	2	
48	2	
49	2	
50	2	
51	2	
52	1	
53	1	
54	1	
55	1	
56	1	
57	2	
58	1	
59	2	
60	2	

N° Paciente	Grupo	N° Prontuário
61	2	
62	2	
63	1	
64	2	
65	1	
66	1	
67	1	
68	2	
69	1	
70	2	
71	1	
72	2	
73	2	
74	1	
75	1	
76	2	
77	2	
78	2	
79	1	
80	2	
81	1	
82	1	
83	1	
84	2	
85	2	
86	1	
87	2	
88	1	
89	2	
90	1	

N° Paciente	Grupo	N° Prontuário
91	2	
92	2	
93	2	
94	1	
95	2	
96	2	
97	2	
98	1	
99	1	
100	1	
101	2	
102	2	
103	1	
104	1	
105	1	
106	2	
107	1	
108	2	
109	2	
110	2	
111	1	
112	2	
113	1	
114	2	
115	2	
116	2	
117	2	
118	1	
119	1	
120	2	
121	1	

N° Paciente	Grupo	N° Prontuário
122	2	
123	1	
124	1	
125	1	
126	1	
127	2	
128	2	
129	2	
130	1	
131	2	
132	2	
133	2	
134	2	
135	2	
136	1	
137	1	
138	2	
139	1	
140	1	
141	1	
142	2	
143	1	
144	2	
145	1	
146	2	
147	2	
148	1	
149	1	
150	1	
151	1	
152	2	

N° Paciente	Grupo	N° Prontuário
153	1	
154	1	
155	2	
156	2	
157	2	
158	1	
159	1	
160	1	
161	1	
162	2	
163	1	
164	2	
165	1	
166	2	
167	1	
168	2	
169	2	
170	2	
171	2	
172	1	
173	2	
174	1	
175	2	
176	2	
177	2	
178	2	
179	1	
180	1	
181	2	
182	2	
183	2	

APÊNDICE B – PROTOCOLO DE PESQUISA

1

Anexo:

Formulário de Registro Clínico - CRF

"Ensaio clínico randomizado para avaliação da superioridade de Amoxicilina/Ácido Clavulânico versus placebo na prevenção de infecção bacteriana secundária em acidentes ofídicos na Amazônia Brasileira"

Prontuário:

Nome:

Cod. Estudo:

Pesquisadores Responsáveis: Jacqueline A. G. Sachett **Contato**: (92) 8151-8086
Iran Mendonça da Silva **Contato**: (92) 9322-3775

	Critérios de Não Inclusão	
1.	Tempo entre o acidente e o atendimento **maior** que 24 horas ou não saber informar a hora do acidente	○ Sim ○ **Não**
2.	Tratamento prévio com antibioticoterapia nos últimos 30 dias	○ Sim ○ **Não**
3.	Possui abscesso ou infecção claramente definida	○ Sim ○ **Não**
4.	Alérgico ao Clavulin BD	○ Sim ○ **Não**
5.	Está grávida ou suspeita de gravidez (fazer teste específico)	○ Sim ○ **Não**
6.	Utilizou soro antiofídico em outra unidade de saúde	○ Sim ○ **Não**
8.	Incapacidade ou falta de vontade de assinar o consentimento informado (paciente e/ou pais / representante legal)	○ Sim ○ **Não**
7.	Indisponibilidade antecipada para acompanhamento/retorno no sétimo dia após o acidente	○ Sim ○ **Não**
8.	Qualquer condição descompensada ou não controlada como tuberculose ativa, doença maligna, malária grave, HIV, hanseníase, doença fúngica sistêmica (histoplasmose, paracoccidioidomicose), hepatite B, hepatite C ou qualquer outra doença infecto-contagiosa;	○ Sim ○ **Não**

PARA SELEÇÃO DO INDIVÍDUO, TODAS AS RESPOSTAS DEVEM SER **"NÃO"**

Randomização

Grupo 1: (   ) Placebo

Grupo 2: (   ) Clavulin BD - 25/3,6mg/kg/dia – 12x12 horas - Crianças e adolescentes

875/125 mg – 12x12 horas - Adultos

Hora da Administração do Antibiótico:_____:_____

Assinatura e carimbo: ____

- UTILIZAR TABELA DE RANDOMIZAÇÃO

1. Dados do paciente

1.1 Contato telefônico: ( ) ______________ ( ) _________________
1.2 Endereço: ____________________________________n° ____ Bairro: ___________
1.3 Município: _________________

1.4 Sexo: ☐ 1-Masculino 2-Feminino

1.5 Data de nascimento ____/____/____ **1.6** Idade: ☐☐

2. Dados do acidente ofídico

2.1 Procedência: ☐ 1-Manaus 2-Interior Qual? ____________________________

2.2 Data do Acidente: ____/____/____ **2.3** Hora Aproximada do Acidente:_____:_____

2.4 Data da internação na FMT-HVD:____/____/____ **2.5** Hora da Admissão:_____:_____

2.6 Data da saída na FMT-HVD: ____/____/____

2.7 Motivo: ☐ 1-Alta 2-Óbito 3-Transferência

2.8 Zona de Ocorrência: ☐ 1-Rural 2-Urbana 3-Periurbana 9-Ignorado

2.9 Local da picada: ☐ 1-Cabeça 2-Braço 3-Perna 4-Tronco 5-Pescoço 6-Mão 7-Coxa 8-Antebraço 9-Pé 10-Outro (Espec.):_________________

2.10 Lado do corpo afetado: ☐ 1-Direito 2-Esquerdo 3-Central

2.11 Andou ou caminhou após acidente: ☐ 1-Não 2-Sim. Quanto tempo? ____ min.

2.12 Acidente relacionado ao trabalho: ☐ 1-Não 2-Sim

2.13 Houve acidente anterior: ☐ 1-Não 2-Sim. Quantos?_____
Data do último: ____/_____ (mês/ano)

Assinatura e Carimbo: ____

3. Soroterapia – Classificação do Acidente ofídico

3.1 Tipo do acidente ☐ 1-Botrópico 2-Laquético 3-Não identificado

3.2 Classificação do acidente (**vide tabela abaixo**): ☐ 1-Leve 2-Moderado 3-Grave

Classificação do Acidente e Soroterapia (*antes de iniciar soroterapia coletar sangue conforme POP – coleta sanguínea):

Botrópico	
**Leve**: quadro local discreto (edema local de até 2 segmentos), sangramento em pele ou mucosas; pode haver apenas distúrbio na coagulação.<br>**Soro**: Antibotrópico (SAB)	2 a 4 ampolas
**Moderado**: edema (edema local de 3 a 4 segmentos) e equimose evidentes, sangramento sem comprometimento do estado geral; pode haver distúrbio na coagulação.<br>**Soro**: Antibotrópico (SAB)	5 a 8 ampolas
**Grave**: alterações locais intensas (edema local de 5 segmentos), hemorragia grave, hipotensão, anúria.<br>**Soro**: Antibotrópico (SAB)	12 ampolas

Laquético	
**Moderado**: quadro local presente (Idem Bothropico), pode haver sangramentos, manifestações vagais leves (PA > 80x40mmHg e Pulso acima de 50bpm)<br>**Soro**: Antibotrópico-laquético (SABL)	10 ampolas
**Grave**: quadro local intenso (Idem Bothropico), hemorragia intensa, com manifestações vagais (PA < 80x40mHg e Pulso abaixo de 50bpm)<br>**Soro**: Antibotrópico-laquético (SABL)	20 ampolas

3.3 Hora da Soroterapia: _____:_____

3.4 Reação Adversa à Soroterapia (até 24 horas após infusão): 1-Não 2-Sim

☐ taquicardia ☐ taquipneia ☐ dispneia ☐ urticária ☐ náuseas

☐ vômitos ☐ hipotensão ☐ prurido ☐ choque ☐ broncoespasmo

☐ rouquidão e estridor laríngeo ☐ reação anafilática

☐ outra: ____

3.5 Hora da Reação Adversa: _____:_____

Assinatura e Carimbo: ____

4. Histórico – Dia 1 (0-12 horas)

4.1 Houve medicamentos orais/injetáveis anteriores a internação:

☐ 1-Não 2-Sim (Espec.):______________________________

4.2 Houve uso de produtos no local do acidente:

☐ 1-Não 2-Sim (Espec.):______________________________

4.3 Condutas adotadas após o acidente 1-Não 2-Sim

☐ Torniquete/garrote ☐ Sucção Labial ☐ Outro: ____________________

5. Dados clínicos do paciente

5.1 Data da avaliação: |__|__||__|__||__|__| **5.2 Hora**:____:____

5.3 Pressão arterial |__|__|__|/|__|__|__| mm Hg **5.4 Pulso** |__|__|__| por min

5.5 Peso corporal |__|__| kg **5.6. Temp.** |__|__|.|__|°C **5.7 Freq. Resp.** |__|__| rpm

5.8 Doenças, Tratamentos ou Queixas concomitantes 1-Não 2-Sim

☐ Doenças Hematológicas ☐ Uso de anticoncepcional

☐ Uso de anticoagulante ☐ Outro: ____________________

5.9 Manifestações Locais: 1-Não 2-Sim

5.9.1 **Sangramento** ☐ sangramento em pele ou mucosas

☐ equimose evidentes

☐ sangramento sem comprometimento do estado geral

5.9.2 **Edema** ☐ Leve - edema local de até 2 segmentos

☐ Moderado - edema local de 3 a 4 segmentos

☐ Grave - edema local de 5 segmentos

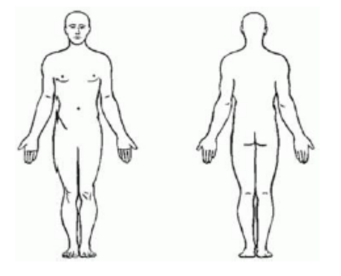

- Circunferência da região acometida (Centímetros)

Lado direito: _____cm Lado esquerdo: ____cm região ímpar: ____cm

Extensão do edema: _____cm*

* marcar na figura o edema tanto no sentido distal quanto proximal do local da picada (marcar com P - local da picada)

5.9.3 **Linfonodos** ☐ ausente ☐ presente Tamanho (cm): ________

Consistência ☐ elástica ☐ pétrea ☐ amolecida

Sensibilidade ☐ doloroso ☐ indolor

Mobilidade ☐ aderido a planos profundos ☐ não aderido (móvel)

5.9.4 **Dor - classificação numérica na escala de 0 - 10**

Valor*: ☐ Ausente: 0 Leve: 1-3 Moderado: 4-7 Grave: 8-10

* Antes do uso de analgésico

5.9.5 **Tonalidade Perilesional:** 1-Não 2-Sim

☐ normal ☐ violácia ☐ vermelhidão ☐ necrótica (enegrecida)

5.9.6 **Características da Pele:** 1-Não 2-Sim

☐ equimose ☐ bolhas ☐ secreção serosa ☐ somente a marca da presa

5.9.7 Temperatura: Área acometida _____°C 5.9.8 Área contralateral _____°C

5.10 Manifestações Sistêmicas: 1-Não 2-Sim, marcar um X

☐ Anúria ☐ Cefaleia ☐ Choque ☐ Cólicas abdominiais ☐ Convulsão

☐ Diarreia ☐ Enterorragia(melena e hematoquesia) ☐ Epistaxe

☐ Equimose ☐ Gengivorragia ☐ Hematêmese ☐ Hematúria

☐ Hemoptise ☐ Hemorragia conjuntival ☐ Hipermenorragia ☐ Náuseas

☐ Oligúria ☐ Otorragia ☐ Petéquias ☐ Sudorese ☐ Vômito

☐ Outras:____

Assinatura e Carimbo: ____

6. Dados da lesão do acidente ofídico

6.1 Infecção Secundária ao Acidente Ofídico: ☐ 1-Não 2-Sim

6.2 Data do diagnóstico: ____/____/____ **6.3 Hora**: ____:____

6.4 Manifestações Locais: 1-Não 2-Sim

☐ celulite ☐ abscesso ☐ Outro (Espec.): ______________________________

6.5 Antibioticoterapia: ____________________ **Posologia:** ______________

<u>Fotodocumentação</u>: Favor tirar uma foto da lesão antes da retirada da amostra para exame.

Assinatura e Carimbo:____

7. Tratamento/medicação concomitante (até o final do tratamento)

7.1 Data: |__|__||__|__||__|__|

7.2 Medicações concomitantes? ☐ 1-Não 2-Sim – Especifique:

Marca / Nome genérico	Via/formul.	Regime	Indicação	Data de início	Data de término
....	....	....	....	|__|__||__|__||__|__|	○ |__|__||__|__||__|__|
....	....	....	....	|__|__||__|__||__|__|	○ |__|__||__|__||__|__|
....	....	....	....	|__|__||__|__||__|__|	○ |__|__||__|__||__|__|
....	....	....	....	|__|__||__|__||__|__|	○ |__|__||__|__||__|__|

Assinatura e Carimbo:____

8. Dados da serpente

8.1 Trouxe a serpente: ☐ 1-Não 2-Sim

INFORMAÇÕES ABAIXO SERÃO INFORMADAS PELA GERÊNCIA DE ANIMAIS PEÇONHENTOS

8.2 Serpente envolvida: ☐ 1-*Bothrops* 2-*Lachesis* 9-Ignorado
3-Outro (Espec.): _____________

8.3 Sexo da serpente: ☐ 1-Masculino 2-Feminino

8.4 Idade da serpente: ______________ **8.5 Tamanho da serpente**: _______cm.

9. Dadros clínicos do paciente - Dia 2 (12-24 horas)

9.1 Data da avaliação: |__|__||__|__||__|__| **9.2 Hora**:____:____

9.3 Pressão arterial |__|__|__|/|__|__|__| mm Hg **9.4 Pulso** |__|__|__| por min

9.5 Temp. |__|__|.|__| °C **9.6 Freq. Resp.** |__|__| rpm

9.7 Queixas concomitantes

☐ 1-Não 2-Sim (Espec.):____

9.8 Manifestações Locais: 1-Não 2-Sim

9.8.1 **Sangramento** ☐ sangramento em pele ou mucosas

☐ equimose evidentes

☐ sangramento sem comprometimento do estado geral

9.8.2 **Edema** ☐ Leve - edema local de até 2 segmentos

☐ Moderado - edema local de 3 a 4 segmentos

☐ Grave - edema local de 5 segmentos

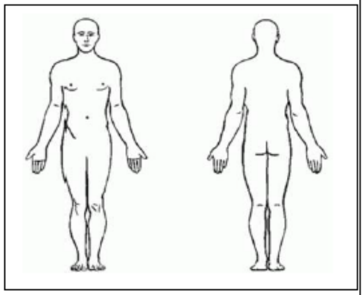

- Circunferência da região acometida (Centímetros)

Lado direito: ______cm Lado esquerdo: _____cm região ímpar: _____cm

Extensão do edema: ______cm*

* marcar na figura o edema tanto no sentido distal quanto proximal do local da picada (marcar com P - local da picada)

9.8.3 **Linfonodos** ☐ ausente ☐ presente Tamanho (cm): ________

Consistência ☐ elástica ☐ pétrea ☐ amolecida

Sensibilidade ☐ doloroso ☐ indolor

Mobilidade ☐ aderido a planos profundos ☐ não aderido (móvel)

9.8.4 **Dor - classificação numérica na escala de 0 - 10**

Valor*: ☐ Ausente: 0 Leve: 1-3 Moderado: 4-7 Grave: 8-10

* Após uso de analgésico

9.8.5 **Tonalidade Perilesional:** 1-Não 2-Sim

☐ normal ☐ violácia ☐ vermelhidão ☐ necrótica (enegrecida)

9.8.6 **Características da Pele:** 1-Não 2-Sim

☐ equimose ☐ bolhas ☐ secreção serosa ☐ somente a marca da presa

9.8.7 Temperatura: Área acometida _____°C 9.8.8 Área contralateral _____°C

9.9 Manifestações Sistêmicas: 1-Não 2-Sim, marcar um X

☐ Anúria ☐ Cefaleia ☐ Choque ☐ Cólicas abdominiais ☐ Convulsão

☐ Diarreia ☐ Enterorragia(melena e hematoquesia) ☐ Epistaxe

☐ Equimose ☐ Gengivorragia ☐ Hematêmese ☐ Hematúria

☐ Hemoptise ☐ Hemorragia conjuntival ☐ Hipermenorragia ☐ Náuseas

☐ Oligúria ☐ Otorragia ☐ Petéquias ☐ Sudorese ☐ Vômito

☐ Outras:____

Assinatura e Carimbo: ____

10. Dados da lesão do acidente ofídico

10.1 Infecção Secundária ao Acidente Ofídico: ☐ 1-Não 2-Sim

10.2 Data do diagnóstico: ____/____/____ **10.3 Hora**: ____:____

10.4 Manifestações Locais: 1-Não 2-Sim

☐ celulite ☐ abscesso ☐ Outro (Espec.): ______________________________

10.5 Antibioticoterapia: ____________________ **Posologia:** _______________

***Fotodocumentação**: Favor tirar uma foto da lesão antes da retirada da amostra para exame.*

Assinatura e Carimbo:____

11. Dados clínicos do paciente - Dia 3 (24-48 horas)

11.1 Data da avaliação: |__|__||__|__||__|__| **11.2 Hora**:____:____

11.3 Pressão arterial |__|__|__|/|__|__|__| mm Hg **11.4 Pulso** |__|__|__| por min

11.5 Temp. |__|__|.|__|°C **11.6 Freq. Resp.** |__|__|rpm

11.7 Queixas concomitantes

☐ 1-Não 2-Sim (Espec.):____

11.8 Manifestações Locais: 1-Não 2-Sim

11.8.1 **Sangramento** ☐ sangramento em pele ou mucosas

☐ equimose evidentes

☐ sangramento sem comprometimento do estado geral

11.8.2 **Edema** ☐ Leve - edema local de até 2 segmentos

☐ Moderado - edema local de 3 a 4 segmentos

☐ Grave - edema local de 5 segmentos

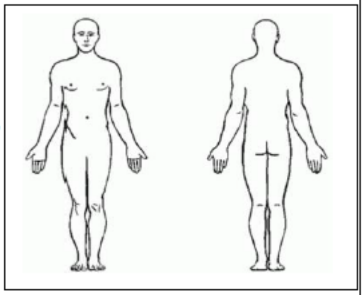

- Circunferência da região acometida (Centímetros)

Lado direito: _____cm Lado esquerdo: ____cm região ímpar: ____cm

Extensão do edema: _____cm*

* marcar na figura o edema tanto no sentido distal quanto proximal do local da picada (marcar com P - local da picada)

11.8.3 **Linfonodos** ☐ ausente ☐ presente Tamanho (cm): _______

Consistência ☐ elástica ☐ pétrea ☐ amolecida

Sensibilidade ☐ doloroso ☐ indolor

Mobilidade ☐ aderido a planos profundos ☐ não aderido (móvel)

11.8.4 **Dor - classificação numérica na escala de 0 - 10**

Valor*: ☐ Ausente: 0 Leve: 1-3 Moderado: 4-7 Grave: 8-10

* Após uso de analgésico

11.8.5 **Tonalidade Perilesional:** 1-Não 2-Sim

☐ normal ☐ violácia ☐ vermelhidão ☐ necrótica (enegrecida)

11.8.6 **Características da Pele:** 1-Não 2-Sim

☐ equimose ☐ bolhas ☐ secreção serosa ☐ somente a marca da presa

11.8.7 Temperatura: Área acometida _____°C 11.8.8 Área contralateral _____°C

11.9 Manifestações Sistêmicas: 1-Não 2-Sim, marcar um X

☐ Anúria ☐ Cefaleia ☐ Choque ☐ Cólicas abdominiais ☐ Convulsão

☐ Diarreia ☐ Enterorragia(melena e hematoquesia) ☐ Epistaxe

☐ Equimose ☐ Gengivorragia ☐ Hematêmese ☐ Hematúria

☐ Hemoptise ☐ Hemorragia conjuntival ☐ Hipermenorragia ☐ Náuseas

☐ Oligúria ☐ Otorragia ☐ Petéquias ☐ Sudorese ☐ Vômito

☐ Outras:____

Assinatura e Carimbo: ____

12. Dados da lesão do acidente ofídico

12.1 Infecção Secundária ao Acidente Ofídico: ☐ 1-Não 2-Sim

12.2 Data do diagnóstico: ____/____/____ **12.3 Hora:** ____:____

12.4 Manifestações Locais: 1-Não 2-Sim

☐ celulite ☐ abscesso ☐ Outro (Espec.): ________________________________

12.5 Antibioticoterapia: ______________________ **Posologia:** ________________

***Fotodocumentação**: Favor tirar uma foto da lesão antes da retirada da amostra para exame.*

Assinatura e Carimbo:____

13. Dados clínicos do paciente - Dia 4 (48-72 horas)

13.1 Data da avaliação: |__|__||__|__||__|__| **13.2 Hora**:____:____

13.3 Pressão arterial |__|__|__|/|__|__|__| mm Hg **13.4 Pulso** |__|__|__| por min

13.5 Temp. |__|__|.|__| °C **13.6 Freq. Resp.** |__|__| rpm

13.7 Queixas concomitantes

☐ 1-Não 2-Sim (Espec.):____

13.8 Manifestações Locais: 1-Não 2-Sim

13.8.1 **Sangramento** ☐ sangramento em pele ou mucosas

☐ equimose evidentes

☐ sangramento sem comprometimento do estado geral

13.8.2 **Edema** ☐ Leve - edema local de até 2 segmentos

☐ Moderado - edema local de 3 a 4 segmentos

☐ Grave - edema local de 5 segmentos

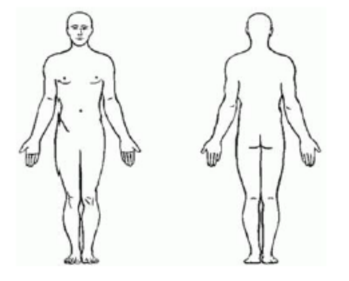

- Circunferência da região acometida (Centímetros)

Lado direito: _____cm Lado esquerdo: ____cm região ímpar: ____cm

Extensão do edema: _____cm*

* marcar na figura o edema tanto no sentido distal quanto proximal do local da picada (marcar com P - local da picada)

13.8.3 **Linfonodos** ☐ ausente ☐ presente Tamanho (cm): _______

Consistência ☐ elástica ☐ pétrea ☐ amolecida

Sensibilidade ☐ doloroso ☐ indolor

Mobilidade ☐ aderido a planos profundos ☐ não aderido (móvel)

13.8.4 **Dor - classificação numérica na escala de 0 - 10**

Valor*: ☐ Ausente: 0 Leve: 1-3 Moderado: 4-7 Grave: 8-10

* Após uso de analgésico

13.8.5 **Tonalidade Perilesional:** 1-Não 2-Sim

☐ normal ☐ violácia ☐ vermelhidão ☐ necrótica (enegrecida)

13.8.6 **Características da Pele:** 1-Não 2-Sim

☐ equimose ☐ bolhas ☐ secreção serosa ☐ somente a marca da presa

13.8.7 Temperatura: Área acometida _____°C 13.8.8 Área contralateral _____°C

13.9 Manifestações Sistêmicas: 1-Não 2-Sim, marcar um X

☐ Anúria ☐ Cefaleia ☐ Choque ☐ Cólicas abdominiais ☐ Convulsão

☐ Diarreia ☐ Enterorragia(melena e hematoquesia) ☐ Epistaxe

☐ Equimose ☐ Gengivorragia ☐ Hematêmese ☐ Hematúria

☐ Hemoptise ☐ Hemorragia conjuntival ☐ Hipermenorragia ☐ Náuseas

☐ Oligúria ☐ Otorragia ☐ Petéquias ☐ Sudorese ☐ Vômito

☐ Outras:____

Assinatura e Carimbo: ____

14. Dados da lesão do acidente ofídico

14.1 Infecção Secundária ao Acidente Ofídico: ☐ 1-Não 2-Sim

14.2 Data do diagnóstico: ____/____/____ **14.3 Hora**: ____:____

14.4 Manifestações Locais: 1-Não 2-Sim

☐ celulite ☐ abscesso ☐ Outro (Espec.): ______________________________

14.5 Antibioticoterapia: ______________________ **Posologia:** ________________

Fotodocumentação**: *Favor tirar uma foto da lesão antes da retirada da amostra para exame.***

Assinatura e Carimbo:____

15. Dados clínicos do paciente - Dia 7 (Retorno Ambulatorial)

15.1 Data da avaliação: |__|__||__|__||__|__| **15.2 Hora**:____:____

15.3 Pressão arterial |__|__|__|/|__|__|__| mm Hg **15.4 Pulso** |__|__|__| por min

15.5 Temp. |__|__|.|__| °C **15.6 Freq. Resp.** |__|__| rpm

15.7 Queixas concomitantes

☐ 1-Não 2-Sim (Espec.):____

15.8 Manifestações Locais: 1-Não 2-Sim

15.8.1 **Sangramento** ☐ sangramento em pele ou mucosas

☐ equimose evidentes

☐ sangramento sem comprometimento do estado geral

15.8.2 **Edema** ☐ Leve - edema local de até 2 segmentos

☐ Moderado - edema local de 3 a 4 segmentos

☐ Grave - edema local de 5 segmentos

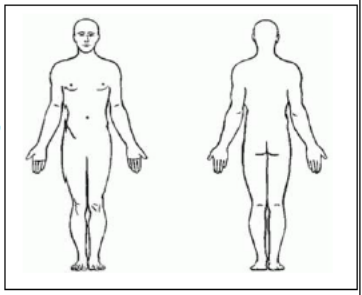

- Circunferência da região acometida (Centímetros)

Lado direito: _____cm Lado esquerdo: ____cm região ímpar: ____cm

Extensão do edema: _____cm*

* marcar na figura o edema tanto no sentido distal quanto proximal do local da picada (marcar com P - local da picada)

15.8.3 **Linfonodos** ☐ ausente ☐ presente Tamanho (cm): _______

Consistência ☐ elástica ☐ pétrea ☐ amolecida

Sensibilidade ☐ doloroso ☐ indolor

Mobilidade ☐ aderido a planos profundos ☐ não aderido (móvel)

15.8.4 **Dor - classificação numérica na escala de 0 - 10**

Valor*: ☐ Ausente: 0 Leve: 1-3 Moderado: 4-7 Grave: 8-10

* Após uso de analgésico

15.8.5 **Tonalidade Perilesional:** 1-Não 2-Sim

☐ normal ☐ violácia ☐ vermelhidão ☐ necrótica (enegrecida)

15.8.6 **Características da Pele:** 1-Não 2-Sim

☐ equimose ☐ bolhas ☐ secreção serosa ☐ somente a marca da presa

15.8.7 Temperatura: Área acometida _____°C 15.8.8 Área contralateral _____°C

15.9 Manifestações Sistêmicas: 1-Não 2-Sim, marcar um X

☐ Anúria ☐ Cefaleia ☐ Choque ☐ Cólicas abdominiais ☐ Convulsão

☐ Diarreia ☐ Enterorragia(melena e hematoquesia) ☐ Epistaxe

☐ Equimose ☐ Gengivorragia ☐ Hematêmese ☐ Hematúria

☐ Hemoptise ☐ Hemorragia conjuntival ☐ Hipermenorragia ☐ Náuseas

☐ Oligúria ☐ Otorragia ☐ Petéquias ☐ Sudorese ☐ Vômito

☐ Outras:____

Assinatura e Carimbo: ____

16. Dados da lesão do acidente ofídico

16.1 Infecção Secundária ao Acidente Ofídico: ☐ 1-Não 2-Sim

16.2 Data do diagnóstico: ____/____/____ **16.3 Hora**: ____:____

16.4 Manifestações Locais: 1-Não 2-Sim

☐ celulite ☐ abscesso ☐ Outro (Espec.): ____________________________

16.5 Antibioticoterapia: ______________________ **Posologia:** ________________

<u>Fotodocumentação</u>: Favor tirar uma foto da lesão antes da retirada da amostra para exame.

Assinatura e Carimbo:____

17. Exames Laboratoriais

17.1 Sangue

	DADOS	D1	D2	D3	D4	D7	UN.	REF.
ERITROGRAMA	HEMÁCIAS						milhões/mm^3	4.7 A 6.1
	HEMOGLOBINA						G/DL	13,0 A 16,0
	HEMATÓCRITO						%	40,0 A 52,0
	M.C.V.						FL	80 A 97
	M.C.H.						PG	27,0 A 31,0
	M.C.H.C						G/DL	33,0 A 37,0
	R.D.W.						%	11,5 A 14,5%
LEUCOGRAMA	LEUCÓCITOS						/ mm^3	4.000-10.800
	SEGMENTADOS						%/ mm^3	42,1 A 75,2
	MIELÓCITOS						%/ mm^3	
	METAMIELÓCITOS						%/ mm^3	
	BASTÕES						%/ mm^3	
	EOSINÓFILOS						%/ mm^3	0,0 A 3,0
	BASÓFILOS						%/ mm^3	0,0 A 3,0
	LINFÓCITOS						/ mm^3	20,0 A 51,1
	MONÓCITOS						/ mm^3	
PLAQUETO -GRAMA	PLAQUETAS						/ mm^3	130.000-400.000
	M.P.V.						FL	7,40 A 10,4
	P.C.T							
	P.D.W.							
COAGULO-GRAMA	TC						MINUTOS	ATE 10
	FIBRINOGÊNIO						G/DL	2 A 4
	TAP						"/%	13,5"= 100%
	INR							1,0
Bioquímica	SÓDIO							135 a 145 mmol/L
	POTÁSSIO							Adultos: 3,5 a 5,1 mmol/L
	URÉIA							Adultos 15 – 39 2,5 – 6,4<br>>60 anos 17 – 45 2,9 – 7,5
	CREATININA							Homens<br>Mulheres<br>Inferior a 1,2<br>Inferior a 1,1
Marcadores Inflamatórios	CK							
	CK-MB							
	Proteína C reativa							
	VHS							
	DHL							
	AST							
	ALT							

17.1.1 Sangue – Alíquotas (Laboratório UFAM)

Laboratório UFAM	TNF-α							
	CCL-2							
	CXCL-8							
	TGF-β							
	IL-1β							
	IL-6							
	IL-10							
	Dot-Elisa (Venenemia)							

17.2 Urina

Carac. Gerais	VOL							
	COR							amarelo citrino
	ASPECTO							Lí Mpido
	pH							4,5 – 7,8
	DENSIDADE							1,005 – 1,030
Bioquímica	GLICOSE							negativo
	PROTEÍNAS							negativo
	CORPOS CETÔNICOS							negativo
	BILIRRUBINA							negativo
	HEMOGLOBINA							Até 0,015 mg
	NITRITO							negativo
	UROBILINOGÊNIO							até 1 mg/dL
Sedimentoscopia	CEL. EPITELIAIS							0 – 1 (pavimentoso)/ campo 400X
	LEUCÓCITOS							0 – 4/campo 400X
	HEMÁCIAS							Homens: 0 – 3/campo 400X Mulheres: 0 – 5/campo 400X
	CRISTAIS							
	CILINDROS							0 – 1 (hialino)/campo 100X
	BACTERIÚRIA							Ausente ou escassa

18. INFECÇÃO LOCAL: ☐ 1-Não 2-Sim

18.1 Tipo de amostra ☐ 1- Hemocultura 2-Aspirado da Lesão 3- Biópsia

18.2 Data da amostra: |__|__|__|__|__|__|

18.3 Bactérias Identificadas 1-Não 2-Sim, marcar um X

☐ não identificado ☐ *Staphylococcus aureus* ☐ *Enterococcus faecalis*

☐ *Streptococcus spp* ☐ *Escherichia coli* ☐ *Klebsiella pneumoniae*

☐ *Proteus spp* ☐ *Morganella morganii* ☐ *Pseudomonas aeruginosa*

☐ *Acinetobacter spp* ☐ Enterobacter spp ☐ *Citrobacter ssp*

☐ *Providencia rettgeri* ☐ *Salmonella entérica diarizonae*

☐ Outra: ____

Antibiograma

18.4 Sensível a: 1-Não 2-Sim 9-Não testado

☐ Amicacina ☐ Ampicilina ☐ Amoxicilina/Ácido Clavulânico

☐ Cefalexina ☐ Cefalotina ☐ Cefepima ☐ Cefoxitina

☐ Cefotaxima ☐ Ceftazidima ☐ Ceftriaxona ☐ Ciprofloxacino

☐ Clindamicina ☐ Cloranfenicol ☐ Eritromicina ☐ Gentamicina

☐ Ertapenem ☐ Imipenem ☐ Meropenem ☐ Neomicina

☐ Oxacilina ☐ Penicilina ☐ Piperacilian ☐ Tazobactam

☐ Tetraciclina ☐ Vancomicina ☐ Sulfametoxazol/trimetropim

☐ Outro: ___________________________________

18.5 Resistente a: 1-Não 2-Sim 9-Não testado

☐ Amicacina ☐ Ampicilina ☐ Amoxicilina/Ácido Clavulânico

☐ Cefalexina ☐ Cefalotina ☐ Cefepima ☐ Cefoxitina

☐ Cefotaxima ☐ Ceftazidima ☐ Ceftriaxona ☐ Ciprofloxacino

☐ Clindamicina ☐ Cloranfenicol ☐ Eritromicina ☐ Gentamicina

☐ Ertapenem ☐ Imipenem ☐ Meropenem ☐ Neomicina

☐ Oxacilina ☐ Penicilina ☐ Piperacilian ☐ Tazobactam

☐ Tetraciclina ☐ Vancomicina ☐ Sulfametoxazol/trimetropim

☐ Outro: ____________________________________

19. Avaliação Final

19.1 Última adm. da medicação do estudo |__|__||__|__||__|__|

19.2 Completou todos os procedimentos ☐ 1-Não 2-Sim

Se não, Especifique:________________________________ .....

20. Após 30 (trinta) dias do acidente ofídico

20.1 Data: |__|__||__|__||__|__|

20.2 Contato telefônico: ☐ 1-Não 2-Sim

Se não, Especifique:________________________________

20.3 Avaliação: 1-Não 2-Sim 9-Ignorado

☐ cicatrização completa da ferida ☐ infecção da lesão ☐ debridamento

☐ amputação de membro ☐ sem alteração

Observação: ____

____

____
