# Supplementary material for: Poor efficacy of preemptive amoxicillin clavulanate for preventing secondary infection from Bothrops snakebites in the Brazilian Amazon: A randomized controlled clinical trial
Source: PLoS Negl Trop Dis. 2017 Jul 10;11(7):e0005745. doi: 10.1371/journal.pntd.0005745 (PMC5519217; doi:10.1371/journal.pntd.0005745)
Supplement: S2 Appendix — (PDF) [file pntd.0005745.s006.pdf]

PARECER CONSUBSTANCIADO DO CEP

DADOS DO PROJETO DE PESQUISA

Título da Pesquisa: Antibioticoterapia empírica precoce: um ensaio clínico randomizado para avaliação de superioridade de ciprofloxacina versus placebo em infecção bacteriana secundária de pacientes vítimas de acidentes ofídicos na Amazonia Brasileira

Pesquisador: Jacqueline de Almeida Gonçalves Sachett
Área Temática:
Versão: 2
CAAE: 19380913.6.0000.5016
Instituição Proponente: Escola Superior de Ciências da Saúde da Universidade do Estado do
Patrocinador Principal: Financiamento Próprio

DADOS DO PARECER

Número do Parecer: 492.892
Data da Relatoria: 13/12/2013

Apresentação do Projeto:
Antibioticoterapia empírica precoce: um ensaio clínico randomizado para avaliação de superioridade de ciprofloxacina versus placebo em o infecção bacteriana secundária de pacientes vítimas de acidentes ofídicos na Amazonia Brasileira.Trata-se de um ensaio clínico com pacientes envolvidos em acidentes ofídicos atendidos na Fundação de Medicina Tropical Doutor Heitor Vieira Dourado, acometidos por acidentes ofídicos no período de novembro/2013 a maio/2015. A Fundação de Medicina Tropical Doutor Heitor Vieira Dourado está situada no município de Manaus-AM, sendo considerada centro de referência nacional e mundial para o tratamento de enfermidades tropicais, principalmente aos eventos relacionados a animais peçonhentos. A população considerada para o presente estudo corresponde aos pacientes atendidos pela instituição com acidentes ofídicos,
correspondendo em média 300 pacientes/ano. Assim, será considerado para o cálculo da população para um período de nove mesmo de seleção amostral.

Objetivo da Pesquisa:
Objetivo Primário:
- Avaliar a eficácia da antibioticoterapia empírica precoce na prevenção de infecções secundários

em pacientes vítimas de acidentes ofídicos.

Objetivo Secundário:

- Identificar o perfil sócio-demográfico dos pacientes envolvidos em acidentes ofídicos;- Utilizar antibioticoterapia empírica precoce em um grupo de

pacientes pós acidente ofídico; - Verificar a evolução clínica relacionada à infecção secundária de pacientes envolvidos em acidente ofídico tratados com e sem antibioticoterapia.- Estabelecer a infectividade das serpentes na lesão decorrentes do acidente e o escore clínico para definição destas

infecções.

Avaliação dos Riscos e Benefícios:

Riscos:

Os riscos são mínimos por tratar de avaliação clínica sem teste de novas drogas, se trata de utilização de antibioticoterapia empírica precoce.

Benefícios:

Este estudo propiciará a participação direta dos profissionais de saúde atuantes no hospital referência em medicina tropical do estado do Amazonas, valorizando o atendimento da rede de assistência específica de ofidismo na região. Além disso, contribuirá para a diminuição da incidência de infecções secundárias pós-acidente ofídico, redução de gastos com internação hospitalar e tratamento dessas infecções, prevenção de resistência

bacteriana ao estabelecer padronização da antibioticoterapia, diminuição dos agravos decorrentes desses acidentes e devolução precoce do indivíduo às suas atividades laborais pelo fato da maioria destes eventos ocorrerem em trabalhos ativos da área rural.

Comentários e Considerações sobre a Pesquisa:

O estudo contribuirá para o tratamento mais apropriado para os acidentes ofídicos na Amazônia.

Considerações sobre os Termos de apresentação obrigatória:

Todos os termos foram apresentados.

Recomendações:

Sem recomendações.

Conclusões ou Pendências e Lista de Inadequações:

Sem pendências.

Situação do Parecer:

Aprovado

Endereço: Av. Djalma Batista, nº 3578, Chapada
Bairro: chapada **CEP:** 69.050-030
UF: AM **Município:** MANAUS
Telefone: (92)3878-4368 **Fax:** (92)3878-4368 **E-mail:** cep.uea@gmail.com

Necessita Apreciação da CONEP:

Não

Considerações Finais a critério do CEP:

MANAUS, 13 de Dezembro de 2013

Assinador por:
Manoel Luiz Neto
(Coordenador)

Prof. M.Sc. Manoel Luiz Neto
Coordenador
Comitê de Ética em Pesquisa
ESA / UEA

Endereço: Av. Djalma Batista, nº 3578, Chapada
Bairro: chapada **CEP:** 69.050-030
UF: AM **Município:** MANAUS
Telefone: (92)3878-4368 **Fax:** (92)3878-4368 **E-mail:** cep.uea@gmail.com

PARECER CONSUBSTANCIADO DO CEP

Elaborado pela Instituição Coparticipante

DADOS DO PROJETO DE PESQUISA

Título da Pesquisa: Antibioticoterapia empírica precoce: um ensaio clínico randomizado para avaliação de superioridade de ciprofloxacina versus placebo em infecção bacteriana secundária de pacientes vítimas de acidentes ofídicos na Amazonia Brasileira

Pesquisador: Jacqueline de Almeida Gonçalves Sachett
Área Temática:
Versão: 2
CAAE: 19380913.6.3001.0005
Instituição Proponente: Escola Superior de Ciências da Saúde da Universidade do Estado do
Patrocinador Principal: Financiamento Próprio

DADOS DO PARECER

Número do Parecer: 602.907-0
Data da Relatoria: 20/12/2013

Apresentação do Projeto:
conforme parecer # 492.892, de 13/12/2013.

Objetivo da Pesquisa:
conforme parecer # 492.892, de 13/12/2013.

Avaliação dos Riscos e Benefícios:
conforme parecer # 492.892, de 13/12/2013.

Comentários e Considerações sobre a Pesquisa:
conforme parecer # 492.892, de 13/12/2013.

Considerações sobre os Termos de apresentação obrigatória:
conforme parecer # 492.892, de 13/12/2013.

Recomendações:

Conclusões ou Pendências e Lista de Inadequações:
Trata-se de projeto de pesquisa cuja instituição proponente é a Universidade do Estado do Amazonas e a FMT-HVD é a instituição coparticipante.

Endereço: Av. Pedro Teixeira, 25
Bairro: D. Pedro I **CEP:** 69.040-000
UF: AM **Município:** MANAUS
Telefone: (92)2127-3572 **Fax:** (92)2127-3572 **E-mail:** cep@fmt.am.gov.br

Pelo o que está exposto no protocolo de pesquisa e seus apêndices, vota-se pela aprovação do mesmo.

Situação do Parecer:
Aprovado

Necessita Apreciação da CONEP:
Não

Considerações Finais a critério do CEP:
O presente projeto está APROVADO e os interessados ficam informados de apresentar a este CEP os relatórios parciais e final do estudo, conforme prevê a Resolução CNS nº 466/2012, utilizando os modelos disponíveis em nossa homepage, na aba ¿Modelos de Documentos¿. Se houver qualquer tipo de dúvida o pesquisador deve entrar em contato com o CEP/FMT-HVD, que lhe oferecerá as orientações necessárias.

MANAUS, 12 de Abril de 2014

Assinador por:
Maria Paula Gomes Mourão
(Coordenador)

Este parecer reemitido substitui o parecer número 602907 gerado na data 10/04/2014 15:50:39, onde o número CAAE foi alterado de 19380913.6.0000.5016 para 19380913.6.3001.0005.

PARECER CONSUBSTANCIADO DO CEP

DADOS DO PROJETO DE PESQUISA

Título da Pesquisa: Antibioticoterapia empírica precoce: um ensaio clínico randomizado para avaliação de superioridade de ciprofloxacina versus placebo em infecção bacteriana secundária de pacientes vítimas de acidentes ofídicos na Amazonia Brasileira

Pesquisador: Jacqueline de Almeida Gonçalves Sachett

Área Temática:

Versão: 2

CAAE: 19380913.6.0000.5016

Instituição Proponente: Escola Superior de Ciências da Saúde da Universidade do Estado do

Patrocinador Principal: Financiamento Próprio

DADOS DA NOTIFICAÇÃO

Tipo de Notificação: Outros

Detalhe: solicitação de alteração o medicamento utilizado

Justificativa: Venho por meio desta solicitar alteração no projeto intitulado ¿Antibioticoterapia

Data do Envio: 14/05/2014

Situação da Notificação: Parecer Consubstanciado Emitido

DADOS DO PARECER

Número do Parecer: 677.472

Data da Relatoria: 26/05/2014

Apresentação da Notificação:

Venho por meio desta solicitar alteração no projeto intitulado Antibioticoterapia empírica precoce: um ensaio clínico randomizado para avaliação de superioridade de ciprofloxacina versus placebo em infecção bacteriana secundária de pacientes vítimas de acidentes ofídicos na Amazônia Brasileira e aprovado sob o n° CAAE:19380913.6.0000.5016. Após intensas reuniões com um painel de especialistas das áreas de dermatologia, infectologia, patologia, farmacologia, hematologia e clínica médica, houve a constatação de que a antibioticoterapia empírica precoce com a utilização do medicamento Ciprofloxacino possui espectro de ação um pouco restrito, necessitando associação de outras drogas para uma atuação mais satisfatória. Com isso, optou-se

para a utilização de inibidor da betalactamase Clavulin BD, por se tratar de maior espectro de ação contra bactérias aeróbias e anaeróbias, gram positivas e negativas.

Objetivo da Notificação:

Alterar a terapia medicamentosa com ciprofloxacina para Clavulin inibidor da betalactamase por se tratar de maior espectro de ação contra bactérias aeróbias e anaeróbias, gram positivas e negativas.

Avaliação dos Riscos e Benefícios:

conforme parecer # 492.892, de 13/12/2013.

Comentários e Considerações sobre a Notificação:

conforme parecer # 492.892, de 13/12/2013.

Considerações sobre os Termos de apresentação obrigatória:

conforme parecer # 492.892, de 13/12/2013.

Recomendações:

conforme parecer # 492.892, de 13/12/2013.

Conclusões ou Pendências e Lista de Inadequações:

conforme parecer # 492.892, de 13/12/2013.

Situação do Parecer:

Aprovado

Necessita Apreciação da CONEP:

Não

Considerações Finais a critério do CEP:

Parecer aprovado de acordo com a resolucao 466 de 12 de Dezembro de 2012 do CONEP

MANAUS, 06 de Junho de 2014

Assinado por:
Manoel Luiz Neto
(Coordenador)

Prof. M.Sc. Manoel Luiz Neto
Coordenador
Comitê de Ética em Pesquisa
ESA / UEA

Endereço: Av. Djalma Batista, nº 3578, Chapada
Bairro: chapada **CEP:** 69.050-030
UF: AM **Município:** MANAUS
Telefone: (92)3878-4368 **Fax:** (92)3878-4368 **E-mail:** cep.uea@gmail.com
